# Molecular Characterization of Complete Simian Foamy Virus Genomes from Three Colobine Monkeys Reveals Highly Divergent Evolutionary Trajectories and Identifies Transmission to Humans

**DOI:** 10.3390/v18030320

**Published:** 2026-03-04

**Authors:** Anupama Shankar, Haoqiang Zheng, David Cowan, Hongwei Jia, Gunars Osis, Alex Burgin, Mili Sheth, Nicole A. Hoff, Megan Halbrook, Anne W. Rimoin, Tony L. Goldberg, Colin A. Chapman, Nelson Ting, William M. Switzer

**Affiliations:** 1 Laboratory Branch, Division of HIV Prevention, National Center for HIV, Viral Hepatitis, STD, and TB Prevention, Centers for Disease Control and Prevention, Atlanta, GA 30329, USA; hxz2@cdc.gov (H.Z.); ryf7@cdc.gov (D.C.); hbj8@cdc.gov (H.J.); 2 ASRT Inc., Contractor for Office of Laboratory Systems and Response, Centers for Disease Control and Prevention, Smyrna, GA 30080, USA; ukm9@cdc.gov; 3 Biotechnology Core Facility Branch, Division of Core Laboratory Services and Response, Office of Laboratory Systems and Response, Centers for Disease Control and Prevention, Atlanta, GA 30329, USA; owz6@cdc.gov (A.B.); vgg1@cdc.gov (M.S.); 4 Department of Epidemiology, UCLA Fielding School of Public Health, University of California at Los Angeles, Los Angeles, CA 90095, USA; nhoff84@ucla.edu (N.A.H.); meganhalbrook@ucla.edu (M.H.); arimoin@g.ucla.edu (A.W.R.); 5 Department of Pathobiological Sciences, School of Veterinary Medicine, University of Wisconsin-Madison, Madison, WI 53706, USA; tony.goldberg@wisc.edu; 6 Biology Department, Vancouver Island University, Nanaimo, BC V9R 5S5, Canada; colin.chapman.research@gmail.com; 7 School of Life Sciences, University of KwaZulu-Natal, Pietermaritzburg 3201, South Africa; 8 Shaanxi Key Laboratory for Animal Conservation, Northwest University, Xi’an 710069, China; 9 Institute of Ecology and Evolution, University of Oregon, Eugene, OR 97403, USA; nting@uoregon.edu

**Keywords:** zoonotic infection, simian foamy viruses (SFV), genomes, evolution, diagnostics, host switching, colobinae

## Abstract

Simian foamy viruses (SFVs) are ancient retroviruses that co-evolve with nonhuman primates (NHPs), although genomic data from Asian and African monkeys are limited. We report the characterization of three new SFV colobine genomes from two Asian species (*Trachypithecus francoisi* (Tfr) and *Pygathrix nemaeus* (Pne)) and one African monkey (*Colobus guereza*, Cgu), obtained via metagenomics analysis of peripheral blood leukocyte tissue culture isolates. Genomic analyses found conserved structural, enzymatic, and auxiliary genes flanked by long terminal repeats, with all major transcriptional and structural motifs highly preserved. An in-frame Δtas mutation in tissue culture and ex vivo specimens was identified in the SFVpne genome, which may promote viral latency. Phylogenetic analyses revealed that these colobine SFVs have distinct evolutionary trajectories without clustering together, contradicting a strict virus–host co-evolution. We developed a new generic SFV PCR assay using these genomes with increased detection sensitivity for Colobinae SFVs and identified four new human infections with Cgu-derived SFV in the Democratic Republic of Congo. Our findings indicate that SFV evolution in colobine monkeys is shaped by host switching, cross-species transmission, and high viral diversity. Our study underscores the importance of broadening SFV genomic sampling to better understand viral evolution, zoonotic risk, and improved diagnostic capabilities.

## 1. Introduction

Simian foamy viruses (SFVs) are complex retroviruses in the family *Retroviridae*, subfamily *Spumaretrovirinae*, and genus *Simiispumavirus*, infecting a wide range of nonhuman primates (NHPs) across Asia, Africa, and Latin America [1,2,3,4,5,6,7,8,9,10,11,12,13,14,15]. SFVs exhibit genetic stability with very low evolutionary rates, having co-evolved with their NHP hosts for millions of years, resulting in species-specific lineages. SFVs establish lifelong persistent infections, often without causing disease, likely because of adaptation during their co-evolutionary history [14]. SFV infection is characterized by persistent seropositivity and molecular detection in peripheral blood lymphocytes (PBLs) and other body sites, including the oral cavity, where viral replication occurs [14]. Transmission of SFV typically occurs through saliva during aggressive encounters, such as bites and scratches, which occur during primate social interactions [14].

Although no human-specific SFV has been identified, zoonotic transmission frequently occurs through contact with NHPs during hunting for bushmeat, biomedical research, or in zoological collections [16,17,18]. In such cases, phylogenetic analyses have exploited the co-evolutionary history of SFVs to identify the NHP source of infection [17,18,19]. Human SFV infections have originated from a range of Old-World NHPs, including chimpanzees, gorillas, baboons, mandrills, African green monkeys, and various macaque species [13,17,18,19,20,21,22,23]. Like the simian hosts, SFV infections in humans appear nonpathogenic, although this conclusion is biased by studying healthy populations with limited clinical follow-up of SFV-infected individuals [24,25]. For example, a recent cross-sectional case–control study of asymptomatic male hunters from Cameroon infected with gorilla SFV reported anemia and hematological abnormalities, though the clinical significance remains unclear [25]. As with other retroviruses, such as simian and human immunodeficiency viruses (SIV and HIV) and T-cell lymphotropic viruses, disease may take decades to develop [26,27,28,29]. Hence, SFV-associated diseases may be rare, cryptic, or restricted to infections with specific viral variants [18].

Research on SFV evolutionary relationships in Old-World monkeys has primarily focused on African species, with limited representation from Asia, mainly involving *Macaca* species due to their prevalence in zoo collections, research studies, and interactions with humans near temples [4,30,31,32,33,34]. The Colobinae subfamily, known as colobines or leaf-eating monkeys, is a diverse group of Old-World primates found across equatorial Africa (Colobini tribe) and Asia (Presbytini tribe), having diverged from the Cercopithecinae approximately 13.8 million years ago (MYA) [35,36]. Although the taxonomy of specific Colobinae clades remains debated, this subfamily includes the African genera *Colobus* (black and white colobus)*, Procolobus* (olive colobus)*, Piliocolobus* (red colobus), the Asian genera *Presbytis* (surilis), *Trachypithecus* (lutung, langur, or leaf monkey), *Semnopithecus* (gray langur), *Rhinopithecus* (snub-nosed monkey), *Pygathrix* (douc langur), *Nasalis* (proboscis monkey), and *Simias* (pig-tailed langur). Asia has 57 colobine species compared to 23 in Africa [35].

Previously, we demonstrated that SFVs from captive *Colobus guereza* and wild *Piliocolobus rufomitratus tephrosceles* from Uganda and *P. badius badius* from Côte d'Ivoire clustered phylogenetically within the *Cercopithecinae* as sister taxa to the *Macaca* SFV by using short integrase (IN) sequences obtained by generic PCR-amplification of PBL DNA [3]. While these results indicate genetic relatedness of the African Colobinae SFV, the co-evolutionary hypothesis suggests these colobus SFVs should cluster outside the SFVs of the *Cercopithicinae*. In contrast, only one SFV *pol* sequence has been reported from the Asian colobine *Trachypithecus francoisi* (Francois’ langur), which formed a distinct and highly divergent phylogenetic lineage between the ape and *Cercopithicinae* SFVs, consistent with the co-evolutionary hypothesis for the Colobinae hosts and SFVs [37]. The *T. francoisi* SFV sequence was obtained with generic PCR amplification from tissue culture cells, as all SFV western blot (WB)-positive Francois’ langurs and *Pygathrix nemaeus* (red-shanked douc langur) PBL DNA samples in that study were PCR-negative, further highlighting the high divergence of Asian colobine SFVs [37].

In the Democratic Republic of Congo (DRC), we demonstrated human infection with a novel colobus SFV from *C. angolensis* (Angolan colobus) in two of sixteen WB-positive women [13]. Among eleven WB-positive persons in the study with available PBL DNA, ten reported NHP exposure, including eight with contact with *C. angolensis*, but were negative for SFV using generic PCR assays [13]. Similarly, in Asia, individuals are exposed to NHPs, including langurs and macaques, through activities related to deforestation, agricultural expansion, hunting, and when sharing urban settings like parks, religious sites, animal markets, and zoos [4,38,39]. Two studies reported SFV infections from *Macaca* species in four persons in Asian countries who lived or worked near NHPs [38,39]. In one of these studies, five persons were WB-positive but PCR-negative using a generic SFV *pol* assay [39]. These negative PCR results in both Africa and Asia likely reflect viral loads below the detection threshold of the molecular assays or infections with divergent SFVs not readily identified with the currently used PCR methods. While PCR negativity alone does not demonstrate viral divergence, this pattern is consistent with substantial primer–template mismatch caused by sequence divergence, particularly given the successful amplification of SFV sequences from tissue culture isolates derived from the same animals. The generic *pol* PCR assays used in these studies were designed based on complete SFV genomes available at the time, including chimpanzee, gorilla, macaque, and African green monkey. While effective for detecting various SFVs from multiple species, these assays may not identify highly divergent SFVs, as demonstrated with gibbon SFV [40].

A database containing sequences from divergent SFV lineages is crucial for the molecular surveillance of SFVs in NHPs and zoonotically infected humans. Currently, the only complete SFV genomes from Asian NHPs are from an orangutan (*Pongo pygmaeuspygmaeus*, SFVppy_bella, GenBank # AJ544579), a pileated gibbon (SFVhpi_SAM106, *Hylobates pileatus*, GenBank # MF621235), and five macaques (SFVmcy _FV21, *M. cyclopis*, GenBank # NC_010819; SFVmcy _FV34[RF], *M. cyclopis*, GenBank # KF026286; SFVmfa_Cy5061, *M. fascicularis*, GenBank # KF026286; SFVmfu_WK1.pJM356, *M. fuscata*, GenBank # AB92351; and SFVmmu_K3T, *M. mulatta*, GenBank # MF280817). No complete SFV genomes from Asian or African Colobinae exist, despite their significant taxonomic diversity and wide geographic distribution. To address this gap and improve diagnostic assays for detecting Colobinae SFV in humans, we obtained and characterized complete SFV genomes from one African (*Colobus guereza*, Cgu) and two Asian species (*Trachypithecus francoisi* (Tfr) and *Pygathrix nemaeus* (Pne)). We used these new SFV genomes to investigate their evolutionary histories through detailed phylogenetic analyses, design generic PCR assays for their detection, and apply these new assays on PBL DNA samples from SFV-seropositive persons in the DRC with NHP exposure.

## 2. Methods

*Blood Sample Processing, Serology, Co-Culture, and PCR Detection.* EDTA-treated whole blood specimens were collected from captive colobine monkeys at North American zoological gardens during their annual examinations, following the guidelines of animal care and use committees at each institution. PBLs and plasma were obtained by Ficoll-Hypaque centrifugation of whole blood, and DNA lysates were prepared as described previously [37]. Human DNA specimens from persons in the DRC were extracted from buffy coats using the Flexigene DNA extraction kit (Qiagen) and quantified with a Nanodrop instrument [11]. The UCLA Institutional Review Board approved the collection, storage, and future testing of blood samples collected in 2007 from all consenting study participants as previously described [11]. This study (Protocol #07041) was reviewed by the Centers for Disease Control (CDC), deemed research not involving human subjects, and conducted consistently with applicable federal law and CDC policy using anonymized participant specimens and information. Demographic and animal contact information for DRC participants was collected with study questionnaires.

SFV serology was performed on plasma using a combination of validated EIA and western blot (WB) assays that broadly detect SFV in Old-World monkeys and apes [30,37]. SFV was isolated from three seropositive colobines (Cgu_910916, Pne_500057, and Tfr_083616) by co-culturing peripheral blood lymphocytes (PBLs) with canine thymocyte (Cf2Th) cells as previously described [37]. In brief, cryopreserved PBLs were rapidly thawed and maintained in interleukin-2-supplemented medium at 37 °C for 72 h. Following stimulation, cells were washed and combined with Cf2Th cells at a 1:1 ratio. The co-cultures were examined at 3–4 day intervals for the development of syncytial cytopathic effects (CPE) characteristic of SFV infection. Upon confirmation of CPE, Cf2Th cells and corresponding culture supernatants were harvested and preserved in liquid nitrogen.

The integrity of PBL and buffy coat DNA was confirmed by β-actin PCR [11]. Initial SFV PCR testing was conducted using 1.0 µg of PBL or 100 ng of tissue culture DNA in a generic nested *pol* PCR assay designed to amplify a 465 bp IN region that has been used successfully to detect diverse SFVs [12,37]. This assay amplified integrase (IN) sequences from Cgu_910916 PBL and Tfr_083616 tissue culture DNA, but not from Pne_500057 [37,41]. We then developed a new nested PCR assay in IN based on a multiple sequence alignment, which included newly acquired complete genomes from the three colobine monkeys and those from 28 other Old-World monkeys and apes. The IN region was selected because it allows broad amplification of diverse SFVs while providing sufficient phylogenetic resolution to distinguish host-associated lineages and is widely used for comparative evolutionary analyses of foamy viruses. The first round PCR used 200 ng DNA with primers SFV1M 5′-GAY AAR CTT GCC ACC CAA GG-3′ and FVPGR1M 5′-CCT GYA RAA GAG ANA RYT CYT CTT CTC-3′ with 3.4 units Expand High Fidelity Taq polymerase (Roche, Indianapolis, IN) per reaction under the conditions of 95 °C for 30 s, 53 °C for 30 s, and 72 °C for 2 min for 40 cycles. The 2nd round PCR used primers SIF3M 5′-CCA ARC CTG GAT GCA GAG YTG GAT C-3′ and FVPGR2M 5′-TCT TCT CKN GWY AAR TCA AGT GT-3′ with 2.5 units AmpliTaq (Thermo Fisher Scientific, Waltham, MA, USA) per reaction under the conditions of 95 °C for 30 s, 50 °C for 30 s, and 72 °C for 2 min for 40 cycles. The primary and nested PCR product sizes were 950 bp and 854 bp, respectively. The sensitivity of the new PCR assay was estimated using 10-fold titrations of nucleic acids from PBL tissue culture supernatants of SFVcgu_910916, SFVpne_500057, and SFVtfr_083616.

Previous studies indicated that SFV adaptation to tissue culture can cause deletions in the transcriptional transactivator (*tas*) gene, eliminating Tas production and inducing transcription of the Bet (between envelope (*env*) and tas) protein [42,43,44,45]. These *tas* deletion mutants are referred to as Δtas. Overproduction of Bet in the absence of Tas downregulates viral replication, favoring viral persistence and latency [46,47]. We assessed intact and Δtas variants using a new nested PCR assay using primers designed from the SFVpne_500057 genome, employing standard PCR conditions except for a 50 °C annealing temperature and 40 amplification cycles. The outer and inner PCR primers are 10100F 5′-CCA TCA ACA GTA ACC TGG CAC-3′ and 10590R 5′-TCT TGG TAG CGC CGC TTC CTA-3′, and 10165F 5′-GAG AGA TTG GGT ACC TGA TCC-3′ and 10590R 5′-GAG CGA CGT TTT GGG AGT CGA G-3′, respectively.

*Next Generation Sequencing and SFV Genome Assembly.* To obtain complete genomes from viral isolates, we used a metagenomics approach described in detail elsewhere [40]. Briefly, we centrifuged 0.5–1.0 mL of tissue culture supernatant at 43,000 rpm at 4 °C for 30 min and resuspended the viral pellet in 165 μL of supernatant. The sample was treated with a cocktail of DNase enzymes (Turbo DNase, Ambion, Austin, TX, USA; Baseline-ZERO DNase, Biosearch Technologies, Middlesex, UK). Viral nucleic acids were extracted with the QIAamp MinElute Virus Spin Kit (Qiagen, Hilden, Germany) following the manufacturer’s protocol, without the addition of carrier RNA. Purified RNA was reverse-transcribed using random hexamer primers to generate double-stranded cDNA with the SuperScript Double-Stranded cDNA Synthesis Kit (Invitrogen, Carlsbad, CA, USA). The resulting cDNA was cleaned using Agencourt AMPure XP beads (Beckman Coulter, Brea, CA, USA). Approximately 1 ng of purified cDNA was then subjected to concurrent fragmentation and adapter tagging using the Illumina DNA Prep Kit (Illumina, San Diego, CA, USA). Libraries were sequenced on an Illumina MiSeq platform with the MiSeq Reagent Kit v2 (500 cycles), generating paired-end reads in a 2 × 150 bp format.

Illumina’s native demultiplexing software (bcl2fastq v2.20.0.422) was utilized for initial read processing, giving an average read Q score of 33.8. Data was then de-hosted by excluding reads aligned to the dog reference genome (Illumina iGenomes, *Canis familiaris* NCBI build 3.1) because the isolates were grown in Cf2Th cells, using Bowtie2 v2.5.1 [48]. We processed the remaining reads with the nf-core/viralrecon pipeline (v2.6.0, nf-core/viralrecon: nf-core/viralrecon v2.6.0—Rhodium Raccoon) in *de novo* assembly mode, conducting quality control (FastQC), genome assembly using SPAdes [49], Unicycler [50], and Minia [51], and generated an assembly report with QUAST [52]. We analyzed the resulting assemblies with BLASTN to evaluate the content, coverage, and length [53]. Contigs of the appropriate size representing SFV genomes were selected for further analysis, yielding an average mean mapping quality of 38.2 and a mean depth of 24,052 for all contigs.

*Sequence Analysis of Complete SFV Genomes.* We used the DNA-to-protein translation website (http://insilico.ehu.es/translate/; accessed on 31 March 2024, 31 May 2024) to identify protein-coding reading frames in the sense direction of the SFVcgu_910916, SFVpne_500057, and SFVtfr_0836161 genomes.

The boundaries of the complete 5′ and 3′ long terminal repeats (LTRs) were defined by manual inspection, guided by alignment with previously published SFV reference genomes. Putative splice donor and acceptor sites were predicted using the neural network–based NetGene2 platform (http://www.cbs.dtu.dk/services/NetGene2/; accessed on 31 March 2024, 31 May 2024). Candidate nuclear localization signals (NLS) within the Tas protein were assessed using both NucPred (https://nucpred.bioinfo.se/cgi-bin/single.cgi; accessed on 31 March 2024, 31 May 2024) and PSORTII (https://psort.hgc.jp/form2.html; accessed on 31 March 2024, 31 May 2024).

The five principal open reading frames—*gag*, *pol*, *env*, *tas*, and *bet*—were identified and extracted in Geneious v2025.0.3 and aligned against representative monkey SFV genomes with complete sequences. For phylogenetic analyses, a concatenated sequence comprising the three major structural and enzymatic genes (*gag*, *pol*, and *env*) was constructed to enhance analytical robustness.

Codon-aware nucleotide alignments of both the concatenated dataset and individual *pol* sequences were generated with MAFFT v7.0.26 [54], followed by manual curation and removal of gap-containing regions. The optimal nucleotide substitution model was selected using the model-testing function in MEGA v7.0.26, which identified the general time reversible model with gamma-distributed rate variation and a proportion of invariable sites (GTR + G + I) as the best fit. Phylogenetic signal was evaluated through likelihood mapping of quartet topologies in IQ-TREE v1.6.12 [55]. In addition, overall phylogenetic signal from the alignments, as well as substitution saturation, were examined using DAMBE v7.0.35 (http://dambe.bio.uottawa.ca/DAMBE/dambe.aspx, 26 February 2026), identifying saturation at the 3rd codon position in the alignment. Consequently, we used the 1st and 2nd codon positions of the alignment for the concatemer phylogenies as previously described [40].

We inferred the colobine concatemer sequence phylogeny using Bayesian inference with BEAST v.1.8.4, using a birth-death speciation tree prior and the 1st and 2nd codon positions of the concatemer alignment, as we have shown that the 3rd codon position is saturated for phylogenetic analysis [40]. We included SFVs from 19 other simians and three non-simians (feline, bovine, and equine) to explore the full FV phylogeny using 400 million Markov Chain Monte Carlo (MCMC) iterations with a 10% burn-in. We used primate and non-primate fossil and genomic divergence dates to calibrate the relaxed molecular clock as normal or exponential tree priors (Table S1) [36,56,57]. Trees were logged every 40,000 generations, and two independent BEAST runs were performed to verify convergence and reliability of the results. We used Tracer v1.7.2 to confirm convergence, ensuring effective sampling size (ESS) values >250. TreeAnnotator v1.8.4 was applied to select the maximum clade credibility tree from the posterior distribution of 10,001 sampled trees, with a burn-in value of 1000 trees. The *pol* phylogeny was constructed using the approximate ML method with FastTree v2.2.0 (https://morgannprice.github.io/fasttree/, 26 February 2026) and the GTR nucleotide substitution model with clades defined by Shimodaira–Hasegawa (SH) support values ≥0.7. Representative SFV *pol* sequences across primate taxonomy available at GenBank were included for the *pol* phylogenetic analysis. The inferred concatemer and *pol* trees were visualized using FigTree v1.4.4 (http://tree.bio.ed.ac.uk/software/figtree/, 26 February 2026).

Genetic recombination in the concatemer alignment was evaluated using the Recombination Detection Program (RDP) v4.101 using the RDP, GENECONV, MaxChi, Chimaera, Bootscan, 3Seq, and SiScan algorithms [58].

*GenBank Accession Numbers.* The complete genomes of SFVcgu_910916, SFVpne_500057, and SFVtfr_083616 were assigned accession numbers PP966955, PP966956, and PP966957, respectively. The human and monkey SFV *pol* sequences have accession numbers PV939864-PV939903.

Statistical Analysis. We assessed the statistical significance of the proportion of SFV infections detected by the original and new *pol* PCR assays using a two-tailed Z score test at a significance level of 0.01 at https://www.socscistatistics.com/tests/ztest/default2.aspx; accessed on 31 November 2025.

## 3. Results

*SFV genome assembly.* We obtained the complete SFV genomes of SFVcgu_910916, SFVpne_500057, and SFVtfr_083616 using a metagenomics approach with tissue isolate supernatants when ≥50% CPE was observed starting on day 18 of culturing. The three novel SFV genomes were composed of 150,000 to 4.4 million reads, resulting in a mean depth coverage ranging from 1783 to 60,529. The sequences of the complete genomes were determined by manual alignment of overlapping 5′ and 3′ LTR regions to give final lengths of 12,645 bp, 12,649 bp, and 12,644 bp for SFVcgu_910916, SFVpne_500057, and SFVtfr_083616, respectively (Table 1).

*Genome comparisons of the new SFVs with other related Asian and African monkey SFVs identify distinct Colobinae SFV evolutionary histories.* Gene length comparisons of the new SFVs with other monkey SFVs are provided in Table 1. The new SFV genome lengths were closer in length to those from an Asian *Macaca cyclopis* SFV but shorter than those from African monkeys. The lengths of the LTRs and the five coding regions for the three new SFVs were comparable in length to those from African and Asian monkey SFVs. Notably, the SFVpne_500057 LTR and five coding region lengths were closer (LTR and *gag*) or identical (*pol*, *env*, *tas*, *bet*) to those from the *Macaca cyclopis* SFV.

In Table 2, we provide the nucleotide and amino acid identity comparisons of the major genes and proteins, respectively, of the three new SFVs with those from related Asian and African monkey SFVs, and for the LTRs and complete genome nucleotide sequences. Sequence analysis showed that SFVtfr_083616 was nearly equidistant from other monkey SFVs sharing approximately 60% nucleotide identity across the genome. In contrast, SFVcgu_910916 and SFVpne_500057 were closer genetically to SFVcae_LK3 and SFVpan_V909 from African *Chlorocebus* and baboon monkeys, respectively. As expected, the highest identities were seen in the *pol* and *env* genes, and the lowest were seen in the LTR, *tas*, and *bet* regions.

**Figure 1:**
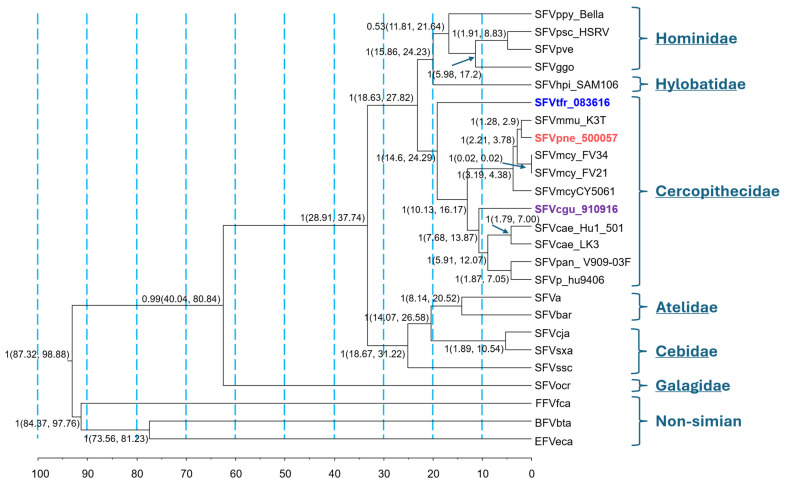
Evolutionary relationships of new Colobinae simian foamy viruses (FVs) to other mammalian FVs using *gag*-polymerase (*pol*)-envelope (*env*) concatemer sequences by using Bayesian inference. **Hominoidea (Great apes):** SFVpve, *Pan troglodytes verus* (Western Africa chimpanzee); SFVpsc, *Pan troglodytes schweinfurthii* (Eastern Africa chimpanzee); SFVggo, *Gorilla gorilla* (gorilla); SFVppy, *Pongo pygmaeus* (orangutan). **Hylobatidae (gibbon):** SFVhpi_Sam106, *Hylobates pileatus* (pileated gibbon). **Cercopithecidae (Old-World monkeys): SFVcgu_910916**, *Colobus guereza* (Mantled guereza or black and white colobus monkey); **SFVtfr_083616**, *Trachypithecus francoisi* (Francois’ monkey); **SFVpne_500057**, *Pygathrix nemaeus nemaeus* (Douc langur); SFVcae_LK3, *Chlorocebus aethiops* (African green monkey); SFVcae_Hu1_501, human infected with SFVcae; SFVmcy_FV21, *Macaca cyclopis* (Formosan rock macaque); SFVmcy_FV34, *Macaca cyclopis*; SFVmfa_CY5061, *Macaca fascicularis* (Long-tailed macaque); SFVmmu_K3T, *Macaca mulatta* (Rhesus macaque); SFVmac, *Macaca mulatta* (Rhesus macaque); SFVpan_V909-03F, *Papio anubis*; SFVp_hu9406, human infected with baboon (*Papio* species) SFV. **Platyrrhini** (**New-World monkeys), Atelidae:** SFVa, *Ateles* species (spider monkey); SFVbar, *Brachyteles arachnoides* (woolly spider monkey). **Cebidae:** SFVcja, *Callithrix jacchus (common marmoset);* SFVssc, *Saimiri sciureus* (squirrel monkey); SFVsxa, *Sapajus xanthosternos* (golden-bellied capuchin). **Galagidae (prosimian):** SFVocr, *Otolemur crassicaudatus* (brown greater galago). **Non-simian mammals:** EFVeca, *Equus caballus* (equine); BFVbta, *Bos taurus* (bovine); and FFVfca, *Felis catus* (feline). The scale bar indicates inferred divergence ages in millions of years. Posterior probabilities are provided at each node of the FV phylogeny, followed by the 95% highest posterior density intervals of the inferred node ages in parentheses.

Bayesian phylogenetic analysis of *gag-pol-env* concatemers inferred divergent evolutionary trajectories for SFVcgu_910916, SFVpne_500057, and SFVtfr_083616, which were polyphyletic instead of forming a Colobinae clade as would be expected under a strict host co-evolution scenario (Figure 1). Only SFVtfr_083616 mirrored the host phylogeny, appearing basal to the African and Asian (Cercopithicinae) monkey SFVs. In contrast, SFVcgu_910916 was basal to the African monkey (Cercopithicini) SFV, while SFVpne_500057 clustered within the macaque SFV clade, likely due to ancestral host switching. Of the remaining FV genomes, only the New-World monkey SFVssc (*Saimiri sciureus*) did not conform to the host co-evolution model, confirming results from previous studies [16,59]. Divergence dating showed that the time to most recent common ancestor (TMRCA) for the Asian SFVtfr_083636 genome was the oldest among Colobinae SFVs (19.12 million years ago (mya)), followed by the African SFVcgu_910916 genome (10.78 mya), with the Asian SFVpne_500057 genome TMRCA evolving more recently within the radiation of the *Macaca* (2.07 mya) (Table 3). Only the SFVcgu_910916 TMRCA aligned with the estimated ancestral colobine divergence of 7.44–16.68 mya; TMRCAs for SFVpne_500057 and SFVtfr_083616 were younger and older, respectively (Table 3). We did not find evidence of recombination in the colobine *gag-pol-env* concatemers using RDP, reducing the likelihood that recombination accounts for the observed gene-tree and TMRCA discordances. Combined, our results suggest that Colobinae SFV evolution has not strictly mirrored host phylogeny and that host switching appears to have influenced SFV phylogeny.

*Conservation of important functional domains in the major coding regions of the new SFVs when compared with related Asian and African monkey SFVs.* An alignment of the complete genomes of SFVcgu_910916, SFVpne_500057, and SFVtfr_083616, provided in Figure S1, shows the location and conservation of the poly adenylation (poly A) signal and TATA boxes in the LTRs. The primer binding site and dimerization signals in the pre-*gag* region are highly conserved, as are the 3′ polypurine tract (PPT) in *pol* and the central PPT preceding the 3′ LTR. The internal promoter in *env* is somewhat conserved. The splice donor (SD) signal in the *tas/bet* coding region is highly conserved, but the inferred splice acceptor (SA) signal for SFVtfr_500057 is distinct and shifted upstream in the alignment by five nucleotides.

Figure S2 shows an alignment of the SFVcgu_910916, SFVpne_500057, and SFVtfr_083616 Gag residues with those from five macaques and five Cercopithecinae SFVs. The PSAP motif important for viral budding; the arginine (R) residue within the N-terminal cytoplasmic targeting and retention signal (CTRS), and the YXXL motif required for particle assembly were completely conserved in all 13 SFVs. We also identified the three glycine-rich (GR) boxes in the C terminus of the Gag protein important for nucleic acid binding and packaging, reverse transcription, and capsid assembly. Within GR2 is the relatively conserved chromatin-binding sequence (CBS) used by the SFV Gag protein to bind to chromosomes for nuclear accumulation in newly infected cells, contributing to integration site preference [61].

Within Pol, we observed that the reverse transcriptase (RT) active center (YVDD), the RNase H residues (DSF), and the integrase (IN) zinc binding motif (BM) with residues HHCC were completely conserved (Figure S3). The IN active center (AC) was mostly conserved with residues DDE, except for SFVpan_V909-03F_MK241969, which has residues NDE. The RT-IN cleavage site (CS) YVVN/XNXX was partially conserved, with SFVpne_500057 and macaque SFV having YVVH/XNXX and SFVtfr_083616 having YVMN/XVXX.

For Env, the WXXW motif in the N-terminal cytoplasmic domain of the leader peptide subunit, important for capsid interaction, is evolutionarily conserved (Figure S4) [62]. The fusion peptide, membrane-spanning domain (MSD), and endoplasmic reticulum retrieval signal are relatively conserved. The optimal furin cleavage sites (RX[K/R]R) at the surface protein and transmembrane junction are conserved, but at the leader peptide and surface protein location, the residues are RXXR.

The transcriptional transactivator (Tas) protein of SFVs contains a bipartite nuclear localization signal (NLS) that directs Tas into the nucleus of cells, where it acts upon promoter sequences in the U3 region of the LTR to initiate transcription of the *gag*, *pol*, and *env* genes and in the internal promoter in *env* to facilitate transcription of the regulatory genes *tas* and *bet* [63]. Bioinformatic analysis identified the potential NLS for each SFV in the Tas alignment (Figure S5) and confirmed the high genetic heterogeneity in the NLS in Tas. Sequence analysis showed that the NLS of SFVpne_500057 shared more identity with those from *Macaca* SFV (64.7–76.5%) than those from African monkey SFV, including SFVcgu_910916 (35.3–41.2%). Deletions in *tas* have been reported for SFV tissue culture isolates [42,43,45,46]. Of the three new colobine SFV genomes, only SFVpne_500057 contained an in-frame Δtas variant of 294 bp in length in the assembled NGS genome (Figure S5). The position of Δtas occurs at the exact locations of the splice donor and acceptor junctions for generation of the *bet* coding region, such that Tas transcription is eliminated and only the Bet reading frame is preserved. We confirmed that the Δtas and intact *tas* genes were present in both the PBL tissue culture supernatants and PBLs from Pne_500057 with nested PCR from two collection dates (23 May 2000 and 7 November 2000). These results suggest the Δtas variant was likely selected in vitro and represented the majority variant in tissue culture.

While Bet has been shown to downregulate viral transcription, little is known about the regulatory motifs within Bet that are responsible for this functionality (Figure S6) [43,64]. One study has shown that the K/RGD motif in the C-terminus of Bet may be involved in binding integrins, which is known for its role in virus entry and infection [65]. Inspection of the Tas alignment identified a conserved XGD motif in SFVpne_500057, the macaque SFVs, and one baboon SFV (SFVpan_V909-03F), but only the D residue was present in the remaining monkey SFV Tas proteins (Figure S5). While one study reported the presence of an NLS in the C-terminus of a Tas protein from a human infected with a chimpanzee SFV, we did not find an NLS in any of the Asian or African monkey SFVs using the tool PSORTII [64].

**Distribution and phylogenetic characterization of novel colobine SFVs in wild and captive NHPs.** We developed and applied a new generic PCR assay for the detection of IN sequences by using the new SFV genomes obtained in our study. By using 10-fold titrations of nucleic acids recovered from PBL tissue culture supernatants of Cgu_910916, Pne_500057, and Tfr_083616, we found the new PCR assay was very sensitive and could detect 0.4 copies/reaction, 1.4 copies/reaction, and 0.6 copies/reaction, respectively. Upon testing of PBL DNA from 75 animals across seven Colobinae genera, of which 44% (33/75) were seropositive, we detected SFV sequences in 90.1% (30/33) of the animals (Table 4). We detected SFV sequences for the first time in two additional *Trachypithecus* species (*T. cristatus cristatus* and *T. obscurus*) by using the new PCR test. In contrast, only 27.3% (9/33) of the seropositive animals were confirmed with SFV infection by using the original PCR assay. The difference in proportions of SFV PCR-positive animals for each test was significant (*p* < 0.00001) using a two-tailed Z score test. None of the seronegative monkeys tested positive by using both the new and original SFV assays, demonstrating the high specificity of both PCR tests.

**Figure 2:**
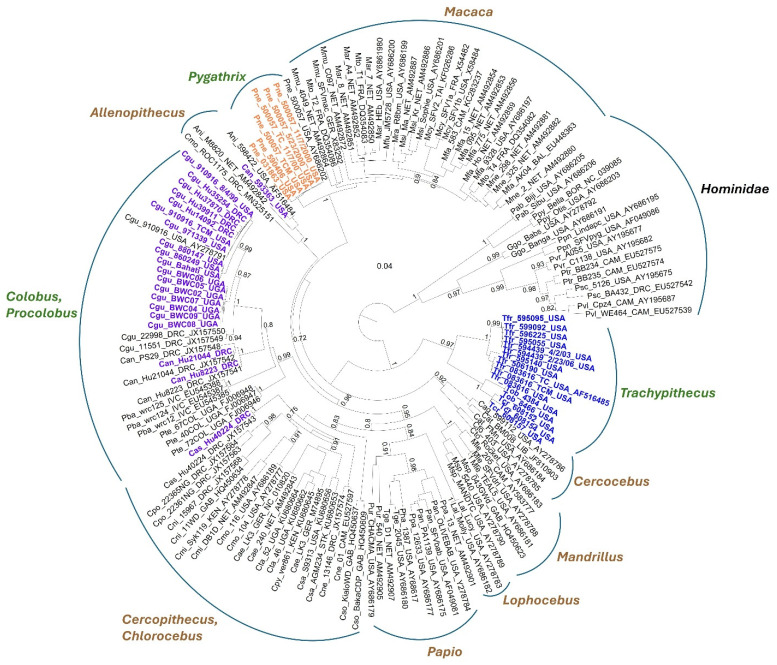
Maximum likelihood evolutionary relationships of new Colobinae simian foamy virus (SFV) integrase (IN) sequences to those from other Old-World primates show divergent evolutionary trajectories for Colobinae SFVs. The placement of the new *Colobus* species, *Pygathrix nemaeus nemaeus*, and *Trachypithecus* species IN sequences is shown in purple, orange, and blue text in the tree, respectively. The Colobinae clades have green text for the genus names; the Cercopithicinae clades have brown text for the genus names; the Hominidae clade is shown with black text. The sequence names are provided in the format: the first three letters are the three-letter genus and species code following the International Community on Taxonomy of Viruses guidelines, followed by the source name for the sequence, the three-letter country code, and the GenBank accession number when available. Hu in the source name indicates the sequence is from an infected human; TC indicates the sequence is from the tissue culture isolate; _TCM indicates that sequences are from the current study. Can, *Colobus angolensis*; Cgu, *Colobus guereza guereza*; Tfr, *Trachypithecus francoisi*; Tob, *Trachypithecus obscurus*; Tcr, *Trachypithecus cristatus cristatus*; Pne, *Pygathrix nemaeus nemaeus*; Pba, *Procolobus badius*; Pte, *Procolobus rufomitratus tephrosceles*; Ani, *Allenopithecus nigroviridis*; Mmu, *Macaca mulatta*; Mto, *Macaca tokeana*; Mar, *Macaca arctoides*; Mfu, *Macaca fuscata*; Mcy, *Macaca cyclopis*; Mfa, *Macaca fasicularis*; Mne, *Macaca nemistrina*; Mra, *Macaca radiata*; Msi, *Macaca silensis*; Pab, *Pongo abelii*; Ppy, *Pongo pygmaeus*; Ggo, *Gorilla gorilla*; Ppn, *Pan paniscus*; Pvr, *Pan troglodytes verus*; Ptr, *Pan t. troglodytes*; Psc, *P. t. schweinfurthii*; Pvl, *P. t. vellerosus*; Cag, *Cercocebus agilis*; Cat, *Cercocebus atys*; Cto, *Cercocebus torquatus*; Mle, *Mandrillus leucophaeus*; Msp, *Mandrillus sphinx*; Lal, *Lophocebus albigena*; Ppa, *Papio papio*; Pan, *Papio anubis*; Pha, *Papio hamadryas*; Pur, *Papio ursinus*; Tge, *Theropithecus gelada*; Cas, *Cercopithecus ascanius*; Cpo, *Cercopithecus pogonias*; Cni, *Cercopithecus nictitans*; Cmi, *Cercopithecus mitis*; Cmo, *Cercopithecus mona*; Cne, *Cercopithecus neglectus*; Cso, *Cercopithecus solatus*; Cae, *Chlorocebus aethiops*; Cta, *Chlorocebus tantalus*; Cpy, *Chlorocebus pygerythrus*; Cas, *Chlorocebus sabaeus*. DRC, Democratic Republic of Congo; BAL, Bali; BOR, Borneo; CAM, Cameroon; FRA, France; GAB, Gabon; GER, Germany; IVC, Ivory Coast; KEN, Kenya; LIB, Liberia; NET, Netherlands; STK, Saint Kitts and Nevis; TAI, Taiwan; UGA, Uganda; USA, United States of America. Shimodaira-Hasegawa support values ≥ 0.7 are provided at branch nodes. The scale bar indicates nucleotide substitutions per site.

Phylogenetic analysis revealed that, like the *gag-pol-env* concatemers from the three new SFV genomes, all *Trachypithecus* IN sequences clustered together basal to Cercopithecinae SFVs (Figure 2). Within the *Trachypithecus* clade, the SFV IN sequences from *T. cristatus cristatus* (Tcr) and *T. obscurus* (Tob) formed a sister clade to *T. francoisi* (Tfr) with high support (SH = 0.97), indicating cospeciation. The African colobine SFV (Cgu, Can, Cas) formed a single clade basal to the African Cercopithecinae SFVs, consistent with the *gag-pol-env* concatemer analysis, and included species-specific subclades for colobus and procolobus (Pba, Pte) SFVs. All *P. nemaeus* SFVs formed a clade basal to *Macaca* SFVs with strong support (SH = 1), rather than clustering within *Macaca* SFVs in the *gag-pol-env* analysis. Notably, one *C. angolensis* (Can_593363) IN sequence clustered with captive *Allenopithecus* SFVs and an IN sequence from a wild-borne *Cercopithecus mona* monkey from DRC. Can_593363 was housed with *Allenopithecus* monkeys in a zoological garden, and in the wild, the distributions of *C. mona* and *Allenopithecus* monkeys overlap. Both scenarios likely explain the observed cross-species SFV infections and phylogenetic clustering of their SFV sequences.

*Detection of Colobinae SFV in persons exposed to NHPs in DRC.* Next, we used the new SFV PCR assay to re-test buffy coat DNA from participants in DRC who were SFV WB seropositive or seroindeterminate in a previous study (Table 5) [13]. Persons with seroindeterminate WB results showed reactivity to only a single Gag protein. In the former study, we only detected SFV sequences in three women, of which two originated from *C. angolensis* and one from *C. ascanius*, by using the original PCR test [13]. With the new PCR assay, we detected SFV sequences in an additional four people (three men, one woman) who were all infected with *C. guereza* SFV as determined by phylogenetic analysis (Table 5, Figure 2). Two of these four people had seropositive results, while the other two were seroindeterminate (Table 5). We also confirmed SFV infection and the simian origin of the SFV in the first three women identified in the original study (Table 5, Figure 2). Five of these seven SFV-infected people reported various simian exposures, while the other two persons, one man and one woman, did not report specific primate exposures but frequently visited the forests surrounding their villages (Table 5). Nonetheless, we previously demonstrated that people with frequent forest visits were at increased risk for SFV infection, possibly from contact with urine or feces from infected animals [13]. The natural habitats of *C. angolensis, C. guereza*, and *C. ascanius* include the forests of DRC, providing epidemiological support for our findings.

## 4. Discussion

We obtained and characterized three new SFV genomes from two colobine monkeys (*Trachypithecus francoisi* and *Pygathrix nemaeus*) naturally found in Asia and one from Africa (*Colobus guereza*) by using a detailed sequence analysis of PBL tissue culture isolates. Previously, the only complete SFV genomes from Asian monkeys were from a single genus, *Macaca*, severely limiting conclusions about SFV evolution and biology and the design of diagnostic assays. Using the new genomes, we improved our diagnostic assays and confirmed infection in additional people from DRC with NHP and forest exposures. The new colobine SFV genomes also enabled a better understanding of the evolutionary histories of these ancient viruses, which showed phylogenies not supporting the SFV host co-evolution model.

Overall, the three new SFV genomes were identical in structure to those of other SFVs and included relatively conserved structural, enzymatic, and auxiliary genes flanked by LTRs. Although the SFVtfr_083616 and SFVcgu_910916 genomes were intact, consisting of all five major coding regions, an in-frame Δtas mutation was present in the SFVpne_500057 genome that effectively eliminates the *tas* open reading frame with that of *bet* without the need for alternative splicing. This mutation has been reported to cause increased Bet production, favoring viral latency by downregulation of viral replication [46]. We confirmed the presence of this mutation and a wild-type SFV in Pne_500057 PBLs and a PBL tissue culture isolate. While some have suggested the Δtas mutation may be an adaptation to tissue culture, we and others have reported this mutation in both NHP and human ex vivo blood specimens, suggesting the Δtas also likely contributes to SFV latency in vivo by upregulation of Bet in the absence of Tas [42,43,45,46]. Further, viral latency, combined with immunological suppression by neutralizing antibodies, interferon, and host restriction by apolipoprotein B-editing catalytic polypeptide-like (APOBEC) deaminases, likely explains the low viral loads in zoonotically SFV-infected individuals and the absence of person-to-person transmission [66,67,68,69].

Increased risks of pathogen acquisition from colobine monkeys in Asia and Africa are caused by hunting and keeping them as pets and activities related to habitat loss or fragmentation caused by logging and agricultural expansion [13,39,70,71,72,73]. Occupational exposures to colobine monkeys can also occur at zoological gardens, where they are frequently kept in collections for their distinctive physical features and to promote primate conservation and education. Previously, we demonstrated that women in the DRC with NHP exposure were at increased risk for SFV infection, including two confirmed by molecular analyses to be infected with the SFV originating from *Colobus angolensis* and one with infection from *Cercopithecus ascanius* [13]. In the current study, we used a new generic SFV PCR assay developed with the new Colobinae genomes to re-test buffy coat DNA samples from 22 persons in DRC with primate exposure who were seroreactive for SFV, of whom 19 were originally PCR negative [13]. We demonstrated the new PCR assay to be significantly more sensitive than the original assay used for detecting Colobinae SFV. Our application of the new assay identified SFV sequences in four more people with infection originating from *Colobus guereza*, expanding the known diversity of SFV variants infecting humans. We also amplified longer *pol* sequences from the three SFV-infected people reported in our previous study, confirming their infections with *Colobus* and *Cercopithecus* SFVs. The habitat of *C. guereza* includes the forests of DRC, where our study was located, making the detection of this SFV strain in the four people who frequented the forests surrounding their villages plausible. Three of these four SFV-infected people reported direct NHP exposures, including butchering and eating NHPs. The fourth person did not report NHP exposure, but we have shown that simply entering forest environments places people at higher risk of SFV infection, likely from contact with body fluids of NHPs present in the forests [13]. Our inability to detect SFV sequences in the remaining 15 SFV seroreactive persons may indicate infection with other divergent SFVs not detected by our improved assay or low proviral loads, which are common with SFV infection [13,74].

Our detailed sequence analyses indicate SFV evolution has not strictly mirrored host phylogeny but rather has a more complex and dynamic history punctuated by host-switching events. Bayesian inference of *gag-pol-env* phylogenies showed that each new SFV was distinct from the others and previously reported SFV genomes from monkeys and apes. Surprisingly, all three colobinae SFV sequences demonstrated diverse evolutionary trajectories without forming a monophyletic clade, which would have been expected based on the prevailing SFV–host co-evolutionary dogma [12,16,40]. We confirmed these Colobinae SFV phylogenetic relationships by analysis of *pol* sequences from multiple colobine species with evidence of species-specific clades for Trachypithecus and African colobines. One exception was the placement of *P. nemeaus* SFV basal to *Macaca* SFV in the *pol* tree, compared with its position within the *Macaca* SFV clade in the *gag-pol-env* concatemer analysis, which was likely resolved by the inclusion of additional *P. nemeaus* SFV *pol* sequences. Our phylogenetic and TMRCA results do not mirror the evolutionary history of the colobine monkeys based on analysis of mitochondrial genomes, which proposes an origin in Africa 18–16 mya, with the colobus/procolobus divergence occurring in Africa around 9–7.5 mya [75]. These mitochondrial TMRCA estimates are consistent with the colobinae fossil record [56]. The Asian colobine ancestor was then inferred to have diverged from the African colobines 12–10 mya, followed by populating Eurasia [75]. About 2 mya later in Asia, the colobine monkeys diverged into the *Semnopithecus*, *Trachypithecus/Presbytis*, and the odd-nosed monkeys (*Simias, Nasalis, Pygathrix, Rhinopithecus*) [76]. In contrast to the colobine host evolutionary histories, the *Trachypithecus* SFV had the oldest estimated TMRCA (24–15 mya), suggesting that colobine SFVs may have originated in Asia and that African colobine SFVs represent a more recent divergence, potentially arising 8–14 mya from African Cercopithecinae.

Several factors may explain the unexpectedly deep divergences within colobine SFVs. One possibility is that the *Trachypithecus* SFVs reflect long-term virus–host co-divergence within Asian colobines, whereas the African colobine SFVs may have been acquired later through an ancient host switch from African cercopithecines. Alternatively, the deep divergence of the *Trachypithecus* SFV lineage may represent the retention of an older SFV lineage that arose early in cercopithecid evolution, with subsequent lineage loss or replacement in other Asian colobines. Under this scenario, the contemporary SFV distribution in colobines could reflect a combination of deep-time divergence, differential lineage survival, and limited cross-species transmission early in catarrhine dispersal between Africa and Eurasia, although the fossil record provides only indirect support for this broader geographic connectivity. This pattern is reminiscent of the phylogeny of squirrel monkey SFVs (*Saimiri sciureus*), which are basal to other platyrrhine SFVs rather than clustering with other Cebidae SFVs, indicating the persistence of deep viral lineages and/or ancient host switches [16,59,77]. Similarly, the basal position of *Pygathrix* SFVs relative to macaque SFVs suggests a shared ancestral SFV lineage or an early spillover event between colobines and macaques. The overlapping distributions of *P. nemaeus* and macaques in Southeast Asia raise the possibility that the SFV infection detected in Pne_500057 resulted from natural cross-species transmission rather than during captivity. If additional SFVs from other Asian colobines cluster with the *Pygathrix* SFV lineage, this would support the hypothesis that Old-World SFVs primarily group by host geography (Africa vs. Asia) rather than by strict host phylogeny, with the *Trachypithecus* SFVs representing an exception due to their unusually deep divergence. Together, these findings reveal a more complex and dynamic evolutionary history for SFVs in colobines than previously appreciated, including the potential for ancient host switches, deep viral lineage preservation, and hidden diversity in under-sampled Asian primates. This underscores the need to include diverse, under-sampled host species, such as colobines, in studies of SFV phylogenetics. Further sampling of Asian colobine SFVs, ideally from wild-caught individuals, would be essential to distinguish among these hypotheses and clarify the evolutionary history and host associations of SFVs in Old-World monkeys.

Our study has limitations. Interpretation of SFV phylogenies requires caution because divergence-time estimates for both viruses and hosts carry large credible intervals, and SFVs may experience lineage extinction, replacement, or unrecognized recombination [16,59,76,77,78,79]. Fossil evidence for early catarrhine dispersal across Africa and Eurasia is sparse and does not yet establish direct sympatry between African and Asian colobines [35,74,75]. Thus, alternative scenarios for the deep divergence of the *Trachypithecus* SFV lineage, including long-term co-divergence, ancient host switches, or retention of relict viral lineages, remain plausible but not definitively resolved. Broader geographic and taxonomic sampling of wild colobines, especially from underrepresented Asian lineages, will be crucial for distinguishing among these hypotheses and for refining the evolutionary history of SFVs in Old-World monkeys. Finally, the human samples tested were restricted to individuals from a specific region in the DRC, potentially missing SFV variants present in humans with different exposure histories. Despite improved PCR assays, SFV sequences were not detected in all seroreactive individuals, possibly due to low proviral loads or the presence of highly divergent SFV strains not targeted by the new assay. While our study identified new human infections, clinical and detailed contact information was not available to investigate clinical outcomes, transmission routes, or broader public health implications of SFV infection in humans.

In summary, we sequenced and analyzed three new SFV genomes from diverse Asian and African colobine monkeys, expanding the SFV genomic database. These new genomes enabled the development of improved diagnostic assays, which detected additional SFV infections in humans exposed to NHPs in the DRC. Genomic and phylogenetic analyses revealed that Colobinae SFVs have conserved genetic structures but follow complex evolutionary paths, including host switching, rather than strict co-evolution with their primate hosts. Our study highlights the need for broader SFV sampling to better understand viral evolution and zoonotic risks.

## Figures and Tables

**Table 1:** Comparison of the three new Colobinae simian foamy virus (SFV) genomes and gene nucleotide lengths with those from representative Cercopithecinae SFVs ^1^.

Virus	Genome	LTR	*gag*	*pol*	*env*	*tas*	*bet*
**SFVtfr_083616**	12,645	1492	1893	3489	2967	903	1446
**SFVpne_500057**	12,944	1622	1923	3450	2970	927	1464
**SFVcgu_910916**	12,644	1499	1878	3435	2955	912	1482
**SFVpan_V909 (MK241969)**	13,393	1856	1902	3432	2949	906	1485
**SFVcae_LK3 (M74895)**	13,111	1708	1944	3432	2949	894	1407
**SFVmcy_FV21 (MN585198)**	12,972	1621	1932	3450	2970	927	1464

^1.^ LTR, long terminal repeat; *gag*/Gag, group-specific antigen; *pol*/Pol, polymerase; *env*/Env, envelope; *tas*/Tas, transactivator gene; *bet*/Bet, between *env* and *tas* genes. tfr, *Trachypithecus francoisi*; pne, *Pygathrix nemaeus nemaeus*; cgu, *Colobus guereza*; pan; *Papio anubis*; cae, *Chlorocebus aethiops*; mcy, M*acaca cyclopis*. Specimens used in the current study are indicated with IDs in purple text.

**Table 2:** Percent nucleotide and amino acid identities of new Colobinae simian foamy viruses (SFVs) with related Old-World monkey SFVs ^1^.

SFV IDs	SFVtfr_083616	SFVpne_500057	SFVcgu_910916	SFVpan_V909 (MK241969)	SFVcae_LK3 (M74895)	SFVmcy_FV21 (MN585198)
**SFVtfr_083616**		Gag: 50.0 Pol: 81.3 Env: 70.1 Tas: 39.5 Bet: 43.1	Gag: 48.4 Pol: 79.5 Env: 66.1 Tas: 40.8 Bet: 41.7	Gag: 47.1 Pol: 80.7 Env: 67.6 Tas: 37.1 Bet: 39.2	Gag: 45.9 Pol: 80.0 Env: 66.1 Tas: 41.3 Bet: 43.3	Gag: 49.0 Pol: 80.8 Env: 70.0 Tas: 40.5 Bet: 42.3
**SFVpne_500057**	LTR: 43.7 *gag*: 53.7 *pol*: 76.5 *env*: 71.1 *tas*: 53.7 *bet*: 53.2 genome: 61.2		Gag: 58.0 Pol: 85.1 Env: 74.3 Tas: 52.8 Bet: 50.3	Gag: 61.8 Pol: 86.2 Env: 76.6 Tas: 48.0 Bet: 50.7	Gag: 62.7 Pol: 85.3 Env: 73.6 Tas: 53.5 Bet: 54.0	Gag: 85.5 Pol: 94.6 Env: 91.7 Tas: 80.2 Bet: 78.7
**SFVcgu_910916**	LTR: 49.3 *gag*: 54.2 *pol*: 75.8 *env*: 68.6 *tas*: 57.7 *bet*: 55.3 genome: 61.2	LTR: 54.3 *gag*: 61.4 *pol*: 80.2 *env*: 74.0 *tas*: 63.8 *bet*: 59.4 genome: 66.6		Gag: 57.7 Pol: 83.7 Env: 81.3 Tas: 58.3 Bet: 50.5	Gag: 67.6 Pol: 83.8 Env: 77.5 Tas: 55.7 Bet: 51.7	Gag: 57.7 Pol: 85.4 Env: 74.2 Tas: 53.1 Bet: 49.7
**SFVpan_V909 (MK241969)**	LTR: 38.4 *gag*: 56.7 *pol*: 76.7 *env*: 68.2 *tas*: 55.3 *bet*: 52.7 genome: 58.4	LTR: 52.3 *gag*: 63.5 *pol*: 81.1 *env*: 73.5 *tas*: 62.6 *bet*: 59.2 genome: 66.3	LTR: 48.4 *gag*: 66.2 *pol*: 80.8 *env*: 77.7 *tas*: 68.3 *bet*: 61.2 genome: 67.2		Gag: 71.2 Pol: 86.6 Env: 80.6 Tas: 56.8 Bet: 59.7	Gag: 60.5 Pol: 86.9 Env: 75.7 Tas: 49.3 Bet: 52.1
**SFVcae_LK3 (M74895)**	LTR: 43.3 *gag*: 54.5 *pol*: 76.8 *env*: 68.7 *tas*: 54.5 *bet*: 54.4 genome: 60.0	LTR: 55.8 *gag*: 63.4 *pol*: 80.7 *env*: 72.1 *tas*: 64.3 *bet*: 62.3 genome: 67.0	LTR: 55.7 *gag*: 66.2 *pol*: 81.1 *env*: 75.7 *tas*: 66.5 *bet*: 61.8 genome: 70.1	LTR: 55.7 *gag*: 68.1 *pol*: 82.0 *env*: 77.5 *tas*: 68.2 *bet*: 64.0 genome: 70.0		Gag: 62.6 Pol: 85.6 Env: 73.1 Tas: 54.2 Bet: 53.5
**SFVmcy_FV21 (MN585198)**	LTR: 44.6 *gag*: 54.7 *pol*: 76.7 *env*: 70.2 *tas*: 53.4 *bet*: 52.9 genome: 61.2	LTR: 81.3 *gag:* 81.7 *pol:* 88.7 *env:* 85.4 *tas:* 83.3 *bet:* 80.1 genome: 84.3	LTR: 55.3 *gag*: 62.8 *pol*: 80.7 *env*: 73.0 *tas*: 63.7 *bet*: 59.5 genome: 67.0	LTR: 52.8 *gag*: 63.3 *pol*: 81.5 *env*: 73.7 *tas*: 63.2 *bet*: 60.2 genome: 66.9	LTR: 56.4 *gag*: 64.1 *pol*: 81.4 *env*: 72.6 *tas*: 64.5 *bet*: 60.6 genome: 67.3	

^1.^ Nucleotide identities are the light blue cells in the lower matrix. Amino acid identities are the light green cells in the upper matrix. Gray cells indicate no comparisons of self vs. self. Specimens used in the current study are indicated with IDs in purple text. LTR, long terminal repeat; *gag*/Gag, group-specific antigen; *pol*/Pol, polymerase; *env*/Env, envelope; *tas*/Tas, transactivator gene; *bet*/Bet, between *env* and *tas* genes. tfr, *Trachypithecus francoisi*; pne, *Pygathrix nemaeus nemaeus*; cgu, *Colobus guereza*; pan; *Papio anubis*; cae, *Chlorocebus aethiops*; mcy, M*acaca cyclopis*.

**Table 3:** Time to most recent common ancestor (TMRCA) estimates in millions of years ago and nucleotide substitution rates for foamy viruses (FVs) ^1^.

FV ^2^, Host, or Analysis Parameter	*FV Gag-Pol-Env* ^3^	95% HPD	Host ^4^	95% HPD
SFVtfr_083616 (Asian Colobinae/Cercopithecinae + SFVcgu split)	19.12	14.60–24.29	11.56	7.44–16.68
SFVcgu_910916 (African Colobinae/Cercopithecinae split)	10.78	7.68–13.87	11.56	7.44–16.68
Crown Macaca	3.80	3.19–4.38	3.47	2.81–4.14
SFVmmu/SFVpne_500057 split	2.07	1.28–2.90	N/A ^5^	N/A
Crown Catarrhini (Hominoidae + Cercopithecoidae)	23.15	18.63–27.82	30.15	25.19–35.11
Crown Cercopithecidae (Colobinae + Cercopithecinae)	19.12	14.60–24.29	10.75	6.54–14.96
Crown Hominoidae	19.99	15.86–24.23	19.32	13.44–25.20
Crown Primates	62.54	40.04–80.84	61.02	N/A
Crown Atelidae	14.14	8.14–20.52	23.93	13.37–34.49
Non-simian FV (horse + cow/cat split)	91.32	84.37–97.76	80.60	63.00–115.40
BFV/EFV split (cow/horse split)	77.39	73.56–81.23	78.00	74.10–81.92
Root FV placental mammals (Crown Boreoutheria)	93.11	97.32–98.88	96.00	89.92–102.10
Mean rate ^7^	1.26 × 10^8^	1.12 × 10^8^–1.36 × 10^8^	1.16 × 10^8^ ^6^	0.81–1.51 × 10^8^
ucld.stdev ^8^	0.694	0.556–0.848	N/A	N/A
α-parameter (Γ-distribution) ^9^	0.974	0.906–1.046	N/A	N/A
ESS ^10^	>290	N/A	N/A	N/A

^1.^ Median TMRCAs; 95%-high posterior density values are shown in parentheses. ^2.^ SFVhpi, SFV from *Hylobates pileatus*; OWM, Old-World monkeys; SFVpve, SFV from *Pan troglodytes verus*; SFVpsc, SFV from *Pan troglodytes schweinfurthii*; SFVggo, SFV from *Gorilla gorilla.* Non-simian foamy viruses include bovine foamy virus (BFV), equine foamy virus (EFV), and feline foamy virus (FFV). ^3.^ 12 cdp, 1st and 2nd codon positions. The 12 cdp length is 4.942 kb versus 7.412 kb for the complete coding concatemer. ^4.^ Primate and non-primate divergence dates from de Vries et al. (primate fossil dates) and dos Reis et al. (mammalian nuclear genomes and mitochondrial genomes) used as calibration points as normal or exponential (only crown primates) priors [56,57]. ^5.^ N/A, not available. ^6.^ Primate mitochondrial nucleotide substitution rates/site/year from Switzer et al. and Ho et al. [12,60]. ^7.^ Mean evolutionary rate in nucleotide substitutions/site/year. ^8.^ Standard deviation (stdev) of the uncorrelated lognormal (ucld) relaxed clock which is the BEAST analysis parameter indicating the amount of variation in the substitution rate across branches. Values close to zero indicate little substitution rate variation and the presence of a molecular clock, whereas values > 1 indicate substantial rate heterogeneity amongst lineages. ^9.^ Shape parameter of the gamma (Γ) distribution of rate heterogeneity among sites. ^10.^ ESS, effective sampling size values for all BEAST parameters.

**Table 4:** Colobinae specimens and simian foamy virus (SFV) infection status using serological and molecular analysis.

Common Name	Scientific Name	Three Letter Simian Code	Natural Geographical Habitat	Captive/Wild	No. Animals	SFV Serology Positives ^1^ (%)	SFV IN PCR Positives (OG ^2^) (%)	SFV IN PCR Positives (New ^3^) (%)
Red-shanked douc langur	*Pygathrix nemaeus nemaeus*	pne	Indochina	Captive	5	3 (60.0)	0/3 (0.0)	3/3 (100.0)
Silver leaf monkey	*Trachypithecus cristatus cristatus*	tcr	Borneo, Sumatra, Java	Captive	8	4 (50.0)	0/4 (0.0)	3/4 (75.0)
Francois langur	*Trachypithecus francoisi*	tfr	Southwest China, northern Vietnam	Captive	18	11 (61.1)	0/11 (0.0)	9/11 (81.8)
Dusky leaf monkey	*Trachypithecus obscurus*	tob	Malaysia, Myanmar, Thailand	Captive	2	2 (100.0)	0/2 (0)	2/2 (100.0)
Black and white colobus	*Colobus guereza guereza*	cgu	Equatorial Africa	Captive	11	5 (45.5)	5/5 (100.0)	5/5 (100.0)
Black and white colobus	*Colobus guereza guereza*	cgu	Uganda	Wild	9	7 (77.8)	4/7 (57.1)	7/7 (100.0)
Angolan colobus	*Colobus angolensis*	can	Congo basin	Captive	22	1 (4.5)	0/1 (0)	1/1 (100.0)
***Totals***					75	33 (44.0)	9/33 (27.3)	30/33 (90.1)

^1.^ SFV serology was done by using a combination of EIA and western blot testing using SFV antigens. ^2.^ OG, original generic SFV integrase (IN) PCR assay. ^3.^ New testing was done by using new generic SFV IN PCR modified using the new Colobinae SFV genomes.

**Table 5:** Identification of SFV infection in persons exposed to nonhuman primates in rural DRC.

ID	Sex, Age	Village	Forest Frequency	NHP Exposure ^1^	SFV WB	OG SFV IN PCR ^2^	New SFV IN PCR ^3^
6753	F, 12	Tokondo	Never	B, PE, E	Positive	Negative	Negative
8223	F, 23	Tokondo	Everyday	B, PE, E	Positive	*Col. angolensis*	*Col. angolensis*
14092	M, 13	Loseke	2–4 times/month	PDE, B	Positive	Negative	*Col. guereza*
21044	F, 57	Asenge	Everyday	PE, E	Positive	*Col. angolensis*	*Col. angolensis*
22492	F, 62	Olombo Munene	>4 times/month	B, PE, E, S, BN	Positive	N/A	N/A
23542	M, 32	Asenge	2–4 times/month	N/A	Positive	Negative	Negative
26806	F, 50	Olombo Munene	Once a month	PE, E	Indeterminate	Negative	Negative
30015	F, 62	Asenge	Everyday	B, PE, E	Positive	Negative	Negative
32863	F, 36	Asenge	Everyday	B, PE, E	Positive	Negative	Negative
33740	F, 13	Asenge	>4 times/month	B, PE, E	Positive	Negative	Negative
35222	F, 18	CERS Kole Yango	Everyday	None reported	Positive	Negative	Negative
35254	F, 30	Asenge	>4 times/month	E	Indeterminate	Negative	*Col. guereza*
36036	M, 34	CERS Kole Yango	N/A	None reported	Indeterminate	Negative	Negative
37870	M, 35	Djombe	>4 times/month	None reported	Indeterminate	Negative	*Col. guereza*
38662	F, 21	CERS Kole Yango	>4 times/month	B, PE, E	Positive	Negative	Negative
39443	F, 11	CERS Kole Yango	Never	E	Positive	Negative	Negative
39911	M, 59	Djombe	Everyday	B, PE, E	Positive	Negative	*Col. guereza*
40165	M, 45	CERS Kole Yango	N/A	None reported	Indeterminate	Negative	Negative
40224	F, 50	Djombe	>4 times/month	None reported	Positive	*Cer. ascanius*	*Cer. ascanius*
41274	F, 25	CERS Kole Yango	Everyday	PE, E	Positive	Negative	Negative
41812	F, 78	CERS Kole Yango	Once a month	None reported	Indeterminate	Negative	Negative
42652	M, 29	Kole Yango	>4 times/month	E	Positive	Negative	Negative

^1.^ NHP, nonhuman primate; PDE, pick up dead monkey to eat; B, butcher monkey; PE, prepare monkey to eat; E, eat monkey; S, scratched by monkey; BN, bitten by monkey. ^2.^ OG, original integrase (IN) PCR assay; when PCR is positive, most likely monkey origin of infection is provided based on phylogenetic analysis of available sequences. N/A, specimens or data not available. ^3.^ New generic SFV IN PCR modified by using the new colobinae SFV genomes. Information for the four persons testing positive with the new PCR assay are indicated in purple text.

## Data Availability

The complete genomes of SFVcgu_910916, SFVpne_500057, and SFVtfr_083616 were assigned Genbank accession numbers PP966955, PP966956, and PP966957, respectively. The human and monkey SFV pol sequences have accession numbers PV939864-PV939903.

## Supplementary Materials

The following supporting information can be downloaded at https://www.mdpi.com/article/10.3390/v18030320/s1. Figure S1. Nucleotide and amino acid sequence alignment of the SFVcgu_910916, SFVpne_500057, and SFVtfr_083616 genomes. Figure S2. Group-specific antigen (Gag) amino acid alignments of SFVcgu_910916, SFVpne_500057, and SFVtfr_083616 with five Asian and five African SFV. Figure S3. Polymerase (Pol) amino acid alignments of SFVcgu_910916, SFVpne_500057, and SFVtfr_083616 with five Asian and five African SFV. Figure S4. Envelope (Env) amino acid alignments of SFVcgu_910916, SFVpne_500057, and SFVtfr_083616 with five Asian and five African SFV. Figure S5. Transactivator (Tas) amino acid alignments of SFVcgu_910916, SFVpne_500057, and SFVtfr_083616 with five Asian and five African SFV. Figure S6. Between envelope and transcriptional transactivator (Bet) amino acid alignments of SFVcgu_910916, SFVpne_500057, and SFVtfr_083616 with five Asian and five African SFV. Table S1: Primate and non-primate fossil calibrations for Bayesian inference of simian foamy virus time to most recent common ancestor.

## References

[1] Calattini S., Nerrienet E., Mauclere P., Georges-Courbot M.C., Saib A., Gessain A. (2004). Natural simian foamy virus infection in wild-caught gorillas, mandrills and drills from Cameroon and Gabon. J. Gen. Virol..

[2] Calattini S., Wanert F., Thierry B., Schmitt C., Bassot S., Saib A., Herrenschmidt N., Gessain A. (2006). Modes of transmission and genetic diversity of foamy viruses in a Macaca tonkeana colony. Retrovirology.

[3] Goldberg T.L., Sintasath D.M., Chapman C.A., Cameron K.M., Karesh W.B., Tang S., Wolfe N.D., Rwego I.B., Ting N., Switzer W.M. (2009). Coinfection of Ugandan red colobus (*Procolobus* [*Piliocolobus*] *rufomitratus tephrosceles*) with novel, divergent delta-, lenti-, and spumaretroviruses. J. Virol..

[4] Jones-Engel L., Engel G.A., Heidrich J., Chalise M., Poudel N., Viscidi R., Barry P.A., Allan J.S., Grant R., Kyes R. (2006). Temple monkeys and health implications of commensalism, Kathmandu, Nepal. Emerg. Infect. Dis..

[5] Khan A.S., Bodem J., Buseyne F., Gessain A., Johnson W., Kuhn J.H., Kuzmak J., Lindemann D., Linial M.L., Lochelt M. (2018). Spumaretroviruses: Updated taxonomy and nomenclature. Virology.

[6] Leendertz S.A., Junglen S., Hedemann C., Goffe A., Calvignac S., Boesch C., Leendertz F.H. (2010). High prevalence, coinfection rate, and genetic diversity of retroviruses in wild red colobus monkeys (*Piliocolobus badius badius*) in Tai National Park, Cote d’Ivoire. J. Virol..

[7] Liu W., Worobey M., Li Y., Keele B.F., Bibollet-Ruche F., Guo Y., Goepfert P.A., Santiago M.L., Ndjango J.B., Neel C. (2008). Molecular ecology and natural history of simian foamy virus infection in wild-living chimpanzees. PLoS Pathog..

[8] Morozov V.A., Leendertz F.H., Junglen S., Boesch C., Pauli G., Ellerbrok H. (2009). Frequent foamy virus infection in free-living chimpanzees of the Tai National Park (Cote d’Ivoire). J. Gen. Virol..

[9] Mouinga-Ondeme A., Betsem E., Caron M., Makuwa M., Salle B., Renault N., Saib A., Telfer P., Marx P., Gessain A. (2010). Two distinct variants of simian foamy virus in naturally infected mandrills (*Mandrillus sphinx*) and cross-species transmission to humans. Retrovirology.

[10] Santos A.F., Cavalcante L.T.F., Muniz C.P., Switzer W.M., Soares M.A. (2019). Simian Foamy Viruses in Central and South America: A New World of Discovery. Viruses.

[11] Switzer W., Ahuka-Mundeke S., Tang S., Shankar A., Wolfe N., Heneine W., Peeters M., Ayouba A., Mulembakani P., Rimoin A. (2011). Simian Foamy Virus (SFV) infection from multiple monkey species in women from the Democratic Republic of Congo. Retrovirology.

[12] Switzer W.M., Salemi M., Shanmugam V., Gao F., Cong M.E., Kuiken C., Bhullar V., Beer B.E., Vallet D., Gautier-Hion A. (2005). Ancient co-speciation of simian foamy viruses and primates. Nature.

[13] Switzer W.M., Tang S., Ahuka-Mundeke S., Shankar A., Hanson D.L., Zheng H., Ayouba A., Wolfe N.D., LeBreton M., Djoko C.F. (2012). Novel simian foamy virus infections from multiple monkey species in women from the Democratic Republic of Congo. Retrovirology.

[14] Murray S.M., Linial M.L. (2006). Foamy virus infection in primates. J. Med. Primatol..

[15] Mayer J., Blanco-Melo D., Coffin J.M., Gifford R.J., Johnson W.E., Lindemann D., Peeters M., Sato K., Stoye J., Tachedjian G. (2025). 2024 taxonomy update for the family Retroviridae. Arch. Virol..

[16] Katzourakis A., Aiewsakun P., Jia H., Wolfe N.D., LeBreton M., Yoder A.D., Switzer W.M. (2014). Discovery of prosimian and afrotherian foamy viruses and potential cross species transmissions amidst stable and ancient mammalian co-evolution. Retrovirology.

[17] Khan A.S. (2009). Simian foamy virus infection in humans: Prevalence and management. Expert Rev. Anti Infect. Ther..

[18] Switzer W.M., Heneine W., Liu D. (2011). Foamy virus infection of humans. Molecular Detection of Human Viral Pathogens.

[19] Pinto-Santini D.M., Stenbak C.R., Linial M.L. (2017). Foamy virus zoonotic infections. Retrovirology.

[20] Betsem E., Patricia T., Alain F., Gessain A. (2011). Frequent acquisition of simian foamy viruses from gorillas, chimpanzees and monkeys through severe bites in central African hunters with no evidence for intra-familial dissemination. Retrovirology.

[21] Betsem E., Rua R., Tortevoye P., Froment A., Gessain A. (2011). Frequent and recent human acquisition of simian foamy viruses through apes’ bites in central Africa. PLoS Pathog..

[22] Engel G.A., Small C.T., Soliven K., Feeroz M.M., Wang X., Kamrul Hasan M., Oh G., Rabiul Alam S.M., Craig K.L., Jackson D.L. (2013). Zoonotic simian foamy virus in Bangladesh reflects diverse patterns of transmission and co-infection. Emerg. Microbes Infect..

[23] Switzer W.M., Tang S., Zheng H., Shankar A., Sprinkle P.S., Sullivan V., Granade T.C., Heneine W. (2016). Dual Simian Foamy Virus/Human Immunodeficiency Virus Type 1 Infections in Persons from Cote d’Ivoire. PLoS ONE.

[24] Boneva R.S., Switzer W.M., Spira T.J., Bhullar V.B., Shanmugam V., Cong M.E., Lam L., Heneine W., Folks T.M., Chapman L.E. (2007). Clinical and virological characterization of persistent human infection with simian foamy viruses. AIDS Res. Hum. Retroviruses.

[25] Buseyne F., Betsem E., Montange T., Njouom R., Bilounga Ndongo C., Hermine O., Gessain A. (2018). Clinical Signs and Blood Test Results Among Humans Infected With Zoonotic Simian Foamy Virus: A Case-Control Study. J. Infect. Dis..

[26] Rudicell R.S., Holland Jones J., Wroblewski E.E., Learn G.H., Li Y., Robertson J.D., Greengrass E., Grossmann F., Kamenya S., Pintea L. (2010). Impact of simian immunodeficiency virus infection on chimpanzee population dynamics. PLoS Pathog..

[27] Allan J.S., Leland M., Broussard S., Mone J., Hubbard G. (2001). Simian T-cell lymphotropic Viruses (STLVs) and lymphomas in African nonhuman primates. Cancer Investig..

[28] Switzer W.M., Heneine W., Owen S.M., Jorgensen J.H., Pfaller M. (2015). Human T-Cell Lymphotropic Viruses. Manual of Clinical Microbiology.

[29] Locatelli S., Peeters M. (2012). Cross-species transmission of simian retroviruses: How and why they could lead to the emergence of new diseases in the human population. AIDS.

[30] Switzer W.M., Bhullar V., Shanmugam V., Cong M.E., Parekh B., Lerche N.W., Yee J.L., Ely J.J., Boneva R., Chapman L.E. (2004). Frequent simian foamy virus infection in persons occupationally exposed to nonhuman primates. J. Virol..

[31] Brooks J.I., Rud E.W., Pilon R.G., Smith J.M., Switzer W.M., Sandstrom P.A. (2002). Cross-species retroviral transmission from macaques to human beings. Lancet.

[32] Huang F., Wang H., Jing S., Zeng W. (2012). Simian foamy virus prevalence in Macaca mulatta and zookeepers. AIDS Res. Hum. Retroviruses.

[33] Hood S., Mitchell J.L., Sethi M., Almond N.M., Cutler K.L., Rose N.J. (2013). Horizontal acquisition and a broad biodistribution typify simian foamy virus infection in a cohort of Macaca fascicularis. Virol. J..

[34] Murphy H.W., Miller M., Ramer J., Travis D., Barbiers R., Wolfe N.D., Switzer W.M. (2006). Implications of simian retroviruses for captive primate population management and the occupational safety of primate handlers. J. Zoo Wildl. Med..

[35] Roos C., Zinner D., Kubatko L.S., Schwarz C., Yang M., Meyer D., Nash S.D., Xing J., Batzer M.A., Brameier M. (2011). Nuclear versus mitochondrial DNA: Evidence for hybridization in colobine monkeys. BMC Evol. Biol..

[36] Shao Y., Zhou L., Li F., Zhao L., Zhang B.L., Shao F., Chen J.W., Chen C.Y., Bi X., Zhuang X.L. (2023). Phylogenomic analyses provide insights into primate evolution. Science.

[37] Hussain A.I., Shanmugam V., Bhullar V.B., Beer B.E., Vallet D., Gautier-Hion A., Wolfe N.D., Karesh W.B., Kilbourn A.M., Tooze Z. (2003). Screening for simian foamy virus infection by using a combined antigen Western blot assay: Evidence for a wide distribution among Old World primates and identification of four new divergent viruses. Virology.

[38] Jones-Engel L., Engel G.A., Schillaci M.A., Rompis A., Putra A., Suaryana K.G., Fuentes A., Beer B., Hicks S., White R. (2005). Primate-to-human retroviral transmission in Asia. Emerg. Infect. Dis..

[39] Jones-Engel L., May C.C., Engel G.A., Steinkraus K.A., Schillaci M.A., Fuentes A., Rompis A., Chalise M.K., Aggimarangsee N., Feeroz M.M. (2008). Diverse contexts of zoonotic transmission of simian foamy viruses in Asia. Emerg. Infect. Dis..

[40] Shankar A., Sibley S.D., Goldberg T.L., Switzer W.M. (2019). Molecular Analysis of the Complete Genome of a Simian Foamy Virus Infecting *Hylobates pileatus* (pileated gibbon) Reveals Ancient Co-Evolution with Lesser Apes. Viruses.

[41] Wolfe N.D., Switzer W.M., Carr J.K., Bhullar V.B., Shanmugam V., Tamoufe U., Prosser A.T., Torimiro J.N., Wright A., Mpoudi-Ngole E. (2004). Naturally acquired simian retrovirus infections in central African hunters. Lancet.

[42] Callahan M.E., Switzer W.M., Matthews A.L., Roberts B.D., Heneine W., Folks T.M., Sandstrom P.A. (1999). Persistent zoonotic infection of a human with simian foamy virus in the absence of an intact orf-2 accessory gene. J. Virol..

[43] Rua R., Betsem E., Calattini S., Saib A., Gessain A. (2012). Genetic characterization of simian foamy viruses infecting humans. J. Virol..

[44] Schmidt M., Herchenroder O., Heeney J., Rethwilm A. (1997). Long terminal repeat U3 length polymorphism of human foamy virus. Virology.

[45] Shankar A., Smith J.M., Switzer W.M. (2025). Genome characterization of a simian foamy virus from a human bitten by an African green monkey. Microbiol. Resour. Announc..

[46] Saib A., Peries J., de The H. (1993). A defective human foamy provirus generated by pregenome splicing. EMBO J..

[47] Yu S.F., Stone J., Linial M.L. (1996). Productive persistent infection of hematopoietic cells by human foamy virus. J. Virol..

[48] Langmead B., Salzberg S.L. (2012). Fast gapped-read alignment with Bowtie 2. Nat. Methods.

[49] Nurk S., Bankevich A., Antipov D., Gurevich A.A., Korobeynikov A., Lapidus A., Prjibelski A.D., Pyshkin A., Sirotkin A., Sirotkin Y. (2013). Assembling single-cell genomes and mini-metagenomes from chimeric MDA products. J. Comput. Biol..

[50] Wick R.R., Judd L.M., Gorrie C.L., Holt K.E. (2017). Unicycler: Resolving bacterial genome assemblies from short and long sequencing reads. PLoS Comput. Biol..

[51] Chikhi R., Rizk G. (2013). Space-efficient and exact de Bruijn graph representation based on a Bloom filter. Algorithms Mol. Biol. AMB.

[52] Gurevich A., Saveliev V., Vyahhi N., Tesler G. (2013). QUAST: Quality assessment tool for genome assemblies. Bioinformatics.

[53] Camacho C., Coulouris G., Avagyan V., Ma N., Papadopoulos J., Bealer K., Madden T.L. (2009). BLAST+: Architecture and applications. BMC Bioinform..

[54] Kumar S., Stecher G., Tamura K. (2016). MEGA7: Molecular Evolutionary Genetics Analysis Version 7.0 for Bigger Datasets. Mol. Biol. Evol..

[55] Nguyen L.T., Schmidt H.A., von Haeseler A., Minh B.Q. (2015). IQ-TREE: A fast and effective stochastic algorithm for estimating maximum-likelihood phylogenies. Mol. Biol. Evol..

[56] de Vries D., Beck R.M. (2023). Twenty-five well-justified fossil calibrations for primate divergence. Palaeontol. Electron..

[57] dos Reis M., Inoue J., Hasegawa M., Asher R.J., Donoghue P.C., Yang Z. (2012). Phylogenomic datasets provide both precision and accuracy in estimating the timescale of placental mammal phylogeny. Proc. Biol. Sci..

[58] Martin D.P., Murrell B., Golden M., Khoosal A., Muhire B. (2015). RDP4: Detection and analysis of recombination patterns in virus genomes. Virus Evol..

[59] Troncoso L.L., Muniz C.P., Siqueira J.D., Curty G., Schrago C.G., Augusto A., Fedullo L., Soares M.A., Santos A.F. (2015). Characterization and comparative analysis of a simian foamy virus complete genome isolated from Brazilian capuchin monkeys. Virus Res..

[60] Ho S.Y., Phillips M.J., Cooper A., Drummond A.J. (2005). Time dependency of molecular rate estimates and systematic overestimation of recent divergence times. Mol. Biol. Evol..

[61] Wei G., Kehl T., Bao Q., Benner A., Lei J., Lochelt M. (2018). The chromatin binding domain, including the QPQRYG motif, of feline foamy virus Gag is required for viral DNA integration and nuclear accumulation of Gag and the viral genome. Virology.

[62] Hutter S., Zurnic I., Lindemann D. (2013). Foamy virus budding and release. Viruses.

[63] Lindemann D., Rethwilm A. (2011). Foamy virus biology and its application for vector development. Viruses.

[64] Lecellier C.H., Vermeulen W., Bachelerie F., Giron M.L., Saib A. (2002). Intra- and intercellular trafficking of the foamy virus auxiliary bet protein. J. Virol..

[65] Giron M.L., de The H., Saib A. (1998). An evolutionarily conserved splice generates a secreted env-Bet fusion protein during human foamy virus infection. J. Virol..

[66] Delebecque F., Suspene R., Calattini S., Casartelli N., Saib A., Froment A., Wain-Hobson S., Gessain A., Vartanian J.P., Schwartz O. (2006). Restriction of foamy viruses by APOBEC cytidine deaminases. J. Virol..

[67] Dynesen L.T., Fernandez I., Coquin Y., Delaplace M., Montange T., Njouom R., Bilounga-Ndongo C., Rey F.A., Gessain A., Backovic M. (2023). Neutralization of zoonotic retroviruses by human antibodies: Genotype-specific epitopes within the receptor-binding domain from simian foamy virus. PLoS Pathog..

[68] Lambert C., Couteaudier M., Gouzil J., Richard L., Montange T., Betsem E., Rua R., Tobaly-Tapiero J., Lindemann D., Njouom R. (2018). Potent neutralizing antibodies in humans infected with zoonotic simian foamy viruses target conserved epitopes located in the dimorphic domain of the surface envelope protein. PLoS Pathog..

[69] Rua R., Lepelley A., Gessain A., Schwartz O. (2012). Innate sensing of foamy viruses by human hematopoietic cells. J. Virol..

[70] Covey R., McGraw W.S. (2014). Monkeys in a West African bushmeat market: Implications for cercopithecid conservation in eastern Liberia. Trop. Conserv. Sci..

[71] Nasi R., Taber A., Van Vliet N. (2011). Empty forests, empty stomachs? Bushmeat and livelihoods in the Congo and Amazon Basins. Int. For. Rev..

[72] Mouinga-Ondeme A., Kazanji M. (2013). Simian foamy virus in non-human primates and cross-species transmission to humans in Gabon: An emerging zoonotic disease in central Africa?. Viruses.

[73] Patouillat L., Hambuckers A., Adi Subrata S., Garigliany M., Brotcorne F. (2024). Zoonotic pathogens in wild Asian primates: A systematic review highlighting research gaps. Front. Vet. Sci..

[74] Rua R., Betsem E., Gessain A. (2013). Viral latency in blood and saliva of simian foamy virus-infected humans. PLoS ONE.

[75] Roos C., Zinner D., Matsuda I., Grueter C.C., Teichroeb J.A. (2022). Molecular Phylogeny and Phylogeography of Colobines. The Colobines: Natural History, Behaviour and Ecological Diversity.

[76] Ting N., Tosi A.J., Li Y., Zhang Y.P., Disotell T.R. (2008). Phylogenetic incongruence between nuclear and mitochondrial markers in the Asian colobines and the evolution of the langurs and leaf monkeys. Mol. Phylogenet. Evol..

[77] Ghersi B.M., Jia H., Aiewsakun P., Katzourakis A., Mendoza P., Bausch D.G., Kasper M.R., Montgomery J.M., Switzer W.M. (2015). Wide distribution and ancient evolutionary history of simian foamy viruses in New World primates. Retrovirology.

[78] Katzourakis A., Gifford R.J., Tristem M., Gilbert M.T., Pybus O.G. (2009). Macroevolution of complex retroviruses. Science.

[79] Aiewsakun P., Katzourakis A. (2017). Marine origin of retroviruses in the early Palaeozoic Era. Nat. Commun..

