# Supplementary material for: Molecular Characterization of Complete Simian Foamy Virus Genomes from Three Colobine Monkeys Reveals Highly Divergent Evolutionary Trajectories and Identifies Transmission to Humans
Source: Viruses. 2026 Mar 4;18(3):320. doi: 10.3390/v18030320 (PMC13030746; doi:10.3390/v18030320)
Supplement: Supplementary file 1 [file viruses-18-00320-s001.zip › viruses-4136306-supplementary.pdf]

**Supplementary material.**

**Table S1. Primate and non-primate fossil calibrations for Bayesian inference of simian foamy virus time to most recent common ancestor**

| Clade | Split | Genome region | Prior distribution | Minimum bound | Maximum bound | Mean age (mya) | Std dev age (mya) | References |
|---|---|---|---|---|---|---|---|---|
| Crown Atelidae | Alouattinae-Atelinae | Fossil record | normal | 13.36 | 34.50 | 23.93 | 5.39 | De Vries and Beck 2023 |
| Crown Catarrhini | Cercopithecoidea-Hominoidea | Fossil record | normal | 25.19 | 35.10 | 30.15 | 2.53 | De Vries and Beck 2023 |
| Crown Cercopithecidae | Cercopithecinae-Colobinae | Fossil record | offset exponential | 12.47 | 25.24 | 18.85 | 0.00 | De Vries and Beck 2023 |
| Crown Cercopithecinae | Cercopithecinae-Colobinae | Fossil record | normal | 6.50 | 15.00 | 10.75 | 2.15 | De Vries and Beck 2023 |
| Crown Hominoidea | Hominidae-Hylobatidae | Fossil record | normal | 13.40 | 25.24 | 19.32 | 3.00 | De Vries and Beck 2023 |
| Crown Primates | Haplorhini-Strepsirhini | Fossil record | offset exponential | 55.94 | 66.10 | 61.02 | 0.00 | De Vries and Beck 2023 |
| Crown Colobinae | Colobini-Presbytini | Fossil record | normal | 8.13 | 15.00 | 11.56 | 2.10 | De Vries and Beck 2023 |
| Ungulata | Equine-bovine | Nuclear genes, mitochondrial genomes, fossil record | normal | 72.80 | 78.30 | 75.55 | 2.00 | Timetree.org |
| Boreoutheria (placental mammals) | Laurasiatheria-Euarchontoglire | Nuclear genes, mitochondrial genomes, genomes | normal | 89.92 | 102.10 | 96.0 | 3.10 | Dos Reis *et al.* 2012 |
| Crown *Macaca* | *M. sylvanus*-Asian macaques | Complete genomes | normal | 2.81 | 4.14 | 3.47 | 0.33 | Shao *et al.* 2023 |

1. Mya, millions of years Ago. Mean age determined from proposed minimum and maximum bounds with standard deviations (Std Dev) in BEAST used as the 2.5% and 97.5% quantiles of the prior distributions for the minimum and maximum bounds, respectively.

**Figure S1.** Nucleotide and amino acid sequence alignment of the SFVcgu_910916, SFVpne_500057, and SFVtfr_083616 genomes. Nucleotide identities are shown with dots; deletions are shown with dashes. Locations of the LTR regions are provided with black brackets. Separation of the U3, R, and U5 regions in the long terminal repeats (LTRs) are indicated with purple arrows. The group specific antigen (Gag), protease-polymerase (Pro-Pol), envelope (Env), transcriptional transactivator (Tas), between Env and Tas (Bet) protein sequence initiation and termination codons are labeled and highlighted with green and orange boxes, respectively. All nucleotide motifs and important signals are labeled and shown with pink, blue, and green boxes, respectively. PBS, primer binding site; IP, internal promoter; DS, dimerization signal (DS3 surrounds the Gag start codon and is not shown); PPT, poly purine tract. The splice donor (SD) and acceptor (SA) nucleotide sequences and location for the Bet coding regions are shown with bold text and arrows.

**Figure S2.** Group-specific antigen (Gag) amino acid alignments of SFVcgu_910916, SFVpne_500057, and SFVtfr_083616 with five Asian and five African SFV. Amino acid identities are shown with dots; deletions are shown with dashes. The cytoplasmic targeting and retention signal (CTRS), assembly domain with the YXXL motif, the conserved PSAP motif, and the glycine-arginine rich (GR) boxes are labeled and highlighted with blue boxes. The black box within CTRS shows the highly conserved arginine reside (R). The black box within GR2 shows the location of the chromatin binding sequence. The cleavage signal (CS) is shown with a purple arrow.

**Figure S3.** Polymerase (Pol) amino acid alignments of SFVcgu_910916, SFVpne_500057, and SFVtfr_083616 with five Asian and five African SFV. Amino acid identities are shown with dots; deletions are shown with dashes. The protease (Pro) and reverse transcriptase (RT) active centers (AC), integrase (IN) zinc binding motifs (BM), and IN active centers (AC) are labeled and highlighted with blue boxes. The cleavage signal (CS) is shown with a purple arrow.

**Figure S4.** Envelope (Env) amino acid alignments of SFVcgu_910916, SFVpne_500057, and SFVtfr_083616 with five Asian and five African SFV. Amino acid identities are shown with dots; deletions are shown with dashes. The locations of the leader peptide (LP), surface subunit (SU), and transmembrane (TM) domain are indicated surrounding the furin cleavage sites (CS) shown with a purple arrow. The conserved N-terminus signal peptide, fusion peptide, membrane spanning domain (MSD) and endoplasmic reticulum (ER) retention signals are labeled and highlighted with blue boxes. The highly conserved WXXW motif required for Gag interaction and budding is shown with a black box inside the signal peptide.

**Figure S5.** Transactivator (Tas) amino acid alignments of SFVcgu_910916, SFVpne_500057, and SFVtfr_083616 with five Asian and five African SFV. Amino acid identities are shown with dots; deletions are shown with dashes. The conserved bipartite nuclear localization signal (NLS) is labeled and highlighted with a blue box.

**Figure S6.** Between envelope and transcriptional transactivator (Bet) amino acid alignments of SFVcgu_910916, SFVpne_500057, and SFVtfr_083616 with five Asian and five African SFV. Amino acid identities are shown with dots; deletions are shown with dashes. The conserved integrin-binding motif (K/RGD) is labeled and highlighted with a blue box. The Tas sequence of SFVmcy_FV34 has been reported to have a premature stop codon at residue 260.

**Figure S1.**

```
                   10        20        30        40        50        60        70        80        90        100
               ....|....|....|....|....|....|....|....|....|....|....|....|....|....|....|....|....|....|....|....|
SFVcgu_916910  TGTGGTGGACAGCCACTAGAATAATAGGTCCAAAAGAAGAGCACTACTGGAAAGAATGCCAGGAGCATGGTT------ATTTTCCTTTAGTTCCTGATCC 94
SFVpne_500057  .....CA.GTT..A..A.A.TGT..T...ATT........AG.T..T...TGT....ATG.TC.TAGA..A.------........CACA.A..CA..AA 94
SFVtfr_083616  ........TTA......A......T..AG....T.T...TG...........CCT..GGGA...T...AA.TGGATG.......AG.G..A..AA..AA 100

                   110       120       130       140       150       160       170       180       190       200
               ....|....|....|....|....|....|....|....|....|....|....|....|....|....|....|....|....|....|....|....|
SFVcgu_916910   TAAAAAACCAACCTGGGTGAGACATTTAGCTCCTTTTAAGGTTACCCCTTTCACTACTCCAGTTGATCTCCAAAAAATTGTGAATGAAAGTCTTCCTTAT 194
SFVpne_500057   A.TGTCC..T..T...........G.T..C..C.GC..CA.ACAAAGA...G.A..A...CA.........G.TT.A.CA.....GCT.T.A..A.C. 194
SFVtfr_083616  AC.T..GGTG..T...C.TGCG...AC.C.A.AGA.....C.AT.A.....TG........TC....G.....G.CTA.......TT..C.T.A..AG.G 200

                   210       220       230       240       250       260       270       280       290       300
               ....|....|....|....|....|....|....|....|....|....|....|....|....|....|....|....|....|....|....|....|
SFVcgu_916910   CAGTATGGAATAATAACTCGTGATGGAAAGATTTACAAAAGTGAGAAAAAAGATAATTTGTTCAAATATGGGACCTTGGAAGAATACTGGGACAATGAGG 294
SFVpne_500057   GG..T.A.T....CC..C.CA..G.....TTG...T.C...CAGTCGC.G.CTCC...ATGGA..TG.A..A..TC.AC....G.TTCAA..TG..T.TA 294
SFVtfr_083616  A.T...AA.G.GG.G..A.CCAG....G.AGAA..TGTT..CA.C.G...G.TA..CA.TG.G.G.G..CTC..T.G.AGT...ATTAAA.CTTC.---- 296

                   310       320       330       340       350       360       370       380       390       400
               ....|....|....|....|....|....|....|....|....|....|....|....|....|....|....|....|....|....|....|....|
SFVcgu_916910   AAGAGACAAAGAAACAGTTTAATTGTGATTGGCTTGATTCTGATAGTGATGATTATTCAGGGGAAGACTAGAATTAGAACGAGGAAATGCCTAGCAACTG 394
SFVpne_500057   .CAGA.TC..A.G.A.A.---------..CAAAA..GA...TC......G.---.G....AT.....T.GA...---------ATTC............. 373
SFVtfr_083616  --------------A.......GAA.......AGAT.CAT..CAGA......CA..C.T.CA..T..T.G.T.----------A..TAC.....T...AA 372

                   410       420       430       440       450       460       470       480       490       500
               ....|....|....|....|....|....|....|....|....|....|....|....|....|....|....|....|....|....|....|....|
SFVcgu_916910   CTTATGCTTAATTATGAATCATAGTATTGTTTAAGTTAAGAGTTAAGAAATACATAGTTAATCCTTAGGGAGCATTTGGTATAACCTACTAATAG-ACAA 493
SFVpne_500057   .........GC......C.A..GCCT......GGAA.....A.AT.TTT.GCT...............A...........A.TT.......GTA-.GGC 472
SFVtfr_083616  A.G...A.AG.A...C.TAA.-----------...A.....A.GCTTCTGCTTCC..............-..........CT...T.AG.A..GTAT.T.. 460

                   510       520       530       540       550       560       570       580       590       600
               ....|....|....|....|....|....|....|....|....|....|....|....|....|....|....|....|....|....|....|....|
SFVcgu_916910   GAGTTGTTTACTACCGTGTTCCCAATCGGAGACT-TGGGGTGAGGATGTTAGTTCAAATCCATGCTCAGCCTAAGTGTCTCCCTTTTA------------ 580
SFVpne_500057   TGT.CA.........A..C.T...G.G.AGA...CC....C....C...G.....G.G.T..AC....T.TCG.............GCTTTTGCTTTCA 572
SFVtfr_083616  CT...C..A.ACC.G...C....GGAA.A.CT..TCT..AGA.T..C.--G....G.G..--GCA.TCT..C..AG.AG.T.T....CTTTTTTCTT--- 553

                   610       TATAA     630       640       650       660       670       680       690       700
               ....|....|....|....|....|....|....|....|....|....|....|....|....|....|....|....|....|....|....|....|
SFVcgu_916910   ------------------GTATAAGATAAGTTTTTGCTGTCA------------GCTAATCCTAAGGGAGGAAGT-GGACCTCTTGATAAATATCAAT-- 647
SFVpne_500057   TAAATAAATAAAGAATATA.......GT..GA.AA.T.ACT.------------TT.............A...A.G....G.........GACA..G--- 657
SFVtfr_083616  -------------AATAGT.....TTGT...G..AAG.AGG.TAAGAAACTTGT.T.......T..T..AAG...G.CTT.C........TGTG.T.ACC 640

                   710       720       730       740       750       760       770       780       790       800
               ....|....|....|....|....|....|....|....|....|....|....|....|....|....|....|....|....|....|....|....|
SFVcgu_916910   TCAGGATGTGAACTTATCTCCCTT--TTAGTGTGTTCAAGCTTTGCTTACGTTTTCTGACTCA------------TAG--CCAGATATAAAATAAAGTGT 731
SFVpne_500057   -..A..C..TCC.C.CCTCT.T..TC..TC....A....ATC.AAA.A.A.AA.GT...T...CTGCCAGAACAT...--..T..AG.........C.AG 754
SFVtfr_083616  ..GA..GAA.CG.CGC...TT.A.--..T.A..T.GA.GA.CGCAAGATT.CA.CA..GT.T.GAAACAG-CTAT...CT.T...A.C.GG.AG..CCT. 737

                   810       820       830       840       850       860       870       880       890       900
               ....|....|....|....|....|....|....|....|....|....|....|....|....|....|....|....|....|....|....|....|
```

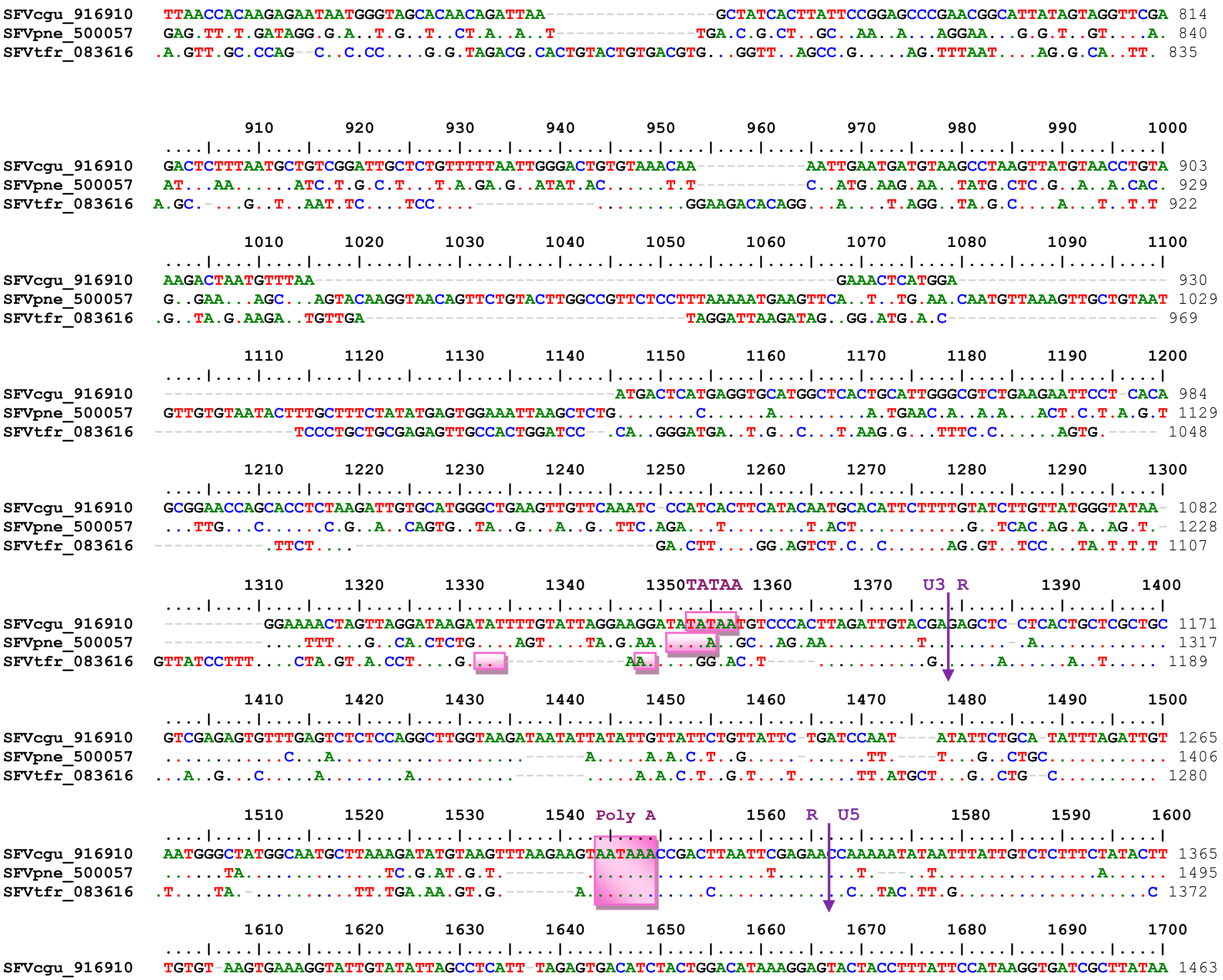
SFVcgu_916910
SFVpne_500057
SFVtfr_083616
TATAA
U3 R
Poly A
R U5

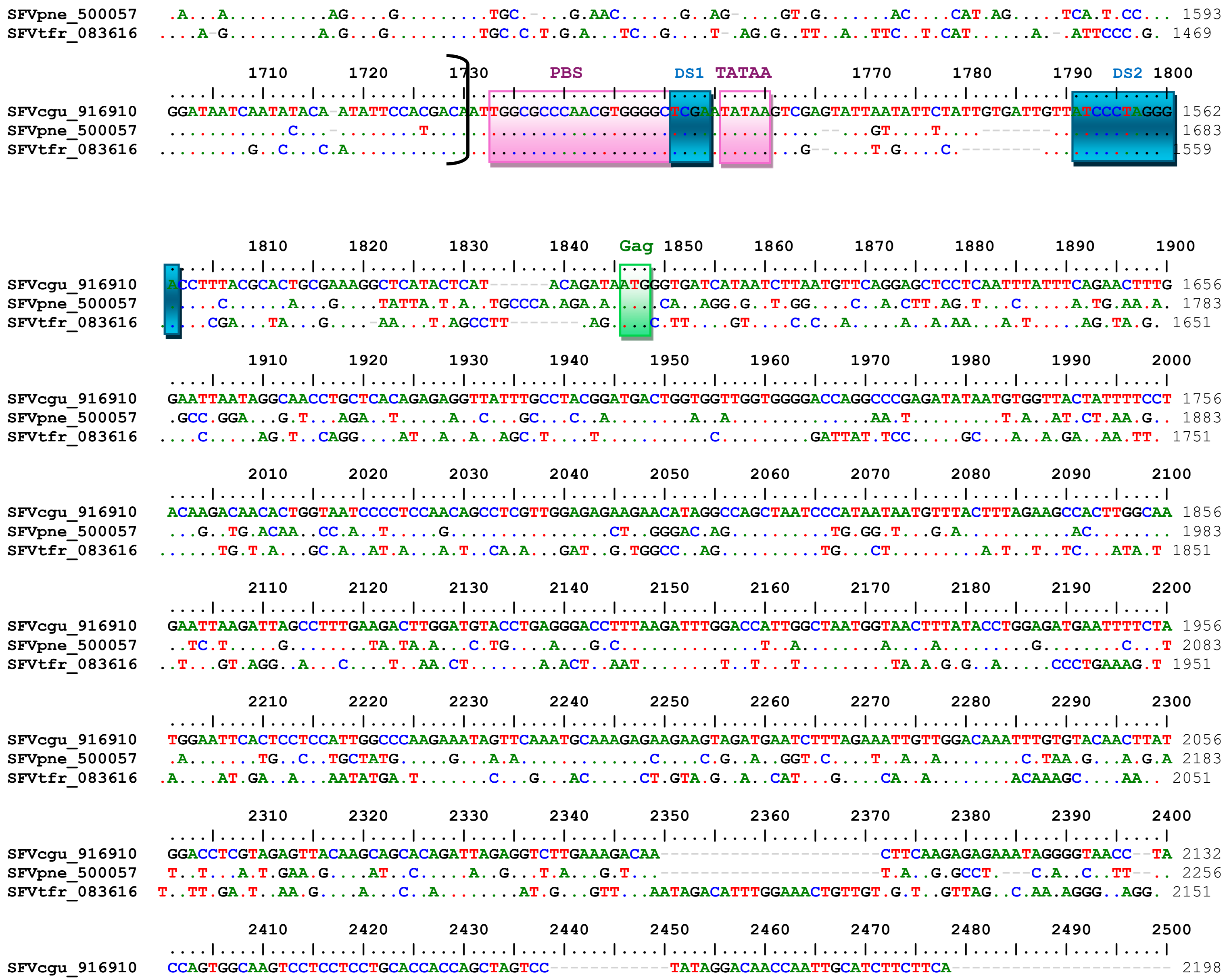
SFVpne_500057
SFVtfr_083616
SFVcgu_916910
PBS
DS1
TATAA
DS2
Gag

```
SFVpne_500057   .....T..T.A.AT...A...C.......TC.....TT------------AGATTTT..G..TGAA..CG........CCTTATGTAGCTCCTGCTGCTC 2344
SFVtfr_083616  G....A..CCC.GT.G.A.T.AG.....A.A.ACCA..AGGGCCATCAGGA......GG...T..G..TA.A..AA.GCCTGGAGA--------------  2237

                        2510      2520      2530      2540      2550      2560      2570      2580      2590      2600
                ....|....|....|....|....|....|....|....|....|....|....|....|....|....|....|....|....|....|....|....|
SFVcgu_916910   ---GTGCAACCACCTATTGATTTAGGTTG----------GGGACAAGTTCCACCTGATAATATAGATTCTCGATTATCAAGAGTTGCTTATAATCCTTTT 2285
SFVpne_500057   CTG.....G..G.-----...C..G..A..GTTTGCTGGT...C.TG..C.GT...T.----G.G..CC.AA..C.GG.TC.C..A..A..C........C 2436
SFVtfr_083616  -----------------A.G......A..GCAGGCAAA---------------..C.C..-G.T...C.AA.GC..ATG..G..A..C.....C...AA. 2304

                        2610      2620      2630      2640      2650      2660      2670      2680      2690      2700
                ....|....|....|....|....|....|....|....|....|....|....|....|....|....|....|....|....|....|....|....|
SFVcgu_916910   TTAC---CTGGACCAAATGATGGAGGAGGATCTATCCCGGTTCAGCCAAGTGCTCCTCCTGCGATATTTCCTTCACCTCAACCTGTTCCAGCT------G 2376
SFVpne_500057   ..G.---....T....G..G...TTCT.AGG.AGC...........T..............GCT.CC..A.TG.TA.C..TGCCA......CAGCCA. 2533
SFVtfr_083616  .GG.CAG.A.ATGGC.GCAG.AAT.CT..GGTATC..AA..GGC...T.....A.....A.....GCCC...G..AT..CTG....AT.T.T.TTGCC-2402


                        2710      2720      2730      2740      2750      2760      2770      2780      2790      2800
                ....|....|....|....|....|....|....|....|....|....|....|....|....|....|....|....|....|....|....|....|
SFVcgu_916910   TTCATCCAGTTATTCAATATGTGGCACC------------TCAACCTGCTCCACAAGTTATACCTATTCAACATATTAGAGCTGTTGTTGGAGATACTCC 2464
SFVpne_500057   .A..G..TA..............CAT..ACC---GCTTAT.A.T.......A.......A.....A..A..G...........A..CACC...TC.G.C.. 2630
SFVtfr_083616  -------------------..C.AT...TCCCATGGCTGT.....A.C..GG..CT..C..T.....A..........A...A...AC.......G.C.. 2483

                        2810      2820      2830      2840      2850      2860      2870      2880      2890      2900
                ....|....|....|....|....|....|....|....|....|....|....|....|....|....|....|....|....|....|....|....|
SFVcgu_916910   AAAGAATCCCCAAGAAATTCCTATGTGGATAGGAAGACATGCTCCAGCAATAGAAGGAGTCTTCCCAATTACAACACCTGATCTACGTGCCAGAGTGGTT 2564
SFVpne_500057   ..CA..C..A.G...G..A.........G.T......A....CT.T..C...........G..T..C..AC.T..T.....CA..A.ATGT.....TA.. 2730
SFVtfr_083616  TCGA....TA.G......C..C..........CC.....C........T..T......A.A.AT..TG.AG.T.....A...A..A.AT.A...A.AA.. 2583

                        2910      2920      2930      2940      2950      2960      2970      2980      2990      3000
                ....|....|....|....|....|....|....|....|....|....|....|....|....|....|....|....|....|....|....|....|
SFVcgu_916910   AATGCTCTGATTGGAGGATCTCTTGGATTGGCCGTTGAGCCTATTCATTGTGTATCCTGGGCCGCAGTTGTAGCAACATTATATGTCAGAACTCATGGTT 2664
SFVpne_500057   ........AC.........AA.......C.TA.TT.A..A...CAA.....CA..A.T......T...C.A....C...C.T..................A 2830
SFVtfr_083616  .....AT..G.....CA.---T.A...C.T..AT.AAGTATA.A.G.G..CA.TA.A....AA..T.CG......GTT..G.T...TC....C.....AC 2680

                        3010      3020      3030      3040      3050      3060      3070      3080      3090      3100
                ....|....|....|....|....|....|....|....|....|....|....|....|....|....|....|....|....|....|....|....|
SFVcgu_916910   CTTACCCTGCTCATCAGTTAGGGAACATATTGCAAGATATCTGCAGGCAAGAAGGAGTTGCTACAGCTTTTCAATTGGGAATTATGCTATCACAAAATAA 2764
SFVpne_500057   .A.....CCT......AC...CTG.AG.G..AAG..GAG.G.CT.ACTCT...........AG.T..A.GG.....A.....G.....TA.TA.TC.AG. 2930
SFVtfr_083616  A.......CTC......C.T..AG.AG..A.C....GA..AGT..CT........G.CA..A..T....A.AGT..A.....G...T..A.TG.T..... 2780

                        3110      3120      3130      3140      3150      3160      3170      3180      3190      3200
                ....|....|....|....|....|....|....|....|....|....|....|....|....|....|....|....|....|....|....|....|
SFVcgu_916910   TTGGGAGCTTGTTTGGGGAATCTTAAGGCCCCAGTTACCTGGTCAAGCTGTAGTTACAGCTATGCAACATAGATTGGACTTAGAAGTCAATGATCCTGCT 2864
SFVpne_500057   C.ATA.C...A..........TG.T..A..TTTA..G.....A........T.....T...........CC.GC.....CA......G.G......A..A 3030
SFVtfr_083616  ..TT...T.G..A.........A.........AT.C.......A................A..T.......A........T..G..GCCA...C.A.AACAA 2880

                        3210      3220      3230      3240      3250      3260      3270      3280      3290      3300
                ....|....|....|....|....|....|....|....|....|....|....|....|....|....|....|....|....|....|....|....|
SFVcgu_916910   AGAATAGCATCCTTTGTTGCTCATTTAAATGCTGTTTATGAACTGTTGGGGCTAAATGCTCGAGGACAAAGTATAT---------ATAGACAAC------ 2949
```

```
SFVpne_500057    ...G.G.TT..T...A..AAC..CC.........A.......C..A..CT.G..C..CA.........CC.------------....AC..CTGTTT 3118
SFVtfr_083616   ..GGC..GCAGA...C.GA.A.....C...AA..A...TCT..AC....C................G.....CGAGCATCTGC.G...CC.GCAACT 2980

                         3310      3320      3330      3340      3350      3360      3370      3380      3390      3400
                 ....|....|....|....|....|....|....|....|....|....|....|....|....|....|....|....|....|....|....|....|
SFVcgu_916910    ---AAGGACAACAATCATCTGGAGCTTCTTCTACAGGAAGGTCTAATAGAGGACGAAGAAATCAACAATCCTCTCCTAGACAACA---------ACAATC 3037
SFVpne_500057    CTGGG......ACTA.GG....GA.C...G..GG.A..G.---..C.C.G..T..T....G...G...GGAG.C...G.GAG...ATCTTCAGG.....T 3215
SFVtfr_083616   CCAC....A.C...C.TC.ACC.AG.AGAGG.GG.CA.G.---A.GAG.......T..G.GATC.GG.GGAAA....CC..G.A.--------TC...CT 3069

                         3410      3420      3430      3440      3450      3460      3470      3480      3490      3500
                 ....|....|....|....|....|....|....|....|....|....|....|....|....|....|....|....|....|....|....|....|
SFVcgu_916910    GCAACAAAATCGAAGAAATATTCAAAATAATCAAGGGACACAATCTGG------TGGCTCTAATAAC------------CAGAATAGTCAA---AGACAG 3116
SFVpne_500057    A....C.C.AG......------G..G.TCC.....---....C..A.ACAGGT..AT...GGCG.T------------TC....CAA..G---..G... 3291
SFVtfr_083616   .----------------------G..T.GG.AG...---....AA..A------.AATAA......TACCTCTTCAAGA...G...CA.CGCCC...... 3138

                         3510      3520      3530      3540      3550      3560      3570      3580      3590      3600
                 ....|....|....|....|....|....|....|....|....|....|....|....|....|....|....|....|....|....|....|....|
SFVcgu_916910    TC---------------CTCTGGAGGATACAACTTACGTCCCAGAATCTACCAACCTCAACGATACGGCGGAGGCCCAGGGAGAAGATGGAATAACAATA 3201
SFVpne_500057    A.GCAAGGAGGGAATGGTAGA........T..T...A.G..AC.T.CG........A...A.G.....G..C..A.G...CC.CC........G.TC.GC 3391
SFVtfr_083616   ..------TGGAAACCAACGA..T..T..T..T...A.G..AC.T.C..TT......ACT........A.....AAG...AC.TC.G...G.....C.-- 3230


                         3610      3620      3630      3640      3650      3660      3670      3680      3690      3700
                 ....|....|....|....|....|....|....|....|....|....|....|....|....|....|....|....|....|....|....|....|
SFVcgu_916910    CTCAACAGCAGCAGCAGTCACCCAGCACACAGTCTGGTGATCAACGGCGTTCCCAAGGCCAGCAACCGCAATCAGAGGCTCGTGGCGATCAGTCACGAAC 3301
SFVpne_500057    ..------------------------GT.AGA..A.A.A......AA........GTCG........A..G.......T.........G........... 3467
SFVtfr_083616   ------------..G......-----...A.A.TCA.A.TCT.---.A.C.......A.TTCT...A.T.CGC...GA.GC.....T.....A.....TC. 3311

                         3710      3720      3730      3740      3750      3760      3770      3780      3790      3800
                 ....|....|....|....|....|....|....|....|....|....|....|....|....|....|....|....|....|....|....|....|
SFVcgu_916910    ATCTGGAAGCGGACGTGGGCCACAAGGAAGAGGAACTCAAAACCGAAACCAGCGTCCTGTCAATGCAAACAG---TGCTCAACCTGTGAATACGGTGACT 3398
SFVpne_500057    ......CGCG..G.....A.A.GG........AG.AC.................ATTAACTGG..G....GC---CAACAG.A..........A.....- 3563
SFVtfr_083616   C......G.T..C..A..A.A.GGC.....TCT.A...............A..A....GAGGA.T..G.G.TGA.A...GCA....C..CG.T...... 3411

                         3810      3820      3830      3840      Pol       3860      3870      3880      Gag       Gag3900
                 ....|....|....|....|....|....|....|....|....|....|....|....|....|....|....|....|....|....|....|....|
SFVcgu_916910    CAGATTTCTGGTGCTGCAACCACATCA---------TCAACAAATAATGAATCCACTTCAACTTCTTCAGCCTCTGGAGGCAGAAATTAAAGGTCATAAA 3489
SFVpne_500057    -----------A......T..G.C...GTCCCTGCC...GGTC.A....G....T..C..G..C..............A..G.....C..G..AAC.... 3652
SFVtfr_083616   ..ATC---------------.....C..--------CC...T.TG.A....G.....G.A.....GA.G..A...........G.....A..GAAAAC.... 3490

                         3910      3920      3930      3940      3950      3960      3970      3980      3990      4000
                 ....|....|....|....|....|....|....|....|....|....|....|....|....|....|....|....|....|....|....|....|
SFVcgu_916910    CTAAAAGCACAGTGGGATAGTGGAGCCACAATTTCCTGTGTTCCTAAGTCATTCCTAACAGATGAACAACCTATTAAACATATGAAGATAAAGACTATTC 3589
SFVpne_500057    T.......C..C.....................A.T.....C...G.AGT......TGA.......T.T.....AC.GACC...TT...T..A....... 3752
SFVtfr_083616   ..TTTT.....C.....C.....TA....C..AA....C..C..AGCTG....T..CT.C..G...G....A...TC.G.AGCA..A..T........A. 3590

                         4010      4020      4030      4040      4050      4060      4070      4080      4090      4100
                 ....|....|....|....|....|....|....|....|....|....|....|....|....|....|....|....|....|....|....|....|
SFVcgu_916910    ATGGAGAGAAACTTCAACCAGTTTATTATTTGACTTTTAAAGTTCAAGGTAAAAAAGTGGAAGCAGAAGTTATAGCTTCCCCTTATGACTATATTTTATT 3689
```

```
SFVpne_500057   .......A....AA..GAAT...........A.........A.A.....A.G......A...........AC.T..C.....A.........G.....C. 3852
SFVtfr_083616  ........C....A...AA...A.....C..A..A.........C.T...A.G......A..G........A...A....T........T.....AC.T.. 3690

                          4110      4120      4130      4140      4150      4160      4170      4180      4190      4200
                ....|....|....|....|....|....|....|....|....|....|....|....|....|....|....|....|....|....|....|....|
SFVcgu_916910   AAGTCCTTCGGATGTACCTTGGTTAATGAAGCAACCATTACAATTGACTATTTTAGTTCCTTTACAAGAATATCAAGAAAGAATATTACAAAAAACAACC 3789
SFVpne_500057   ......A..A.....T..C...C.T......A....T............G.GC........C.......T............C.T..GA..C....TG.T 3952
SFVtfr_083616   ..A...AA.A.....T............C.AA....T..G............A.....A..AC.T..........A...CT.....TC.......G..TG.T 3790

                          4210      4220      4230      4240      4250      4260      4270      4280      4290      4300
                ....|....|....|....|....|....|....|....|....|....|....|....|....|....|....|....|....|....|....|....|
SFVcgu_916910   TTGCCAACACAAGAGAGAGAAAAACTTAAAACTTTATTTATTAAATATGAACTATTATGGCAACATTGGGAAAATCAAGTAGGACATAGGCATATTTTGC 3889
SFVpne_500057   .....T.AGG..C.A.A...TC.GT.A..T.AAC.G...T.A....T...TGC.C.C...............................AGA...AA.G 4052
SFVtfr_083616  C.AAA.GA.G.CT.C.A.C..C.GT.AC...AAC.TC..GA.........CTCC.................G........G..T......AGA..ACCTG 3890

                          4310      4320      4330      4340      4350      4360      4370      4380      4390      4400
                ....|....|....|....|....|....|....|....|....|....|....|....|....|....|....|....|....|....|....|....|
SFVcgu_916910   CTCATAATATTGCTACTGGTACAGTTACTCCTAGGCCTCAAAAACAATATCCTATTAATCCAAGAGCTCGAGAGAGTATACAAATTGTCATTAATGATTT 3989
SFVpne_500057   .A..........A.........T.AG.C.................G........A.......AG..AAA.CCT..C............A..AG....... 4152
SFVtfr_083616  .A........A..A..A..ACA.AC.C.....C.C............G.....A........T.A...GAA.CCT.....T........G..AG...... 3990

                          4410      4420      4430      4440      4450      4460      4470      4480      4490      4500
                ....|....|....|....|....|....|....|....|....|....|....|....|....|....|....|....|....|....|....|....|
SFVcgu_916910   GTTAAAACAAGGGGTTTTAATACAACAAAACAGTATCATGAACACTCCTATATACCCTGTACCTAAACCTGATGGAAAATGGAGAATGGTTCTAGATTAC 4089
SFVpne_500057   ..............................T..C.CT.....T..C..A.....T..A.....A..G..C.....T........G.....GT.......T 4252
SFVtfr_083616  .C.......G..T..G...G.G.......CA..C..T.....T......G....T..A..T.....G..A.....T.G............GT.......T 4090


                         Poly A    4520      4530      4540      4550      4560      4570      4580   TATAA          4600
                ....|....|....|....|....|....|....|....|....|....|....|....|....|....|....|....|....|....|....|....|
SFVcgu_916910   AGAGAAGTTAATAAAACTATACCCCTAATAGCTGCTCAAAACCAGCATTCTGCAGGTATATTATCAACTATTTTTAGAGGAAAGTATAAGTCTACTTTAG 4189
SFVpne_500057   ..G..................C..AT....T..C..A..G..T..A.....A.........TC.T..CT.A..C.A.................A......... 4352
SFVtfr_083616  ........A........A..T..TT.......A..A.....T..A..C...TT...A.....G..T.G.....A..........A.....AA.A....... 4190

                          4610      4620      4630      4640      4650      4660      4670      4680      4690      4700
                ....|....|....|....|....|....|....|....|....|....|....|....|....|....|....|....|....|....|....|....|
SFVcgu_916910   ACTTATCAAATGGATTCTGGGCACATCCTATTACCCCGGAATCCTACTGGTTAACTGCATTTACTTGGCAAGGGAAACAATATTGTTGGACAAGACTACC 4289
SFVpne_500057   .TC.TA..........T.....T........A..A..A.....T...........A..T...................................T..GT.... 4452
SFVtfr_083616  .T.....T.....T...................A.....A.....G..T...A....A..C.......................................C.. 4290

                          4710      4720      4730      4740      4750      4760      4770      4780      4790      4800
                ....|....|....|....|....|....|....|....|....|....|....|....|....|....|....|....|....|....|....|....|
SFVcgu_916910   TCAAGGGTTTCTTAATAGTCCTGCTTTATTTACTGCAGATGTAGTTGACTTATTAAAAGACATACCTAATGTGGAAGCATATGTTGATGATATTTATATT 4389
SFVpne_500057   A.....A...........................A........T.....T..G.....G..A.....A.....TC..........G...........C... 4552
SFVtfr_083616  ......A..............A.....G.....A........G..A..T..........A.T..T........AC.T..T...................G.A 4390

                          4810      4820      4830      4840      4850      4860      4870      4880      4890      4900
                ....|....|....|....|....|....|....|....|....|....|....|....|....|....|....|....|....|....|....|....|
SFVcgu_916910   AGTCATAATGACCCACAAGAACATTTAGAGCAACTTGAGAAAGTTTTCTCAATATTGCTCAATGCTGGTTATGTGGTTTCTCTTAAAAAATCTGTTATTG 4489
```

```
SFVpne_500057   .....G......T.........C.T..A........A........T..T.....A.......................C..C.....G....AA.... 4652
SFVtfr_083616  .....G....T..TTCT.....C.G..A..GT.A..A...A.A..TGATG....A.AA.......G...........GT.A.....G..A.AG.... 4490

                        4910      4920      4930      4940      4950      4960      4970      4980      4990      5000
                ....|....|....|....|....|....|....|....|....|....|....|....|....|....|....|....|....|....|....|....|
SFVcgu_916910   CTCAATATGAAGTTGAATTTTTAGGTTTTAATATTACTAAAGAAGGTAGGGGCCTTACTGACTCCTTTAAACAGAAGCTGTTAAATATTACCCCACCTAA 4589
SFVpne_500057   .A...AGA...................................C.T........A..TA.T........A..AT..........A.T......... 4752
SFVtfr_083616  .......CTCT..G...........G........C...........G..A.....A..A..A..T......A.T..AA.T.....C..AT.A........ 4590

                        5010      5020      5030      5040      5050      5060      5070      5080      5090      5100
                ....|....|....|....|....|....|....|....|....|....|....|....|....|....|....|....|....|....|....|....|
SFVcgu_916910   AGATCTTAAACAATTACAAAGTATATTAGGATTGTTAAATTTTGCTAGAAATTTTATTTCTAATTTTTCTGAATTAGTTAAACCACTTTATAATTTGGTA 4689
SFVpne_500057   ....T.A..G..G........C..T......C....G.................C...C..................A......T.G..C.C.A.T... 4852
SFVtfr_083616  .....A.....G........C..TC.T.....AC.......................C........G....G..GA.A..G...T.A.........A.T 4690

                        5110      5120      5130      5140      5150      5160      5170      5180      5190      5200
                ....|....|....|....|....|....|....|....|....|....|....|....|....|....|....|....|....|....|....|....|
SFVcgu_916910   GCAACTGCTAAGGGTAAATATATTGAATGGACTACTGAACACAGTAATCATTTACAATATATAATTACTGTACTTAATTCTGCTGAGAATTTGGAAGAAA 4789
SFVpne_500057   ..C.A...C..T.......T...ATCC...T.AGAA...A.T........A.....GA....T.....A..T..A...CAG..A..A.....A....... 4952
SFVtfr_083616  .....A..A..T..G.........TC....T..GAG...A.TTC...G..G......G.AC.G.....AAC.T.A....TG..A..A.....A..G...C 4790

                        5210      5220      5230      5240      5250      5260      5270      5280      5290      5300
                ....|....|....|....|....|....|....|....|....|....|....|....|....|....|....|....|....|....|....|....|
SFVcgu_916910   GAAATCCTGAAACTAAATTAATTATTAAAGTTAATTCTTCTCCATCAGCAGGCTACATAAGGTTCTACAATGAACATTCTAAAAGACCTATTATGTATGT 4889
SFVpne_500057   .............A.G.C.T..A..C.....A.....C.....C..T.....A..T..T..A.AT..T......GGG....G.C....A........... 5052
SFVtfr_083616  ....A..A..TGTACGT...G.A..A..GA.C...G.C..C..T..T..T..A.....CC.C..T..T......GG...AC...A...C..C......A. 4890

                        5310      5320      5330      5340      5350      5360      5370      5380      5390      5400
                ....|....|....|....|....|....|....|....|....|....|....|....|....|....|....|....|....|....|....|....|
SFVcgu_916910   AAATTATGTATATACAAAAGCTGAACTTAAATTTACCCCTACTGAAAAATTATTATCAACTATCCATAAGGCCCTAATTAAAGCCATGGATTTAGCCATG 4989
SFVpne_500057   T........G.T.T.T..G......G....G.....T.AA..A.........C..A.T..A..G.......GT..T.....G........CC.T..T... 5152
SFVtfr_083616  .........T.T...T..GA.A..GT.A.........T..ATTA.....G..G...A....CC.A.....A..TT.......G..TT.A...C.G..T... 4990

                        5410      5420      5430      5440      5450      5460      5470      5480      5490      5500
                ....|....|....|....|....|....|....|....|....|....|....|....|....|....|....|....|....|....|....|....|
SFVcgu_916910   GGACAAGAGATTATAGTTTATAGTCCTATTGTTTCTATGACAAAGTTACAAAGGACTCCTCTTCCTGAAAGAAAAGCTTTACCCATACGATGGATTACTT 5089
SFVpne_500057   ..T..G..A..C.....A.........A..A..........C..AA.T..G.AA..A..AT.A...............A.....TG.CA.G.......... 5252
SFVtfr_083616  ..T..G..A..ATCTA.C..C............G.............A.T....AA......T.A.....G...............T..TA.......A..C. 5090

                        5510      5520      5530      5540      5550      5560      5570      5580      5590      5600
                ....|....|....|....|....|....|....|....|....|....|....|....|....|....|....|....|....|....|....|....|
SFVcgu_916910   GGATGACTTATTTAGAAGATCCACGAATACAGTTTCTATATGACAAAACTTTGCCTGAACTACAACAAATACCTATTAT---CACTGAAGATGTTATAGC 5186
SFVpne_500057   ......A...........C..CA.G..T..A...TAT.....T....G.C.A..A...T..........T...TCAG.---.............AG.G.. 5349
SFVtfr_083616  .......A.....G.........TA....TA.....TAT...........A......A...T..A....T..C...GAAG.ATCT....G.AT....CA.-- 5188

                        5610      5620      5630      5640      5650      5660      5670 Poly A         5690      5700
                ....|....|....|....|....|....|....|....|....|....|....|....|....|....|....|....|....|....|....|....|
SFVcgu_916910   TAAGTCTAAACACCCCAGTGAGTTTGAAATGGTATTTTATACTGATGGTTCTTCAATTAAACATCCTAATATTAATAAAACACATAGTGCTGGTATGGGA 5286
```

```
SFVpne_500057     A..AA.......T..T.....A...TC......C..............G...G.T..C............G........T.T...........C...... 5449
SFVtfr_083616     -------.....T...TCAC.A.A.AGT..............C........AG.T......A.......CCC.......G..........C..A...... 5281

                        5710      5720      5730      5740      5750      5760      5770      5780      5790      5800
                  ....|....|....|....|....|....|....|....|....|....|....|....|....|....|....|....|....|....|....|....|
SFVcgu_916910     ATAGTACAAGCTCAATATAAGCCTGAATTTACTATAGTTAATCAATGGTCAATTCCTTTAGGTGACCATACAGCCCAGCTGGCTGAAGTTGCAGCTGTAG 5386
SFVpne_500057     ..T.CT....T.....T.C.A..C....A..AA..T..CC..........T..A..A..G..A.....C..T..T..AT.A......A.......A.... 5549
SFVtfr_083616     ..T.......TA....T.TTA..A....A..AAG.TA..C.A........T..C.....G..A..T........A..AT....A...A....T.....T. 5381

                        5810      5820      5830      5840      5850      5860      5870      5880      5890      5900
                  ....|....|....|....|....|....|....|....|....|....|....|....|....|....|....|....|....|....|....|....|
SFVcgu_916910     AATTTGCTTGTAAGAAGGCATTGAAAGTTTCAGGTCCTGTTTTAATAGTAACAGATAGTTTTTATGTTGCTGAAAGTGCTACCAAGGAGTTACCTTATTG 5486
SFVpne_500057     .......C.....A.....T..A...A.C..T.........C.C.....C..T..C...........A...........C.AT................. 5649
SFVtfr_083616     .............A.....T..A...A..CA...G..A..C........T..............................G....A..A...T.C..... 5481

                        5910      5920      5930      5940      5950      5960      5970      5980      5990      6000
                  ....|....|....|....|....|....|....|....|....|....|....|....|....|....|....|....|....|....|....|....|
SFVcgu_916910     GCAATCTAATGGTTTCCTTAATAATAAAAAGAAACCTTTGAAACATGTTTCTAAATGGAAATCAATAGCTGAGTGTTTACAGCTTAAACCCACCATAACC 5586
SFVpne_500057     .A.G.....C..C...A.......C..G..A......C.T...........C......................................TGAT..T..T 5749
SFVtfr_083616     .A..........G..TG......................A........A..............T.....A..A......A.TA.A.....AGAT..T..T 5581

                        6010      6020      6030      6040      6050      6060      6070      6080      6090      6100
                  ....|....|....|....|....|....|....|....|....|....|....|....|....|....|....|....|....|....|....|....|
SFVcgu_916910     ATTCTACATGAAAAAGGACACCAGTCTCCACAAACTTCTATTCATACAGAAGGTAATGCTTTAGCTGATAAGCTTGCCACCCAAGGGAGTTATGTAGTAC 5686
SFVpne_500057     ...A................T..ACAG..TATG...A.CT.A.....C.........AA....................................G..T. 5849
SFVtfr_083616     ...A....................C.ATTGTT...CAGC..A.....T.....A...A.......A....................A......A.G..CA 5681

                        6110      6120      6130      6140      6150      6160      6170      6180      6190      6200
                  ....|....|....|....|....|....|....|....|....|....|....|....|....|....|....|....|....|....|....|....|
SFVcgu_916910     ATACAAATACTACTCCAAGCCTGGATGCAGAATTGGATCAATTACTACAAGGACTACATCCTAAAGGATATCCAAAACAATATATTTACCAATTGGAAAA 5786
SFVpne_500057     ..TGT.....C....................GC.......G..G..........ATA.....CC...G..C....C......C.AA..TACCC.T..GG. 5949
SFVtfr_083616     ..TGTGT..GA.AA.....T...........GC........G.TA..A.G..TAA.T.......G........T.........CAG...TT.......G. 5781

                        6210      6220      6230      6240      6250      6260      6270      6280      6290      6300
                  ....|....|....|....|....|....|....|....|....|....|....|....|....|....|....|....|....|....|....|....|
SFVcgu_916910     TGGCCAAGTCACTGTCATAAGGCCAGCTGGTAAAAGAATTATACCTCCATCAGCTGATCGACAGTCTATAATTTTATCAGCCCACAATATTGCTCATACA 5886
SFVpne_500057     ..ATA..A.T.TA..TGA......TA.C..A.G.C.G..AG.G......AA.......A..G.AAAA..T..C.CTA..........C............ 6049
SFVtfr_083616     A..A.TT..T.AA..A..T.....TCAA..A.TT.....A..T.......T.TTA..CA..GCTAAC......C..AA...T..T..A...........T 5881

                        6310      6320      6330      6340      6350      6360      6370      6380      6390      6400
                  ....|....|....|....|....|....|....|....|....|....|....|....|....|....|....|....|....|....|....|....|
SFVcgu_916910     GGAAGAGATGCCACCTTCTTAAAAGTTACTTCTCAGTATTGGTGGCCTAATCTTAGAAAGGATGTGGTTAAAGTCATCAGACAATGTAAACAATGTCTGG 5986
SFVpne_500057     ...C....C..T..T.....G......T....CA...................A....................G........G....C...G....... 6149
SFVtfr_083616     ..T........T..A..T..G..G..CT...................A...........A.................A.....G...C........T.A. 5981

                        6410      6420      6430      6440      6450      6460      6470      6480      6490      6500
                  ....|....|....|....|....|....|....|....|....|....|....|....|....|....|....|....|....|....|....|....|
SFVcgu_916910     TAACTAATTCTACAGCCCTGTCAGCTCCCCCAATTTTGAGGCCTGACAGACCCTTAAAACCTTTTGATAAATTTTATATTGATTATATTGGCCCCTTGCC 6086
```

```
SFVpne_500057  .C..A...G..G..AAT..AA.TT.G..T.....AC.T........G.AG..TC.C..G..C.................C...........A..T..A.. 6249
SFVtfr_083616 .C..C...G.GG.TAA.T.AA.TT.CT.TT.......A........G..G..TCA...G...........G....TC.......T......A..TC.A.. 6081

                    6510      6520      6530      6540      6550      6560      6570      6580      6590      6600
               ....|....|....|....|....|....|....|....|....|....|....|....|....|....|....|....|....|....|....|....|
SFVcgu_916910  TCCTTCTAATGGCTATTTACATGTCCTTGTAGTAGTCGATGGTATGACTGGATTTGTATGGTTATACCCCACTAAGGCTCCCACAACAAGCGCAACTGTT 6186
SFVpne_500057  A...........T....................G.................C....................A.........T....T............ 6349
SFVtfr_083616 A...........A...C.......TT.A..G..G..A...A..........G...TGT..............A.....C..T..T..C.A.......... 6181

                    6610      6620      6630      6640      6650      6660      6670      6680      6690      6700
               ....|....|....|....|....|....|....|....|....|....|....|....|....|....|....|....|....|....|....|....|
SFVcgu_916910  AAAGCTCTCAACATGCTCACTAGTATTGCAATTCCTAAGGTGTTGCATTCTGATCAGGGTGCAGCATTCACCTCTTCCACTTTTGCTGATTGGGCAAAAG 6286
SFVpne_500057  ...........T.......................A......C....C........A..G..G..............A.................T.... 6449
SFVtfr_083616 ...........T.......................A......C.............A..AT...................C........A........G. 6281

                    6710      6720      6730      6740      6750      6760      6770      6780      6790      6800
               ....|....|....|....|....|....|....|....|....|....|....|....|....|....|....|....|....|....|....|....|
SFVcgu_916910  ATAAAGGTATACAATTGGAATTCAGTACTCCTTACCATCCCCAAAGTAGTGGTAAGGTGGAAAGGAAAAATAGTGACATTAAACGACTTTTAACTAAACT 6386
SFVpne_500057  .A..................................................C..........................A.................... 6549
SFVtfr_083616 .A................................T..C........C.............................A..A.................... 6381

                    6810      6820      6830      6840      6850      6860      6870      6880      6890      6900
               ....|....|....|....|....|....|....|....|....|....|....|....|....|....|....|....|....|....|....|....|
SFVcgu_916910  GCTTATTGGGAGACCTGCTAAGTGGTATGATCTTCTTCCAGTCGTACAATTGGCATTAAATAACTCTTATAGTCCATCTTCTAAATATACTCCTCATCAA 6486
SFVpne_500057  ...A.............................G..G..T..T....................T.....C...............C.............. 6649
SFVtfr_083616 .T.GG.A..A......A.A...........C...T.G..C...........A...........T..A........T..C..C...AG...C........G 6481

                    6910      6920      6930      6940      6950      6960      6970      6980      6990      7000
               ....|....|....|....|....|....|....|....|....|....|....|....|....|....|....|....|....|....|....|....|
SFVcgu_916910  CTTTTATTTGGCATAGATTCAAATACTCCATTTGCAAATTCTGATACACTTGACTTATCTCGAGAAGAAGAACTATCTTTGTTACAGGAAATTAGATCTT 6586
SFVpne_500057  ..C..G.....TG.......C..C..A..G.......................T.....CA.......G..GT.G...C.T...........A....... 6749
SFVtfr_083616 T.G..G...........C......GT...T.........AAG..C........T......A.............TG..C.T.....A..G..C...A... 6581

                    7010      7020      7030      7040      7050      7060   3'PPT 7080      7090      7100
               ....|....|....|....|....|....|....|....|....|....|....|....|....|....|....|....|....|....|....|....|
SFVcgu_916910  CACTTGTGCCTCCAGCCGCCCCTCCACCATCCTCTCGTTCCTGGGTTCCTCTTGTTGGTCAACTGGTCCAGGAGAGGGTGGCTCGGCCTGCTTCACTAAG 6686
SFVpne_500057  .CT.GTAC.AG...A..T....G..TG.C.....G.........TCA...TC......C...T................A..A..T............C. 6849
SFVtfr_083616 .T...CAC.AG..TA..T....A...T.C....TA..C......A.....G....C..C........................A........A....TC. 6681

                    7110      7120      7130      7140      7150      7160      7170      7180      7190      7200
               ....|....|....|....|....|....|....|....|....|....|....|....|....|....|....|....|....|....|....|....|
SFVcgu_916910  ACCACGGTGGCATAAACCTACTCCAATCACAGAAGTTCTCAATCCAAGAACTGTAATAATTGTGGACCATCTTGGCAACAAGAGAACTGTAAGTGTTGAT 6786
SFVpne_500057  ......T........G......A.C..TCTT..G..CG.A..C..TC.G..G..GG.C...T...................AC................C 6949
SFVtfr_083616 ...T..T............GT.AAGG.ATTG......T.G......C.......GG.C...T.......CT.G........C..G.T...G.....G..C 6781

                    7210      7220      7230  Env 7240      7250      7260      7270     Pol        7290      7300
               ....|....|....|....|....|....|....|....|....|....|....|....|....|....|....|....|....|....|....|....|
SFVcgu_916910  AATCTTAAATTAACAGCTCATCAGGACAAACAGCATGGCACCACCAATGACGCTTCAACAATGGATGCAATGGAAAAAGATGAATGATCTTCATCAGGCT 6886
```

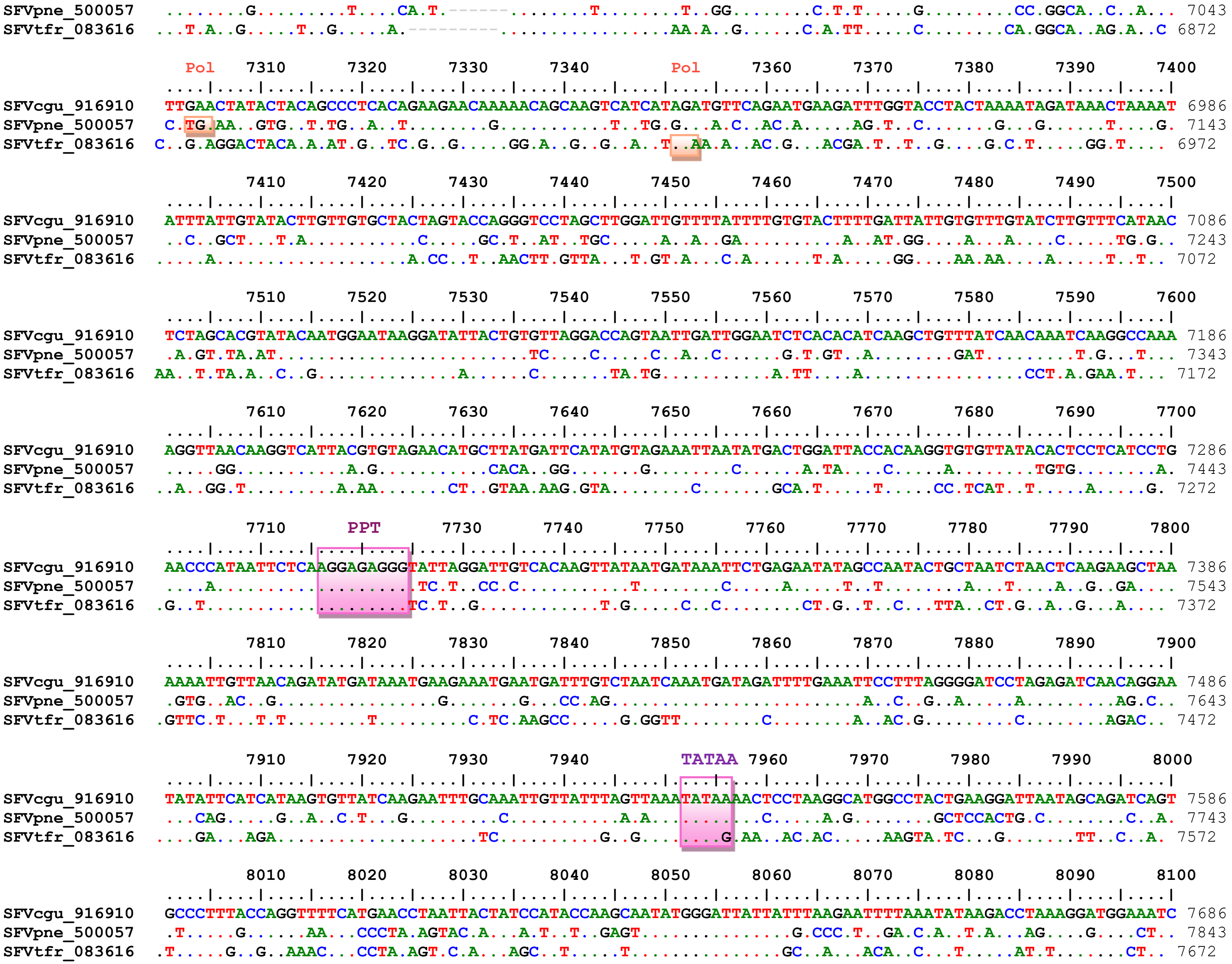

SFVpne_500057
SFVtfr_083616
SFVcgu_916910
Pol
PPT
TATAA
7043
6872
6986
7143
6972
7086
7243
7072
7186
7343
7172
7286
7443
7272
7386
7543
7372
7486
7643
7472
7586
7743
7572
7686
7843
7672

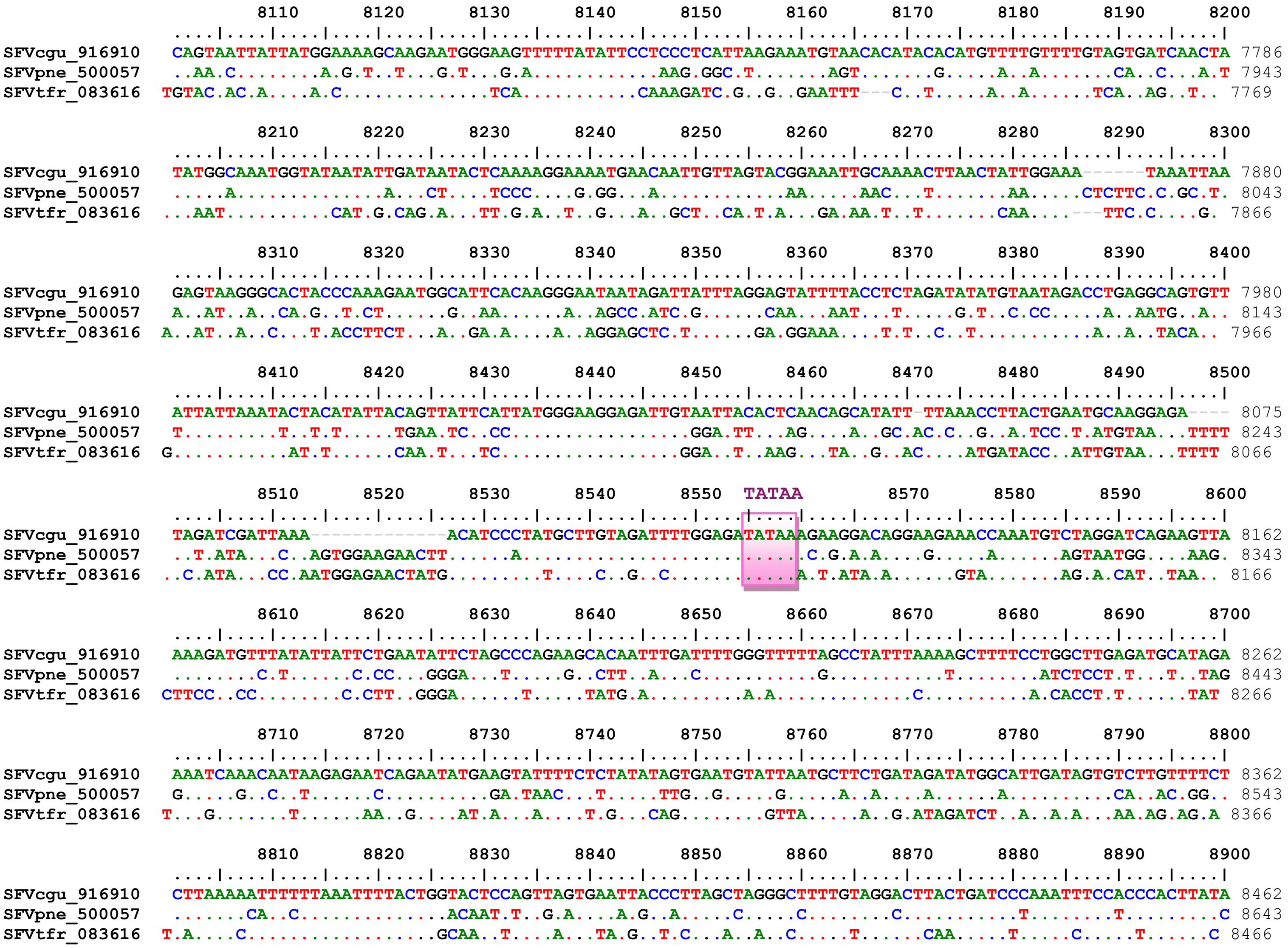
SFVcgu_916910
SFVpne_500057
SFVtfr_083616
TATAA

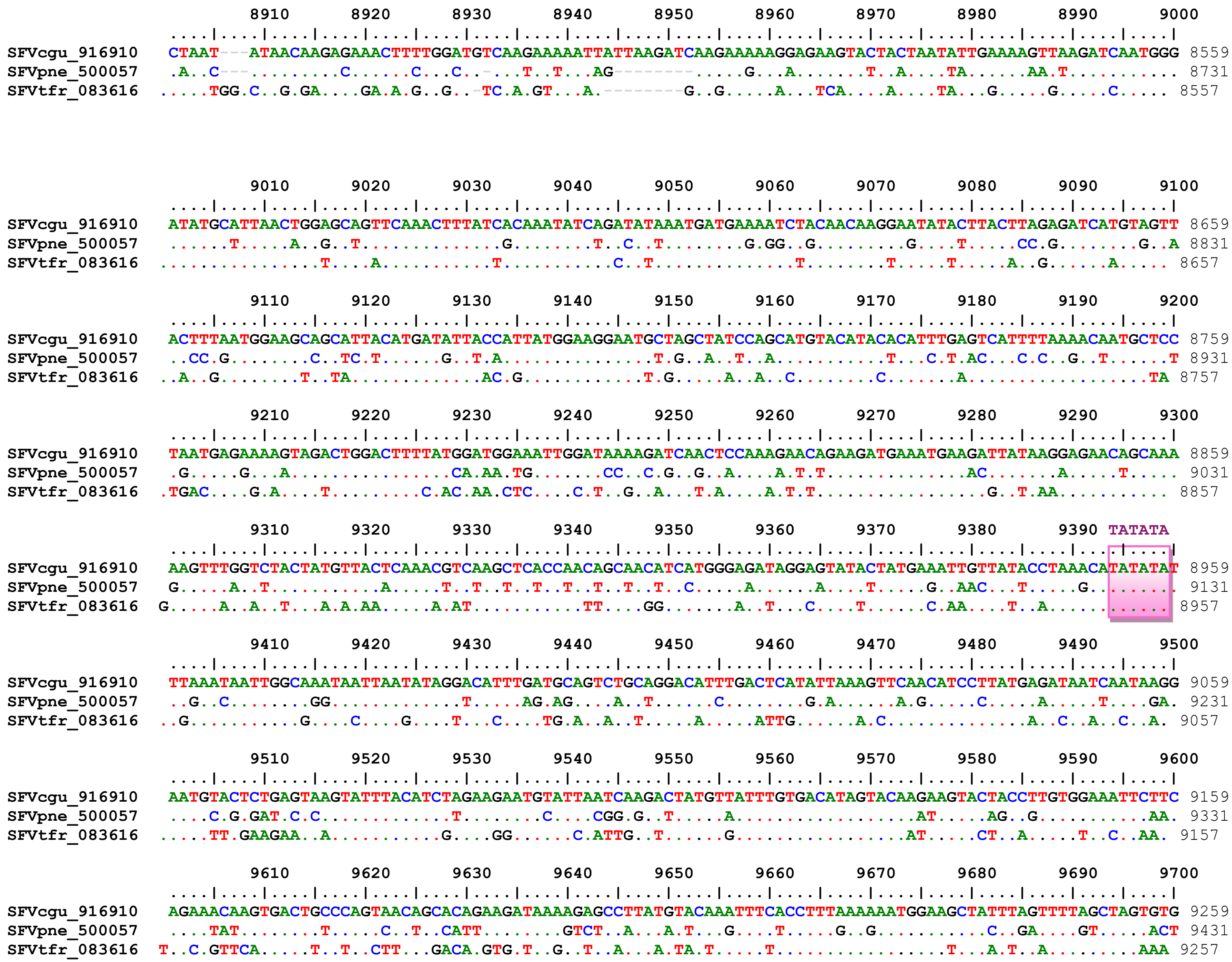
SFVcgu_916910
SFVpne_500057
SFVtfr_083616
TATATA

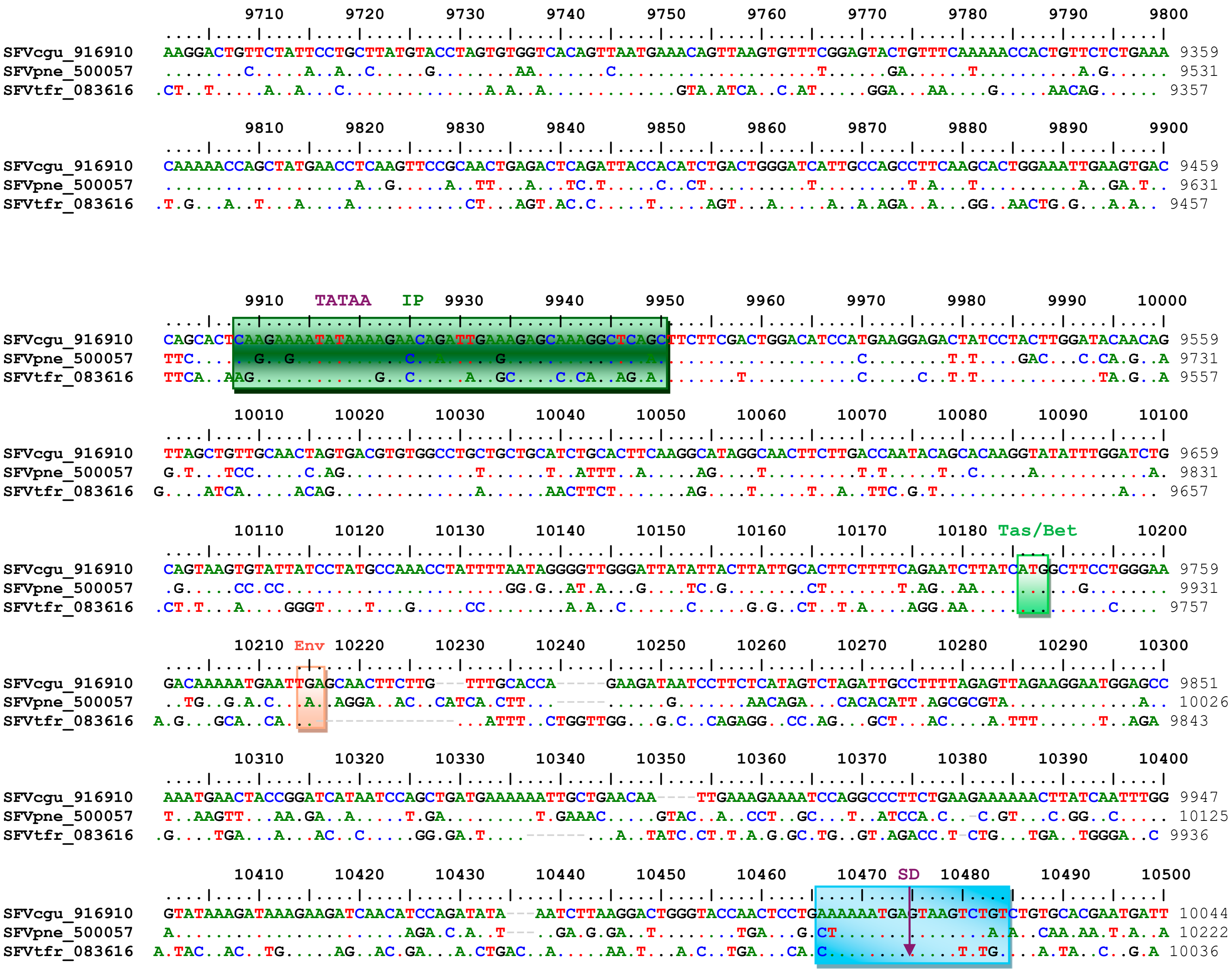

SFVcgu_916910
SFVpne_500057
SFVtfr_083616
TATAA
IP
Tas/Bet
Env
SD

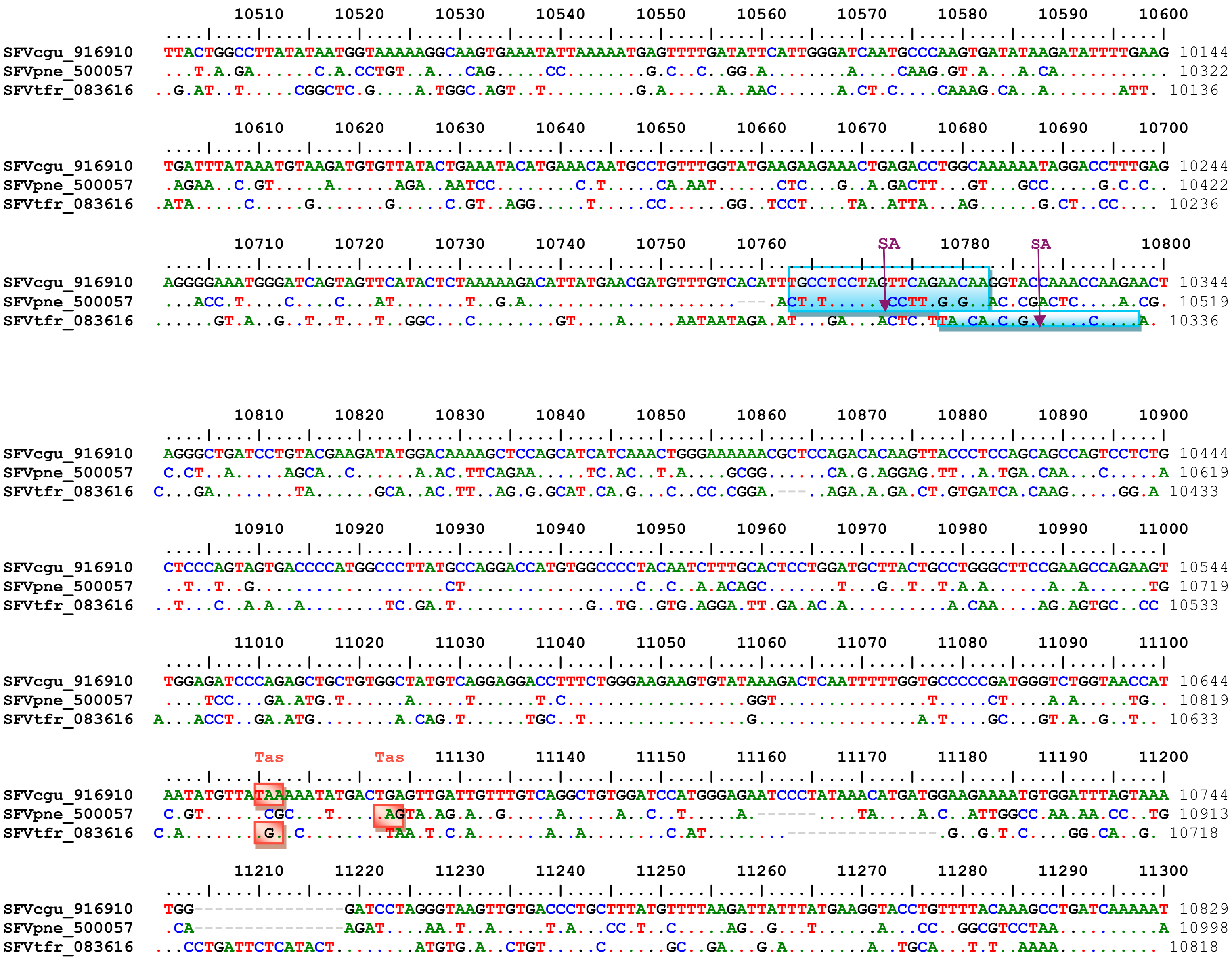
SFVcgu_916910
SFVpne_500057
SFVtfr_083616
SA
SA
Tas
Tas

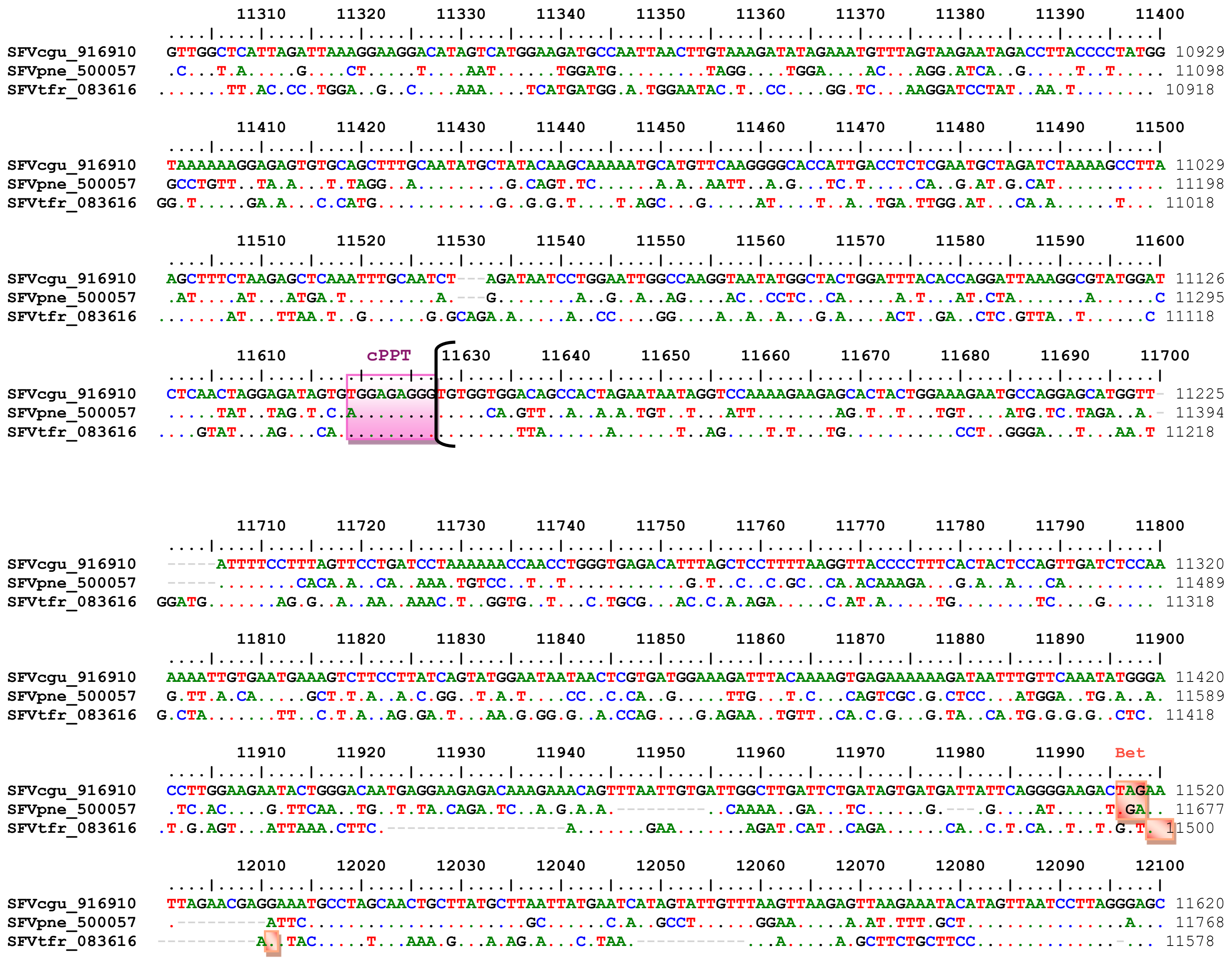
SFVcgu_916910
SFVpne_500057
SFVtfr_083616
cPPT
Bet

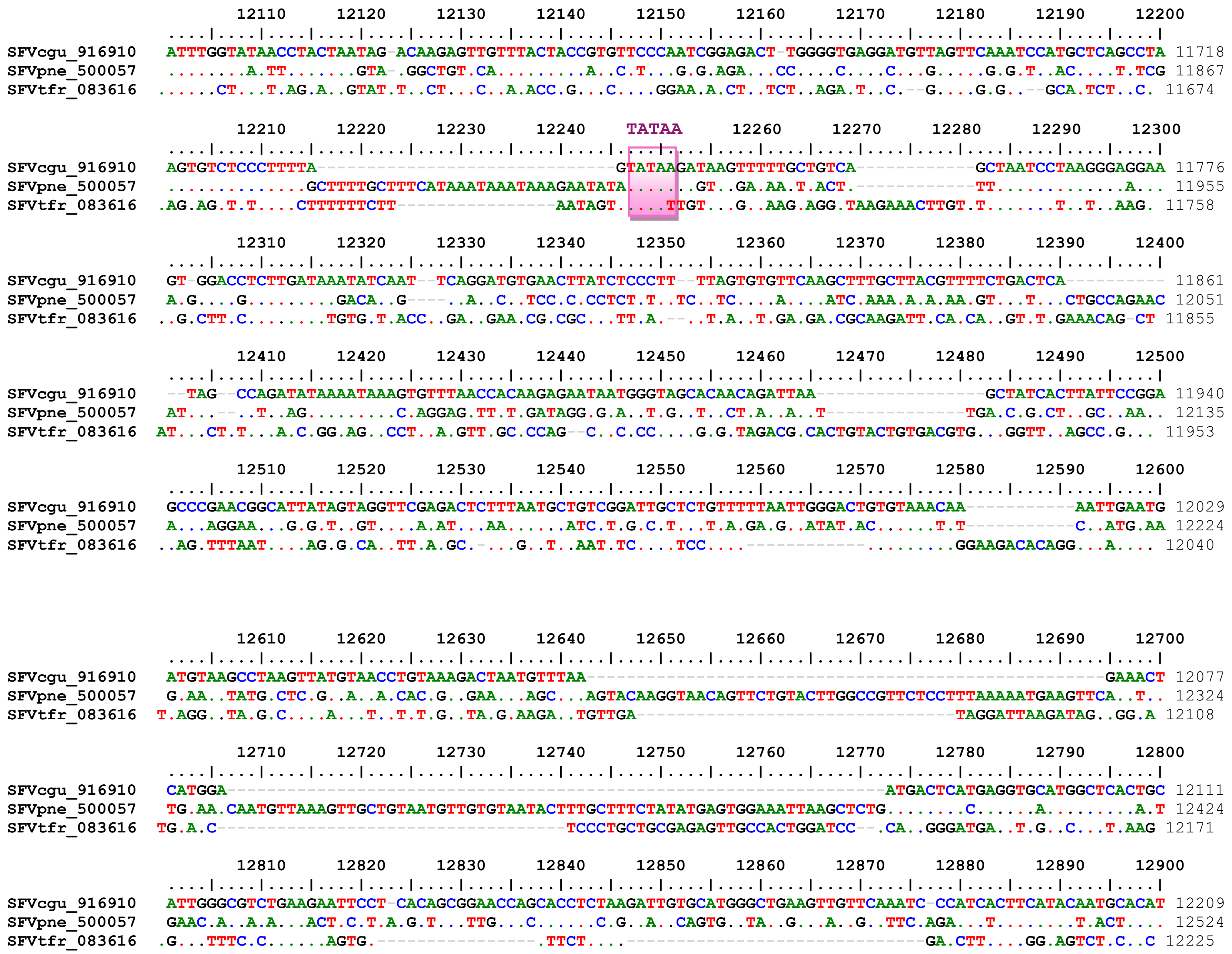

TATAA

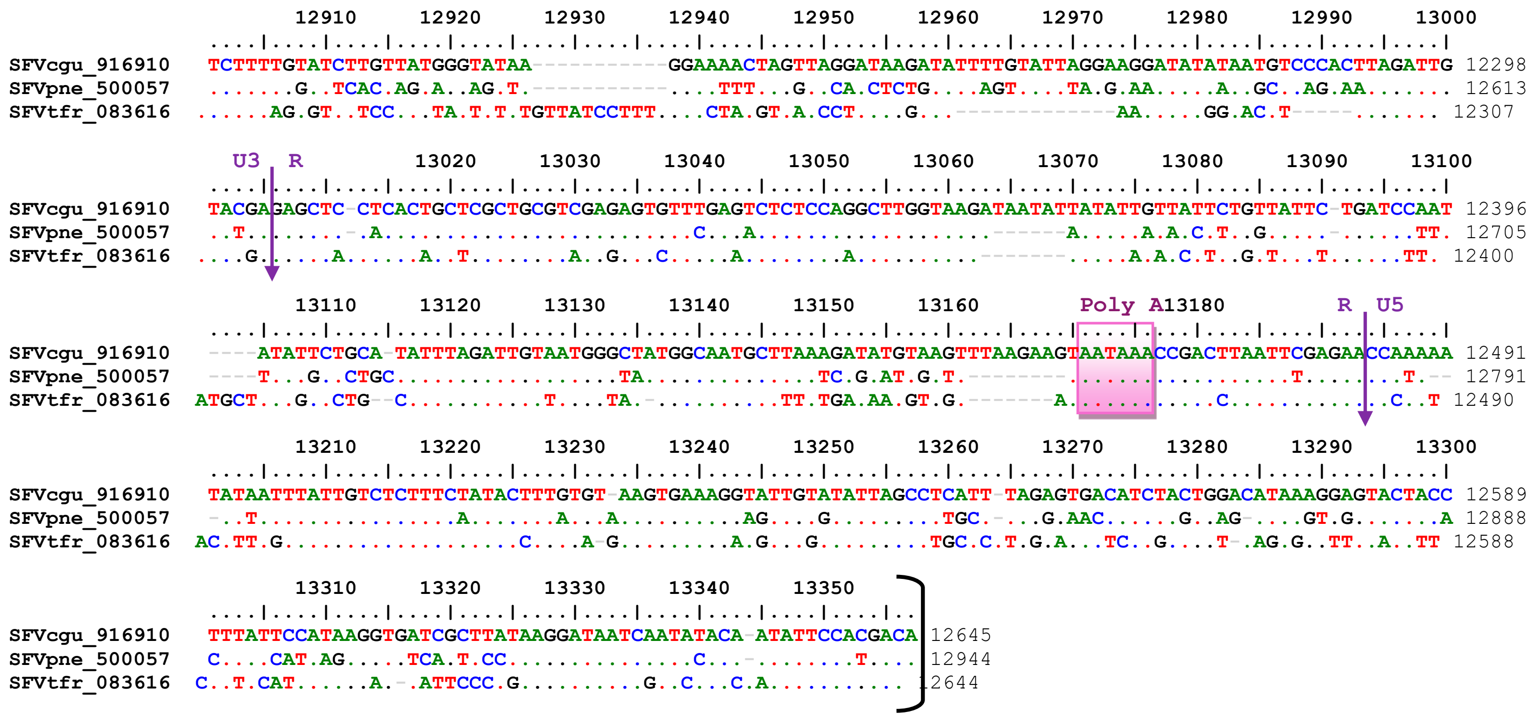

SFVcgu_916910
SFVpne_500057
SFVtfr_083616
U3
R
Poly A
R
U5
12298
12613
12307
12396
12705
12400
12491
12791
12490
12589
12888
12588
12645
12944
12644

**Figure S2**

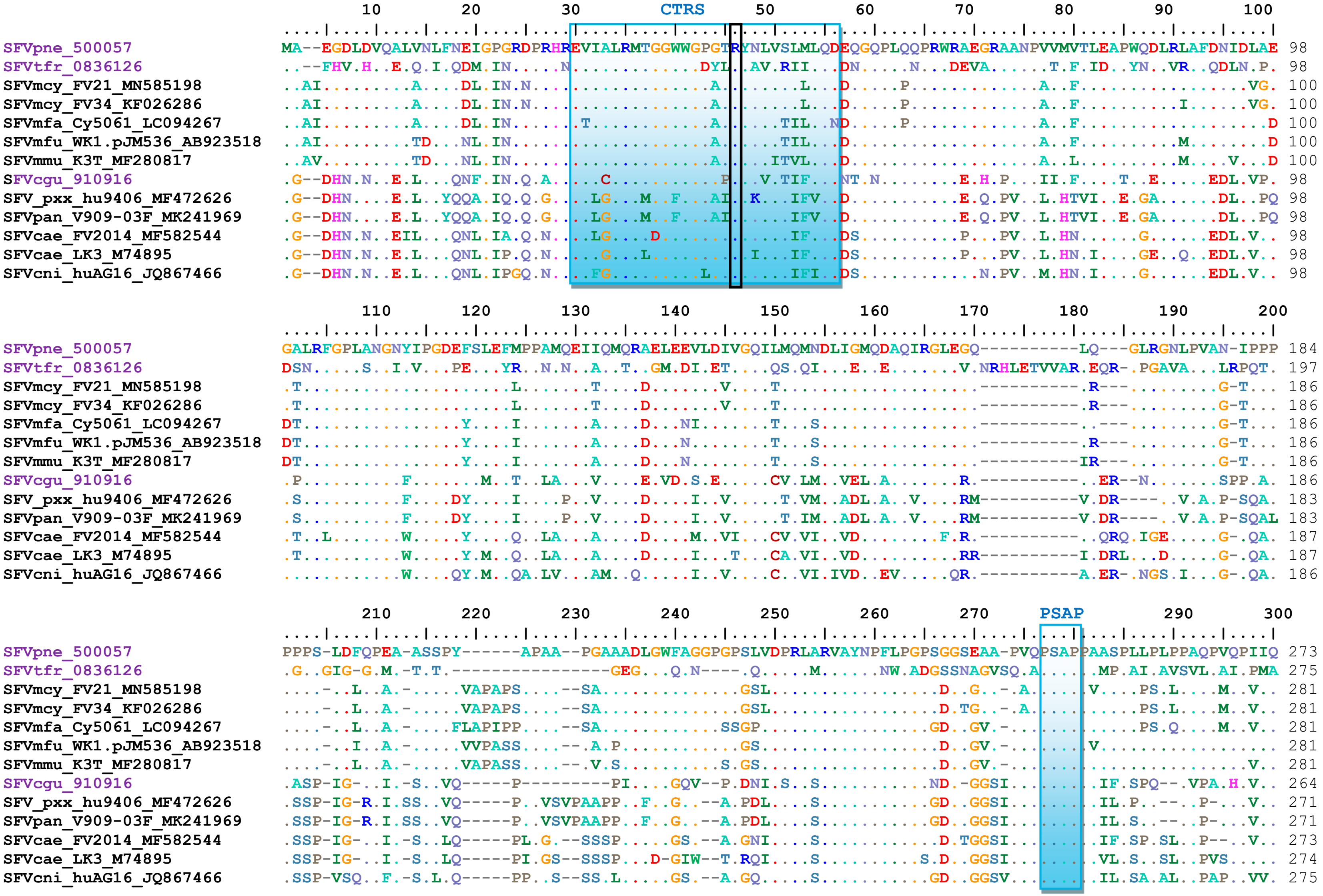

CTRS
PSAP
SFVpne_500057
SFVtfr_0836126
SFVmcy_FV21_MN585198
SFVmcy_FV34_KF026286
SFVmfa_Cy5061_LC094267
SFVmfu_WK1.pJM536_AB923518
SFVmmu_K3T_MF280817
SFVcgu_910916
SFV_pxx_hu9406_MF472626
SFVpan_V909-03F_MK241969
SFVcae_FV2014_MF582544
SFVcae_LK3_M74895
SFVcni_huAG16_JQ867466

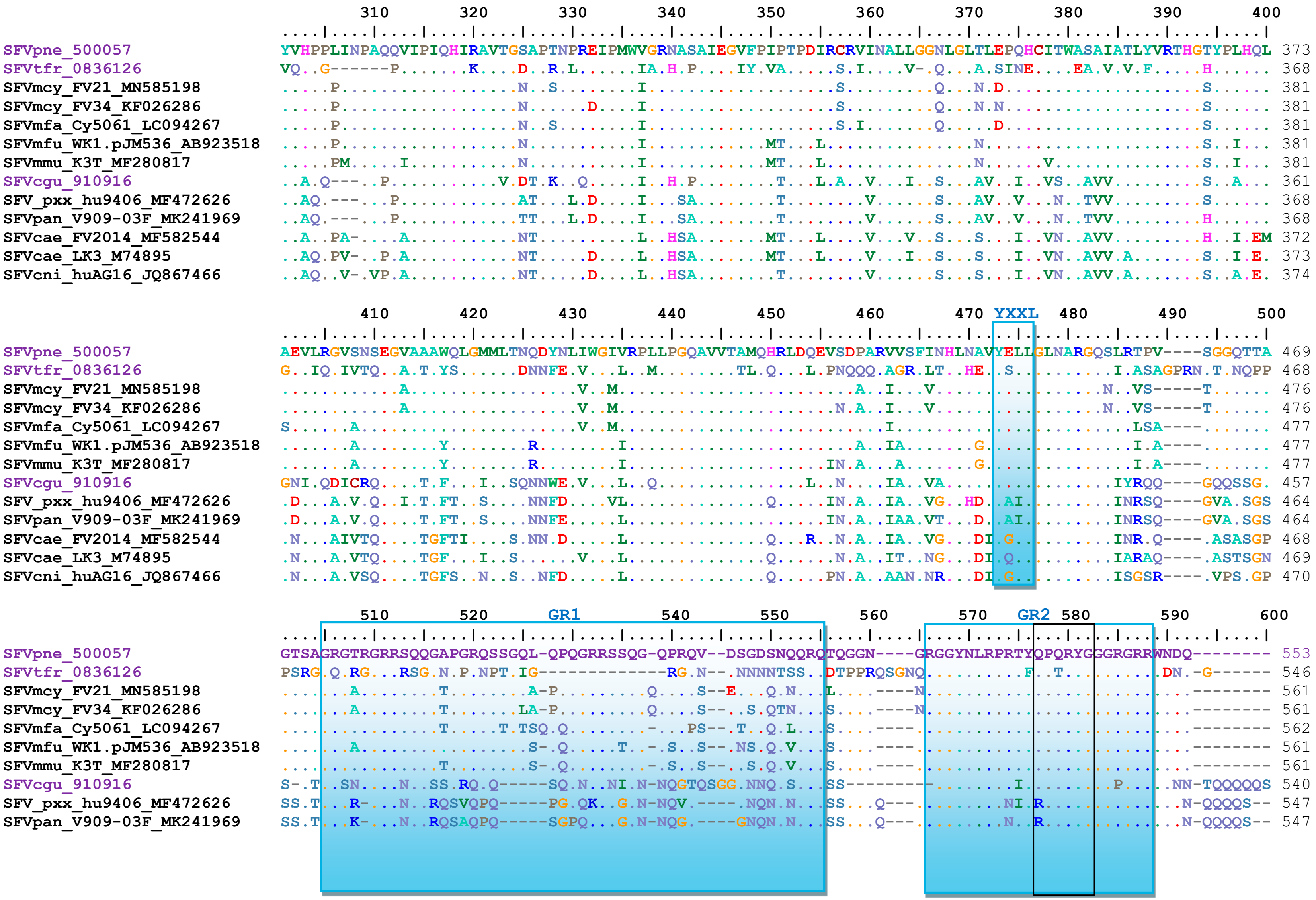
YXXL
GR1
GR2
SFVpne_500057
SFVtfr_0836126
SFVmcy_FV21_MN585198
SFVmcy_FV34_KF026286
SFVmfa_Cy5061_LC094267
SFVmfu_WK1.pJM536_AB923518
SFVmmu_K3T_MF280817
SFVcgu_910916
SFV_pxx_hu9406_MF472626
SFVpan_V909-03F_MK241969
SFVcae_FV2014_MF582544
SFVcae_LK3_M74895
SFVcni_huAG16_JQ867466

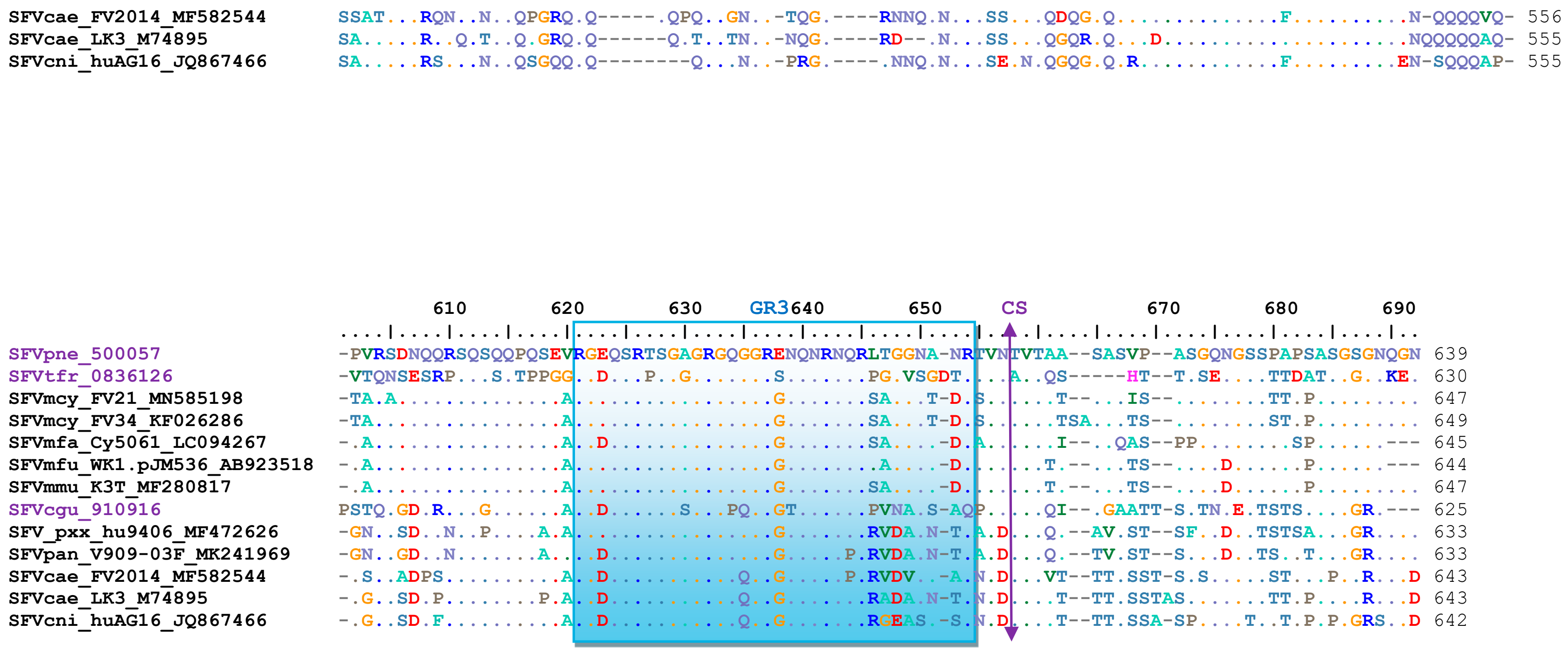
SFVcae_FV2014_MF582544
SFVcae_LK3_M74895
SFVcni_huAG16_JQ867466
610
620
630
GR3
640
650
CS
670
680
690
SFVpne_500057
SFVtfr_0836126
SFVmcy_FV21_MN585198
SFVmcy_FV34_KF026286
SFVmfa_Cy5061_LC094267
SFVmfu_WK1.pJM536_AB923518
SFVmmu_K3T_MF280817
SFVcgu_910916
SFV_pxx_hu9406_MF472626
SFVpan_V909-03F_MK241969
SFVcae_FV2014_MF582544
SFVcae_LK3_M74895
SFVcni_huAG16_JQ867466

Figure S3

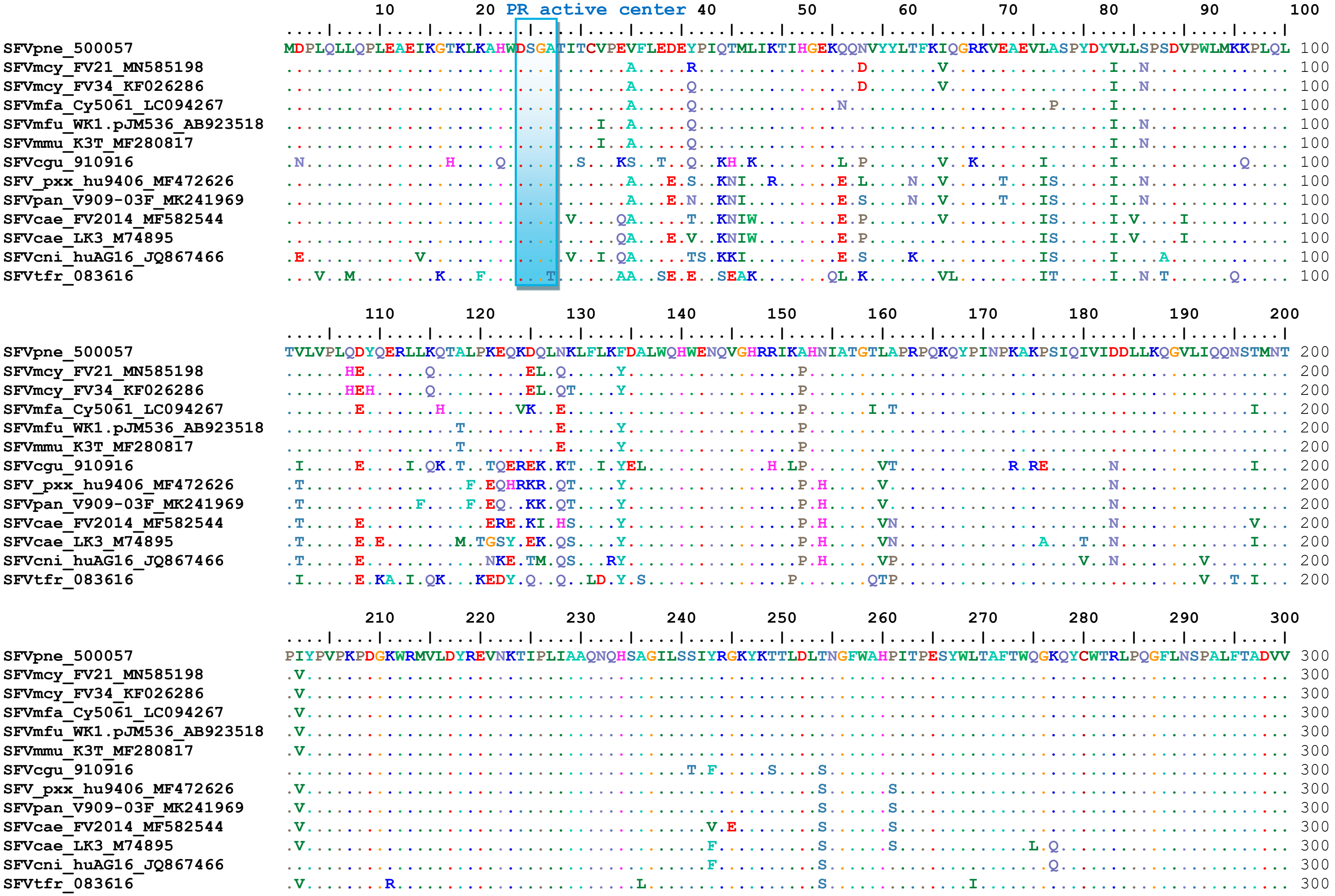
PR active center
SFVpne_500057
SFVmcy_FV21_MN585198
SFVmcy_FV34_KF026286
SFVmfa_Cy5061_LC094267
SFVmfu_WK1.pJM536_AB923518
SFVmmu_K3T_MF280817
SFVcgu_910916
SFV_pxx_hu9406_MF472626
SFVpan_V909-03F_MK241969
SFVcae_FV2014_MF582544
SFVcae_LK3_M74895
SFVcni_huAG16_JQ867466
SFVtfr_083616

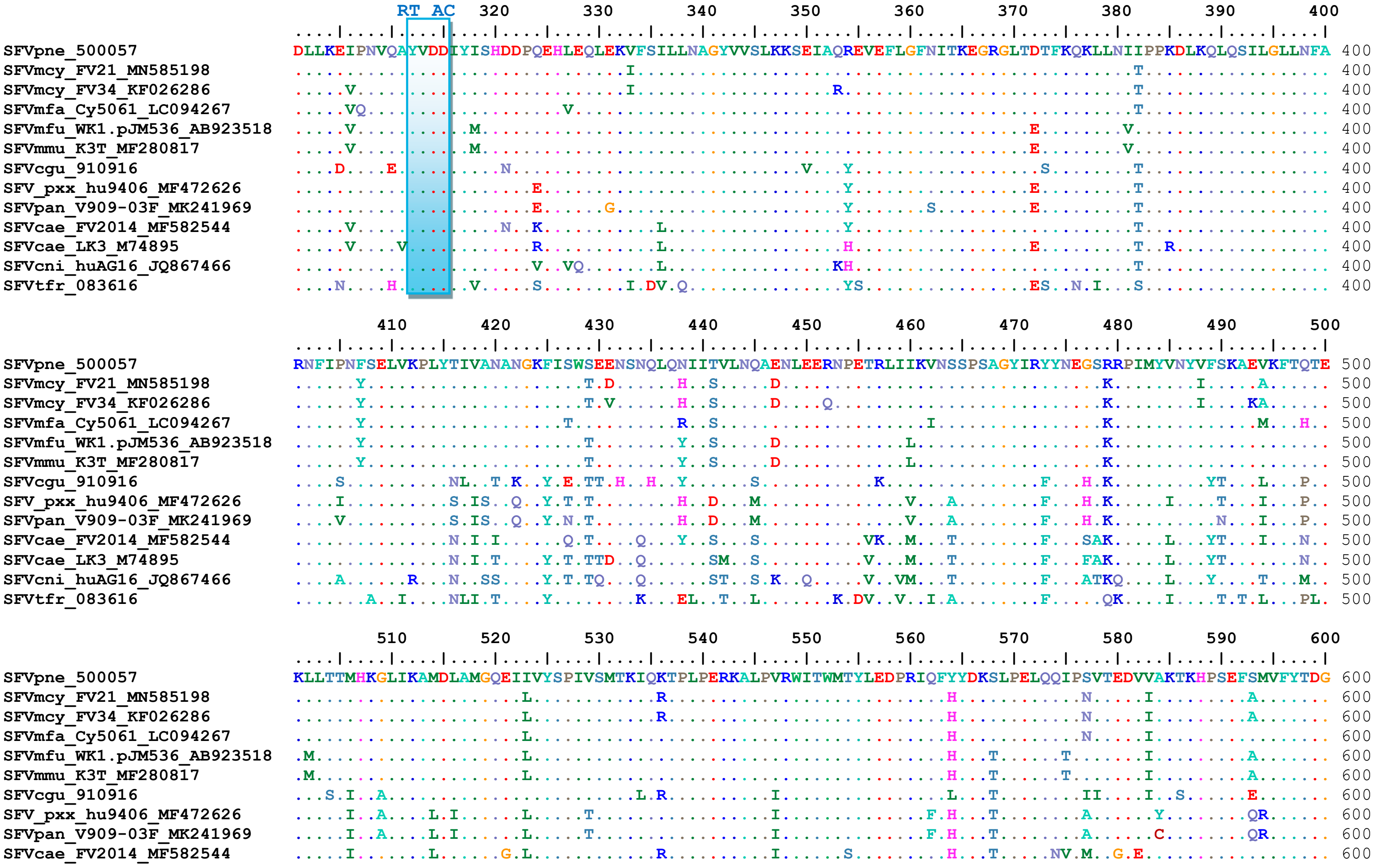
RT AC
SFVpne_500057
SFVmcy_FV21_MN585198
SFVmcy_FV34_KF026286
SFVmfa_Cy5061_LC094267
SFVmfu_WK1.pJM536_AB923518
SFVmmu_K3T_MF280817
SFVcgu_910916
SFV_pxx_hu9406_MF472626
SFVpan_V909-03F_MK241969
SFVcae_FV2014_MF582544
SFVcae_LK3_M74895
SFVcni_huAG16_JQ867466
SFVtfr_083616

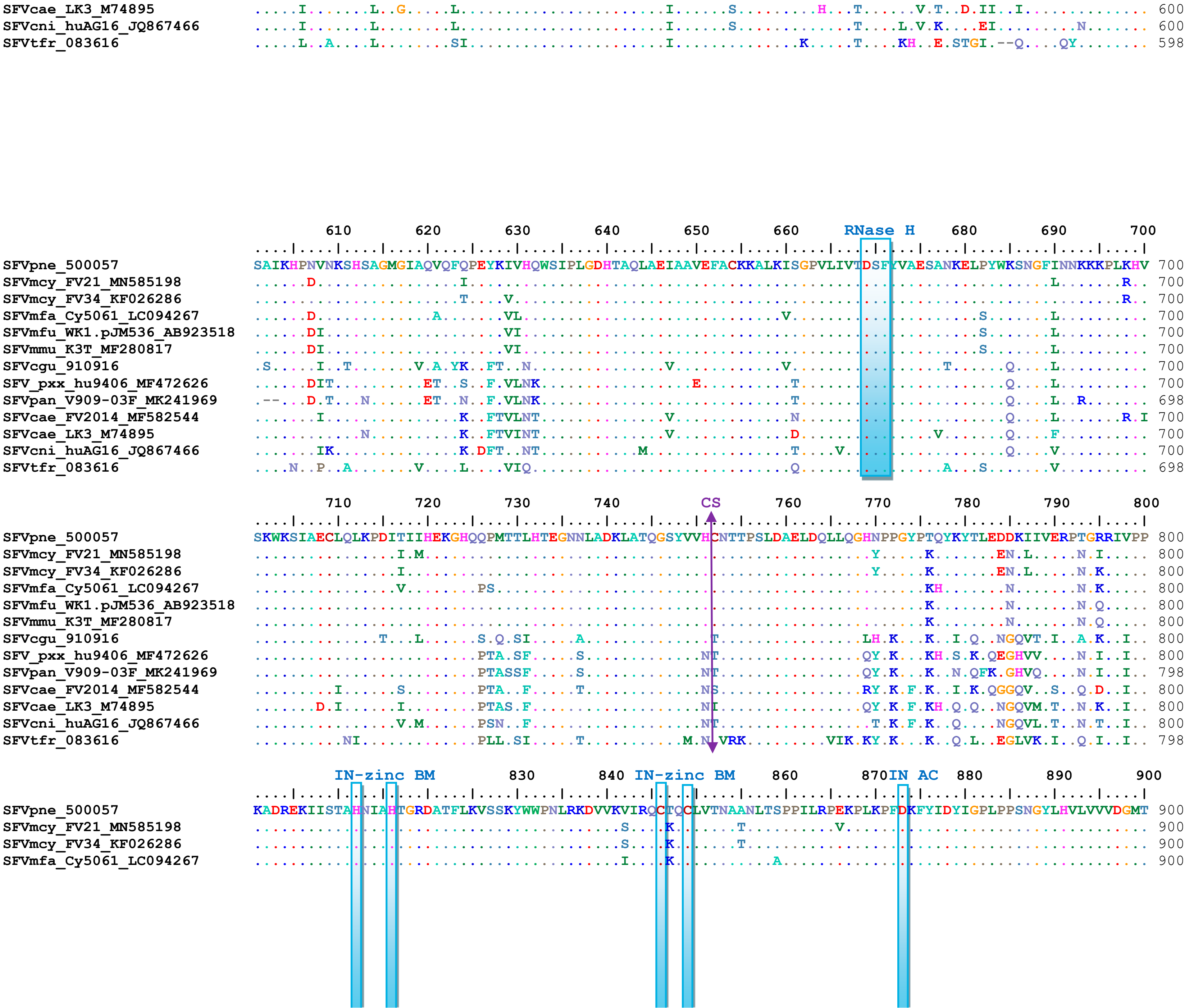

SFVcae_LK3_M74895
SFVcni_huAG16_JQ867466
SFVtfr_083616
600
600
598
610 620 630 640 650 660 RNase H 680 690 700
SFVpne_500057
SAIKHPNVNKSHSAGMGIAQVQFQPEYKIVHQWSIPLGDHTAQLAEIAAVEFACKKALKISGPVLIVTDSFYVAESANKELPYWKSNGFINNKKKPLKHV 700
SFVmcy_FV21_MN585198
SFVmcy_FV34_KF026286
SFVmfa_Cy5061_LC094267
SFVmfu_WK1.pJM536_AB923518
SFVmmu_K3T_MF280817
SFVcgu_910916
SFV_pxx_hu9406_MF472626
SFVpan_V909-03F_MK241969
SFVcae_FV2014_MF582544
SFVcae_LK3_M74895
SFVcni_huAG16_JQ867466
SFVtfr_083616
710 720 730 740 CS 760 770 780 790 800
SKWKSIAECLQLKPDITIIHEKGHQQPMTTLHTEGNNLADKLATQGSYVVHCNTTPSLDAELDQLLQGHNPPGYPTQYKYTLEDDKIIVERPTGRRIVPP 800
IN-zinc BM 830 840 IN-zinc BM 860 870 IN AC 880 890 900
KADREKIISTAHNIAHTGRDATFLKVSSKYWWPNLRKDVVKVIRQCTQCLVTNAANLTSPPILRPEKPLKPFDKFYIDYIGPLPPSNGYLHVLVVVDGMT 900

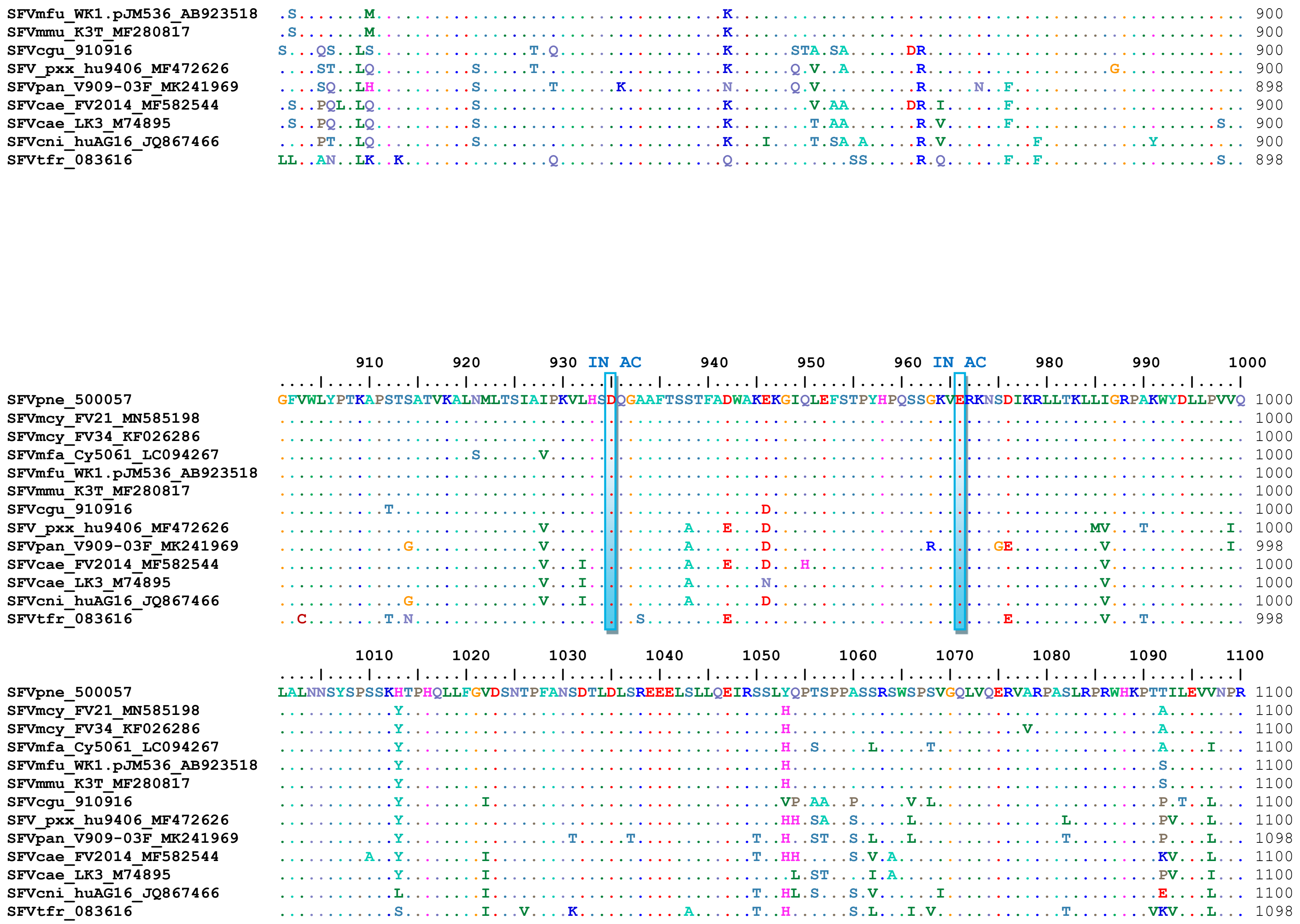
SFVmfu_WK1.pJM536_AB923518
SFVmmu_K3T_MF280817
SFVcgu_910916
SFV_pxx_hu9406_MF472626
SFVpan_V909-03F_MK241969
SFVcae_FV2014_MF582544
SFVcae_LK3_M74895
SFVcni_huAG16_JQ867466
SFVtfr_083616
910 920 930 IN AC 940 950 960 IN AC 980 990 1000
SFVpne_500057
GFVWLYPTKAPSTSATVKALNMLTSIAIPKVLHSDQGAAFTSSTFADWAKEKGIQLEFSTPYHPQSSGKVERKNSDIKRLLTKLLIGRPAKWYDLLPVVQ 1000
SFVmcy_FV21_MN585198
SFVmcy_FV34_KF026286
SFVmfa_Cy5061_LC094267
SFVmfu_WK1.pJM536_AB923518
SFVmmu_K3T_MF280817
SFVcgu_910916
SFV_pxx_hu9406_MF472626
SFVpan_V909-03F_MK241969
SFVcae_FV2014_MF582544
SFVcae_LK3_M74895
SFVcni_huAG16_JQ867466
SFVtfr_083616
1010 1020 1030 1040 1050 1060 1070 1080 1090 1100
SFVpne_500057
LALNNSYSPSSKHTPHQLLFGVDSNTPFANSDTLDLSREEELSLLQEIRSSLYQPTSPPASSRSWSPSVGQLVQERVARPASLRPRWHKPTTILEVVNPR 1100
SFVmcy_FV21_MN585198
SFVmcy_FV34_KF026286
SFVmfa_Cy5061_LC094267
SFVmfu_WK1.pJM536_AB923518
SFVmmu_K3T_MF280817
SFVcgu_910916
SFV_pxx_hu9406_MF472626
SFVpan_V909-03F_MK241969
SFVcae_FV2014_MF582544
SFVcae_LK3_M74895
SFVcni_huAG16_JQ867466
SFVtfr_083616

```
                                       1110      1120      1130      1140      1150      1160
                            ....|....|....|....|....|....|....|....|....|....|....|....|....|..
SFVpne_500057               TVVILDHLGNKRTVSVDNLKLTAYHN--NGTSNDSGTMASMEKDEPGTPSS---------------- 1149
SFVmcy_FV21_MN585198        ..I.......R.............QD--...........L..E..SS.S.T---------------- 1149
SFVmcy_FV34_KF026286        ..I.......R.............Q.--......FR...LV.E...S.S.T---------------- 1149
SFVmfa_Cy5061_LC094267      ..........R............HQ---...T..F..V.PV.E...SAS.P---------------- 1148
SFVmfu_WK1.pJM536_AB923518  ..........R............HQ.--...T.......PV.E..S.S...---------------- 1149
SFVmmu_K3T_MF280817         ..........R............HQ.--...T.......PV.E..S.S...---------------- 1149
SFVcgu_910916               ..I.V..................HQDKQH..T..AS..DA.....---------------------- 1145
SFV_pxx_hu9406_MF472626     ......................V.QDS-..S....AA..P..E..---------------------- 1144
SFVpan_V909-03F_MK241969    ..I.....................QQQED..T...T...LV.E..---------------------- 1143
SFVcae_FV2014_MF582544      ..........R...........V.Q.--..S..E.SAV.A..EN.---------------------- 1143
SFVcae_LK3_M74895           A.........R.............QK--D..P.E.AAVVA.....---------------------- 1143
SFVcni_huAG16_JQ867466      ..........R.............QE--H..T.EPAA..PL.EN.---------------------- 1143
SFVtfr_083616               ..........N.I..........HQ---D..T..NTA..TL.Q..Q..R.PGRTTNIDSGAKGTGDN 1162
```

Figure S4

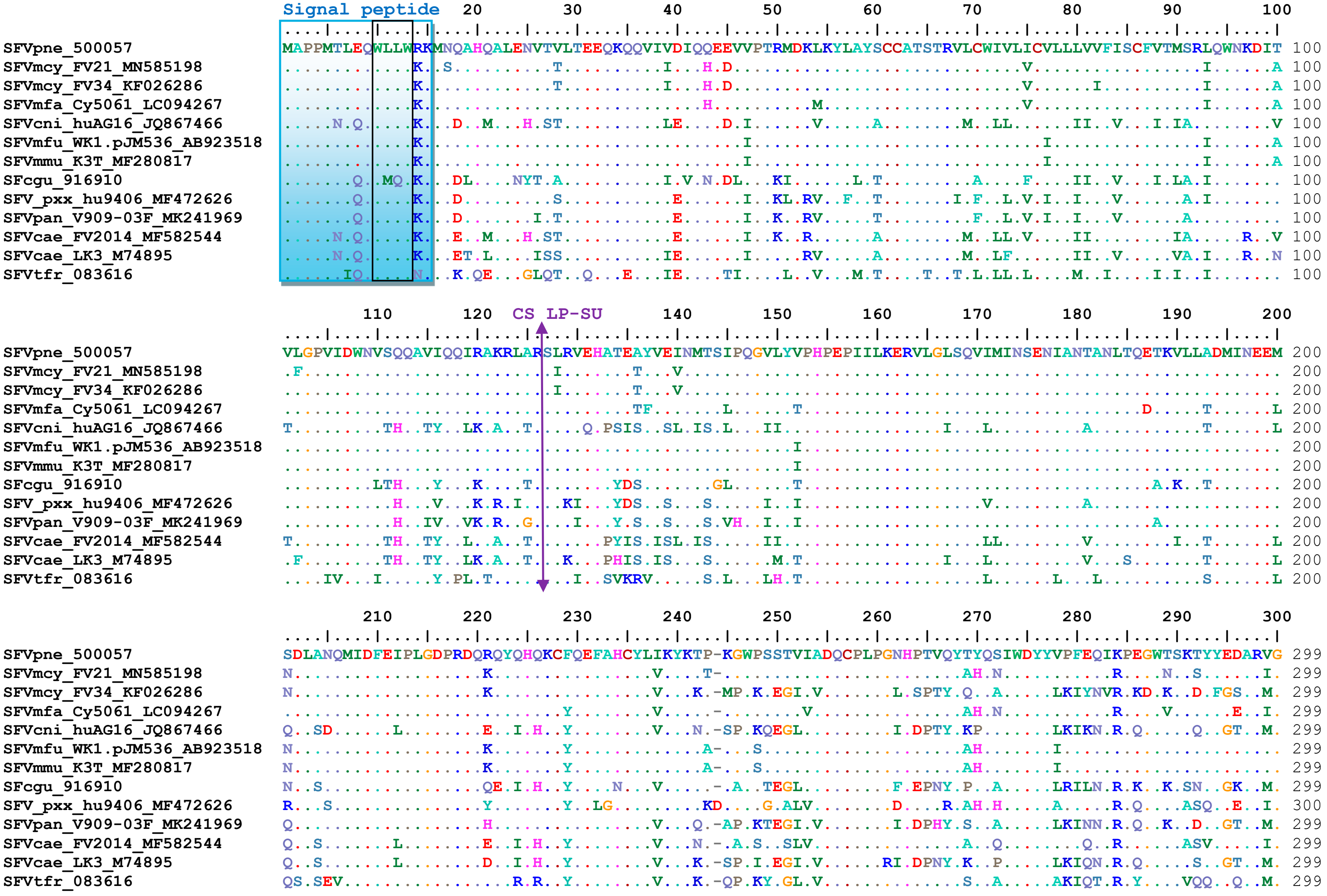
Signal peptide
CS
LP-SU
SFVpne_500057
SFVmcy_FV21_MN585198
SFVmcy_FV34_KF026286
SFVmfa_Cy5061_LC094267
SFVcni_huAG16_JQ867466
SFVmfu_WK1.pJM536_AB923518
SFVmmu_K3T_MF280817
SFcgu_916910
SFV_pxx_hu9406_MF472626
SFVpan_V909-03F_MK241969
SFVcae_FV2014_MF582544
SFVcae_LK3_M74895
SFVtfr_083616

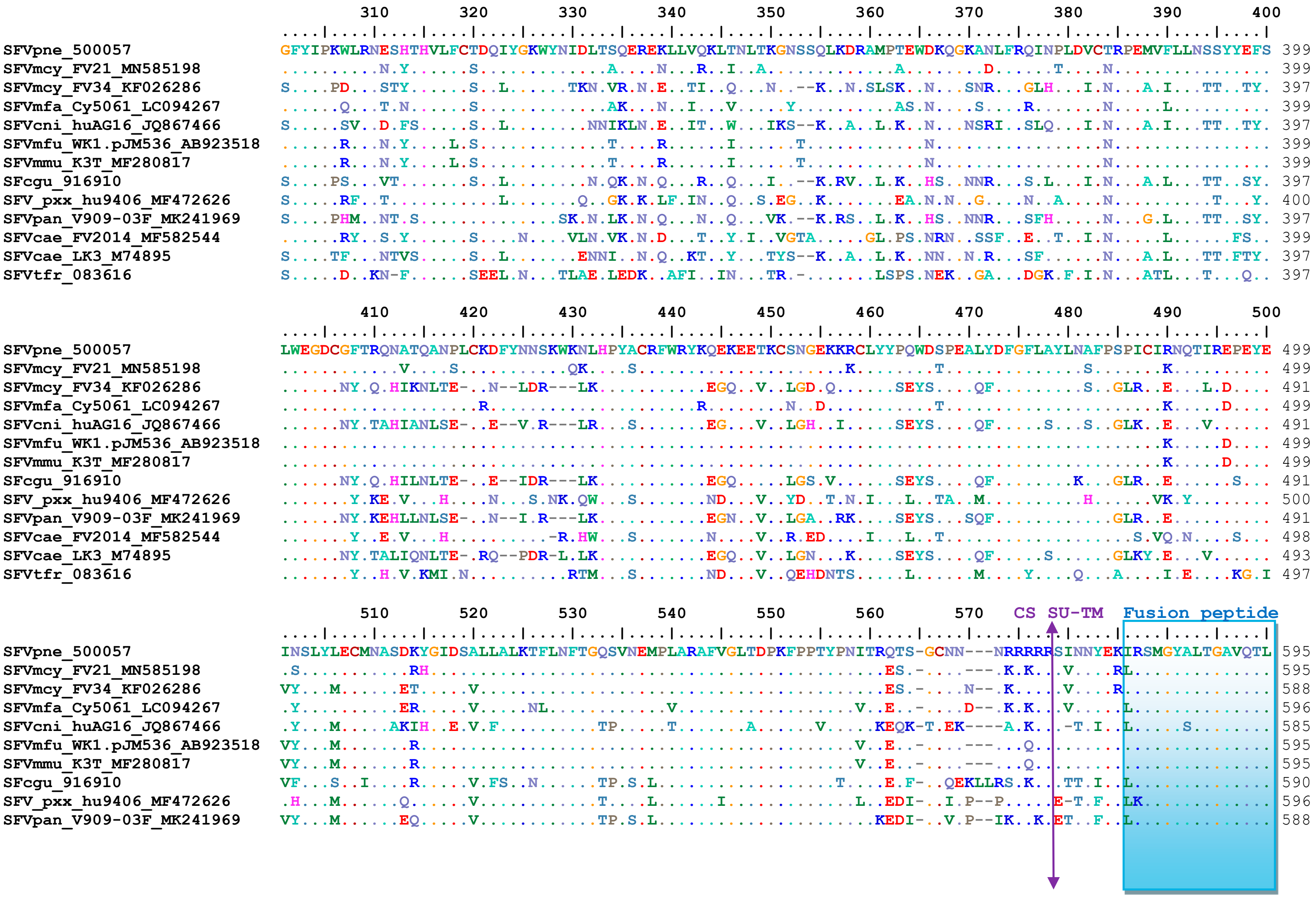
SFVpne_500057
SFVmcy_FV21_MN585198
SFVmcy_FV34_KF026286
SFVmfa_Cy5061_LC094267
SFVcni_huAG16_JQ867466
SFVmfu_WK1.pJM536_AB923518
SFVmmu_K3T_MF280817
SFcgu_916910
SFV_pxx_hu9406_MF472626
SFVpan_V909-03F_MK241969
SFVcae_FV2014_MF582544
SFVcae_LK3_M74895
SFVtfr_083616
CS
SU-TM
Fusion peptide

```
SFVcae_FV2014_MF582544      VH.I.M.....AK.HD...V.F..........TP.....T..............V...V..EQK-..ES----S.KK..-T.I..L.............. 592
SFVcae_LK3_M74895           VY...M....SAE......V.F..........TP....ST.....................KEQK-R...---LK..K..-T.I..L.....S........ 588
SFVtfr_083616               .Y...Q...L..ENRS.ENVIV...........P..D..S........Q.........W..EEDM..QS---.K.K...T.....L.........S.... 594


                                     610       620       630       640       650       660       670       680       690       700
                            ....|....|....|....|....|....|....|....|....|....|....|....|....|....|....|....|....|....|....|....|
SFVpne_500057               SQISDINDERLQQGVYLLRDHVVTLMEAALHDVSIMEGMLAIQH-VHTHLNHLKTMLLMRKIDWTFIKSDWIQQQLQKSEDEMKLIRRTAKSLVYYVTQT 694
SFVmcy_FV21_MN585198        ............H...............................-......................R..........TD..........R......... 694
SFVmcy_FV34_KF026286        ......................................V.....-......................R..........TD..........R......... 687
SFVmfa_Cy5061_LC094267      ............................................-......................Q......................R......... 695
SFVcni_huAG16_JQ867466      .........N...........I..........IT..........-.............L........R.N...E..........I............... 684
SFVmfu_WK1.pJM536_AB923518  ............................................-......................R................................ 694
SFVmmu_K3T_MF280817         ............................................-......................R................................ 694
SFcgu_916910                .........N....I.................IT..........-.....S.F........V....MDGN..KD...RT.....I............... 689
SFV_pxx_hu9406_MF472626     ................................I.L.......PTCAYLIF..F..L...........SGN...E......................A... 696
SFVpan_V909-03F_MK241969    A..........H..I...................L.........-.......F..............S..........T..................... 687
SFVcae_FV2014_MF582544      .........K.........N............IT..........-.....T..................N...E..........I............... 691
SFVcae_LK3_M74895           ...............S................IT..........-..........I.............N..KE....T.....I............... 687
SFVtfr_083616               .........N....I......I.......I..ITL.........-.......F....ID.R.....LQ.S.LKE..........M.K.........IK.. 693

                                     710       720       730       740       750       760       770       780       790       800
                            ....|....|....|....|....|....|....|....|....|....|....|....|....|....|....|....|....|....|....|....|
SFVpne_500057               SSSPTATSWEIGIYYEITIPKHIYLNNWQVINIGHLVESAGHLTHVKVKHPYEIINRECSDTQYLHLEECITEDYVICDIVQIVQPCGNSTEYSDCPVTA 794
SFVmcy_FV21_MN585198        .................V..............V...L...................K..............R.................A..L....... 794
SFVmcy_FV34_KF026286        .................V..............V...L..................................R.................T..L....... 787
SFVmfa_Cy5061_LC094267      ............V....I..................L...........N......................R.......V...........GT....... 795
SFVcni_huAG16_JQ867466      ............................................L...........K..NYE.........SQ.........V.S.......VS....... 784
SFVmfu_WK1.pJM536_AB923518  ................................V...........................N..........R..................E..L....... 794
SFVmmu_K3T_MF280817         ................................V...........................N..........R..................E..L....... 794
SFcgu_916910                ............V....V...........I......MQ.......I..Q.......K..TLSK........NQ.........E.L.....S.T....... 789
SFV_pxx_hu9406_MF472626     .................I.....F.....IF.............L..IQ.......K...EE.........SQ......K...MP.....SVT....... 796
SFVpan_V909-03F_MK241969    .K.A.............I.....F.....I..V...........LI..Q...V..K...IE.........NQ......V....P.....SIT....... 787
SFVcae_FV2014_MF582544      ............................................LI.....F.V..K..KYE......D..SQ......V....S......IT....... 791
SFVcae_LK3_M74895           ...T........................................LIR.....V..K..TYE......D..SQ......T....S......TT....... 787
SFVtfr_083616               Y....S.A....L...LI...........IT.V...I....Q..LI.IQ.......K..FEEK.....G..QL...V.......P......DS.....L. 793

                                     810       820       830       840       850       860       870       880       890       900
                            ....|....|....|....|....|....|....|....|....|....|....|....|....|....|....|....|....|....|....|....|
SFVpne_500057               IKIKSPYIQVSPLKNGSYLILSSTKDCSIPAYVPSVITVNETVKCFGVEFHKPLYAETKTSYEPQVPHLKLRLPHLTGIIASLQSLEIEITSTQENIKDQ 894
SFVmcy_FV21_MN585198        L.V.T..............V................V.....................................................V.......... 894
```

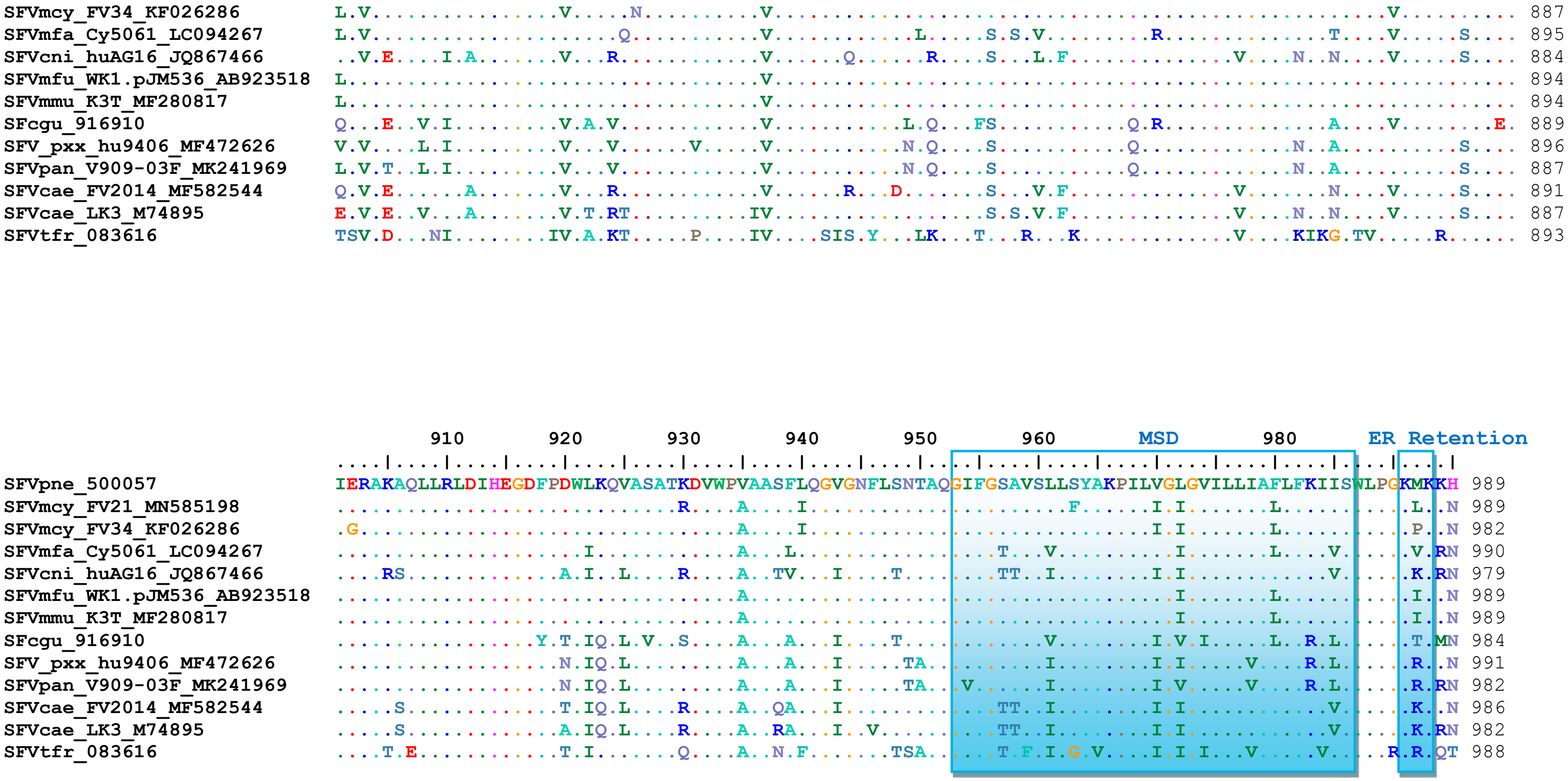
SFVmcy_FV34_KF026286 887
SFVmfa_Cy5061_LC094267 895
SFVcni_huAG16_JQ867466 884
SFVmfu_WK1.pJM536_AB923518 894
SFVmmu_K3T_MF280817 894
SFcgu_916910 889
SFV_pxx_hu9406_MF472626 896
SFVpan_V909-03F_MK241969 887
SFVcae_FV2014_MF582544 891
SFVcae_LK3_M74895 887
SFVtfr_083616 893
910 920 930 940 950 960 MSD 980 ER Retention
SFVpne_500057 IERAKAQLLRLDIHEGDFPDWLKQVASATKDVWPVAASFLQGVGNFLSNTAQGIFGSAVSLLSYAKPILVGLGVILLIAFLFKIISWLPGKMKKH 989
SFVmcy_FV21_MN585198 989
SFVmcy_FV34_KF026286 982
SFVmfa_Cy5061_LC094267 990
SFVcni_huAG16_JQ867466 979
SFVmfu_WK1.pJM536_AB923518 989
SFVmmu_K3T_MF280817 989
SFcgu_916910 984
SFV_pxx_hu9406_MF472626 991
SFVpan_V909-03F_MK241969 982
SFVcae_FV2014_MF582544 986
SFVcae_LK3_M74895 982
SFVtfr_083616 988

**Figure S5**

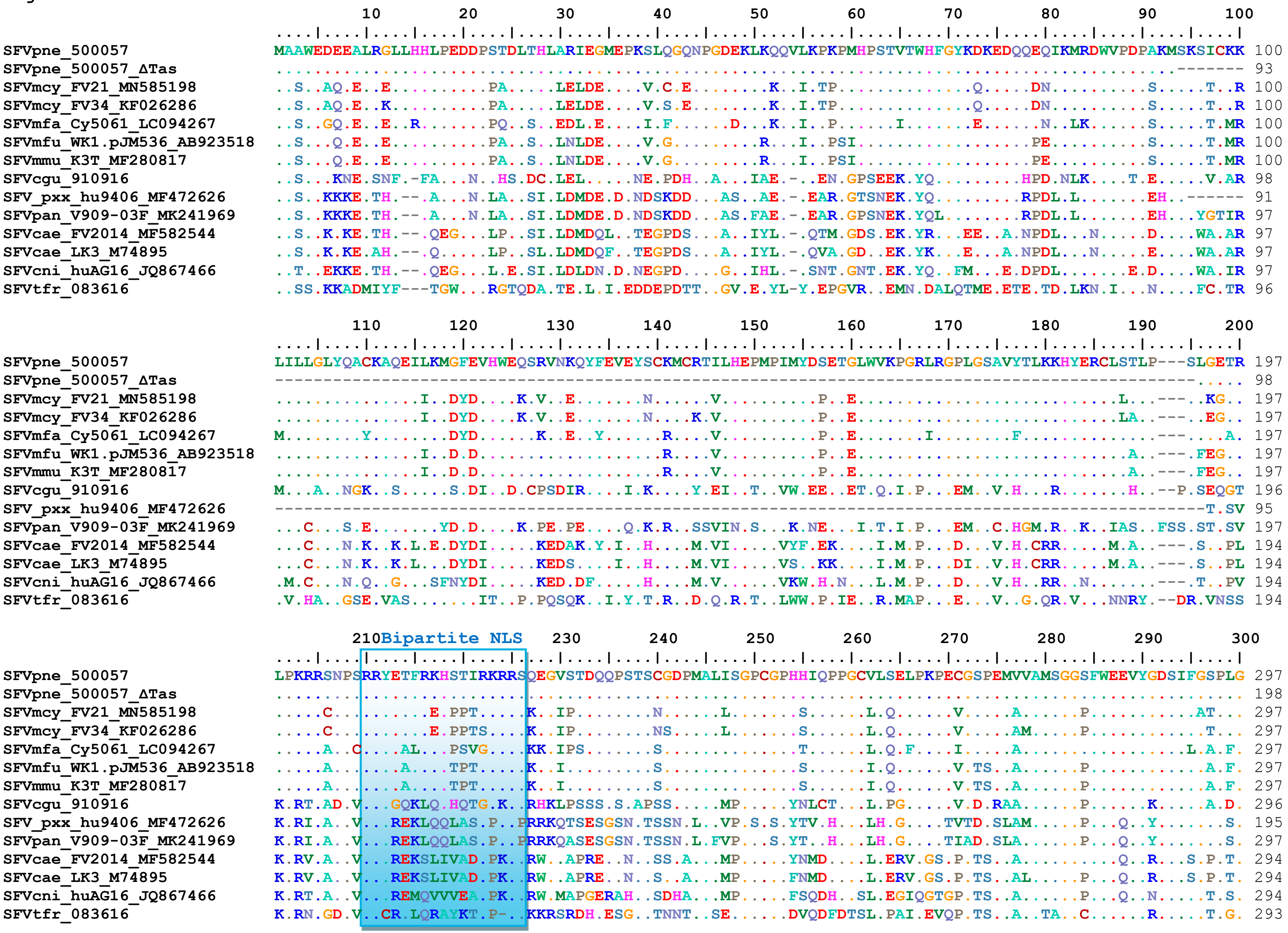

Bipartite NLS
SFVpne_500057
SFVpne_500057_ΔTas
SFVmcy_FV21_MN585198
SFVmcy_FV34_KF026286
SFVmfa_Cy5061_LC094267
SFVmfu_WK1.pJM536_AB923518
SFVmmu_K3T_MF280817
SFVcgu_910916
SFV_pxx_hu9406_MF472626
SFVpan_V909-03F_MK241969
SFVcae_FV2014_MF582544
SFVcae_LK3_M74895
SFVcni_huAG16_JQ867466
SFVtfr_083616

```
                                         310
                               ....|....|.
SFVpne_500057                  TGNDQLLSQFD 308
SFVpne_500057_ΔTas             ........... 209
SFVmcy_FV21_MN585198           SSE........ 308
SFVmcy_FV34_KF026286           S.E........ 308
SFVmfa_Cy5061_LC094267         ..E........ 308
SFVmfu_WK1.pJM536_AB923518     .SD........ 308
SFVmmu_K3T_MF280817            .SD........ 308
SFVcgu_910916                  S..HNM.---- 303
SFV_pxx_hu9406_MF472626        S..S------- 199
SFVpan_V909-03F_MK241969       S.DN------- 301
SFVcae_FV2014_MF582544         SS--------- 296
SFVcae_LK3_M74895              PSEN------- 298
SFVcni_huAG16_JQ867466         S..N------- 298
SFVtfr_083616                  VSDY.M.---- 300
```

Figure S6

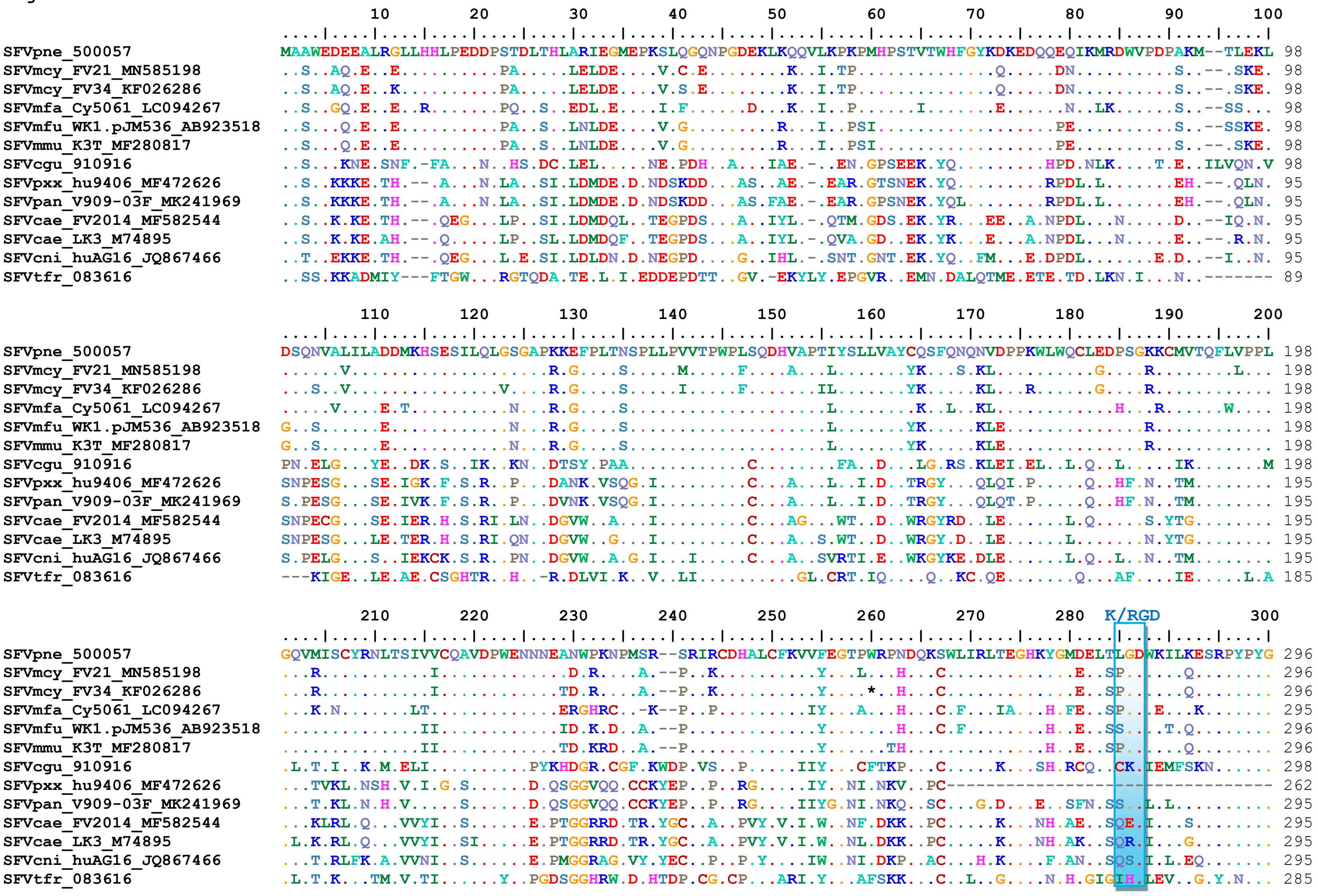

SFVpne_500057
SFVmcy_FV21_MN585198
SFVmcy_FV34_KF026286
SFVmfa_Cy5061_LC094267
SFVmfu_WK1.pJM536_AB923518
SFVmmu_K3T_MF280817
SFVcgu_910916
SFVpxx_hu9406_MF472626
SFVpan_V909-03F_MK241969
SFVcae_FV2014_MF582544
SFVcae_LK3_M74895
SFVcni_huAG16_JQ867466
SFVtfr_083616
K/RGD

```
                                   310       320       330       340       350       360       370       380       390       400
                            ....|....|....|....|....|....|....|....|....|....|....|....|....|....|....|....|....|....|....|....|
SFVpne_500057               PVGKCSRLQYAVSVKMKVIGGPLTSRVLALKALNFHRMNICNMD-NPGIGEGHPPHGYSHAIKAYGPQYGSCEERVWQVATKCIGIKEEDYWCEYDHRGY 395
SFVmcy_FV21_MN585198        .I.EDPN.....G............T.......S...V......-..SL....A.L.....L................T......PE..N.........F 395
SFVmcy_FV34_KF026286        .I.EDPY.....G.....T......T................I.-..SL......L.....L................T......P...N.........F 395
SFVmfa_Cy5061_LC094267      .I.E.PN.....G.....T.....PK.......S...V......-.......Y.AQ.....L....A...CS......TGV.L..GQ.K........H.. 394
SFVmfu_WK1.pJM536_AB923518  ...EDPN...........T......T.......C...V......-...L......L.....L................A......PG.G........... 395
SFVmmu_K3T_MF280817         ...EDPN...........T......T.......C...V......-...L......L.....L................A......PG.G........... 395
SFVcgu_910916               KK.E.AA....IQA..H.Q.A.....M.D....S.L.AQ...L.-.....Q.NMAT.FTPGL....S.L.DSV....WT..RI..P...H..K.CQEH.. 397
SFVpxx_hu9406_MF472626      ---------------------------------------------------------------------------------------------------- 262
SFVpan_V909-03F_MK241969    KP.ED.L.....Q.....T.A..DPL.M.I...W.Q.AL..EPE-.S.....KK.S..TQ.LL...S.L.CS.....LT..GQ.KPD.LA..KYK.QYFM 394
SFVcae_FV2014_MF582544      SI.Q.AK.....Q...R.DKA...AK..SI...H...W...QPE-.......YS.S..TQ.L......H..V.....LTS.RMT.PQ.D...KDAYRW.. 394
SFVcae_LK3_M74895           LI.Q.PK....IQ...R.DKA...AK...V...H...W...QRE-.......YF.S..TQ.L......H..A.Q...LIS..IV.PQ.K...RDAYRW.. 394
SFVcni_huAG16_JQ867466      RI.E.PK.....Q...RTAKA..S.K.R.I.......W...QPE-.......FS.S..TQ.L......R..V.....LT...M.KPQ.DA..KD.WKY.. 394
SFVtfr_083616               DK...PM.....E..LA.Q.S...DL...Q...S...L.M..VQK...L...KIAA...E.LL........SM....WIT..I..ANVD...KPWGEYEL 385

                                   410       420       430       440       450       460       470       480       490       500
                            ....|....|....|....|....|....|....|....|....|....|....|....|....|....|....|....|....|....|....|....|
SFVpne_500057               --FPHIPNKMSPTWVRHVAPCNIQRFATPHDLQDLANELLPSGFSITTPEGNCYTSSRRLHYGNEGTLQEFQDDYNRIKRKYQNG----SSSEESDED-- 487
SFVmcy_FV21_MN585198        --..MV...L.......A..YC.......Y...YF......P.......K.VS...D.............YNENCDKV..G.DEI----...DY....-- 487
SFVmcy_FV34_KF026286        --..MV.G.L.......A.SYQ.......Y...YI......P.......K.VN...D.K...........YNENCDK...G.DEI----...DY....-- 487
SFVmfa_Cy5061_LC094267      --..VV.QPL....A..A..YG.......Y...KF.S....Y....N..T.A..V.D....P........YLGNIEQTR.G.DEI----L..DD....-- 486
SFVmfu_WK1.pJM536_AB923518  --..I....L.......A..YG...L...Y...MF......P....N..S.A..I...............Y.ENCD....G.EDI----...DS....-- 487
SFVmmu_K3T_MF280817         --..I....L.......A..YG...L...Y...MF......P.Y..N..S.T..V.N.............Y.ENCD....G.EDI----...DS....-- 487
SFVcgu_910916               --..LV.DPKK......L..FKVTP.T..V...KIV..S..YQYG.I.RD.KI.K.EKKDNLFKY...E.YW.NEEET.KQFNCDWLDSD.DDY.G..-- 493
SFVpxx_hu9406_MF472626      ---------------------------------------------------------------------------------------------------- 262
SFVpan_V909-03F_MK241969    --W.LV...H..G.L..AVRHRLS....LT...KV.D....K.Y..E..D.IKV..N.KV..AV....K.YYAKMSE.EK..GG.----L..NS-.T.YS 487
SFVcae_FV2014_MF582544      --..LV...HH.G.T.YLTKFK.S..S..A.I.KITD....R.A..V.AD.SK.E.T.KV.LV.....K.Y..KNRE.EE..GC.CC--L..DSD.D.YS 490
SFVcae_LK3_M74895           --..LV...HH.G.T..LTKFK.S.....A.I.KIVD....R.A..V.AD..R.E.T.KV.LV.....E.Y.AKIRK----------------------- 469
SFVcni_huAG16_JQ867466      --..LV...QD.G.T..LTKFK.S..S..A.I.KITD....K.AC.M.TD.TK.I.T.KV.LV.....E.YKARCKE.EE..GGH----L..DS--D.YS 486
SFVtfr_083616               DD..VV...HKV..LA.TPQIKLSP....S.V..YV.LT..ENYKVV..S.EE.V.N.KVNIVRDL.WS.IKASKFKNDWR.I------ADDDNPAD.W- 478

                                   510
                            ....|....|....
SFVpne_500057               -------------- 487
SFVmcy_FV21_MN585198        -------------- 487
SFVmcy_FV34_KF026286        -------------- 487
SFVmfa_Cy5061_LC094267      -------------- 486
SFVmfu_WK1.pJM536_AB923518  -------------- 487
SFVmmu_K3T_MF280817         -------------- 487
SFVcgu_910916               -------------- 493
SFVpxx_hu9406_MF472626      -------------- 262
SFVpan_V909-03F_MK241969    EDTPD-------ED 494
SFVcae_FV2014_MF582544      EDTPDTESTSVEED 504
```

```
SFVcae_LK3_M74895          --------------- 469
SFVcni_huAG16_JQ867466     EDTPATETTEVEED 500
SFVtfr_083616              --------------- 478
```
